# Melatonin Signaling Pathways Implicated in Metabolic Processes in Human Granulosa Cells (KGN)

**DOI:** 10.3390/ijms23062988

**Published:** 2022-03-10

**Authors:** Arjoune Asma, Sirard Marc-André

**Affiliations:** 1 Centre de Recherche en Reproduction, Développement et Santé Intergénérationnelle, Département des Sciences Animales, Faculté des Sciences de l’Agriculture et de l’Alimentation, Université Laval, Quebec, QC G1V 0A6, Canada; asma.arjoune.1@ulaval.ca; 2 Department of Animal Production, National Agronomic Institute of Tunisia, University of Carthage, Mahrajène 1082, Tunisia

**Keywords:** melatonin, granulosa cells, signaling pathways

## Abstract

Female reproduction depends on the metabolic status, especially during the period of folliculogenesis. Even though it is believed that melatonin can improve oocyte competence, there is still limited knowledge of how it can modulate metabolic processes during folliculogenesis and which signaling pathways are involved in regulating gene expression. To investigate the effects of melatonin on metabolic signals during the antral stage of follicular development, human granulosa-like tumor cells (KGN) were treated with melatonin or forskolin, and gene expression was analyzed with RNA-seq technology. Following appropriate normalization and the application of a fold change cut-off of 1.5 (FC 1.5, *p* ≤ 0.05), 1009 and 922 genes were identified as differentially expressed in response to melatonin and forskolin, respectively. Analysis of major upstream regulators suggested that melatonin may activate PKB/mTOR signaling pathways to program the metabolism of KGN cells to support slower growth and differentiation and to prevent follicular atresia. Similarly, PKA activation through stimulation of cAMP synthesis with FSK seemed to exert the same effects as melatonin in reducing follicular growth and regulating differentiation. This study suggests that melatonin may act through PKA and PKB simultaneously in human granulosa cells to prevent follicular atresia and early luteinization at the antral stage.

## 1. Introduction 

Female reproduction is sensitive to the metabolic status, particularly nutrition intake during gestation, but also in the period of gametogenesis [1]. In mono-ovulatory species, one single oocyte, usually from the largest growing follicle, is ovulated at each cycle. This process is well established and requires close coordination between the oocyte and the surrounding somatic cells, such as granulosa cells. Gap junctional channels mediate the transfer of metabolites, ions, and signaling molecules between these cells [2]. These metabolic interactions between the oocyte and granulosa cells have long been studied since they are involved in the control of meiotic resumption [3,4].

The oocyte acquires its maturation competence during folliculogenesis, when the bidirectional communication between somatic cells and the oocyte creates an intrafollicular microenvironment that maintains the growth of primordial follicles into antral follicles, from which one is selected to ovulate a high-quality oocyte. This microenvironment depends on the maternal metabolic environment, which can affect the oocyte epigenetic and metabolic programming throughout follicular growth [5].

Over the last few years, studies have shown melatonin (N-acetyl-5-methoxytryptamine), a neurohormone secreted by the pineal gland, controls the daily coordination of physiological, neuroendocrine, and behavioral processes. Most tissues, including ovaries, can synthesize melatonin in their mitochondria. Melatonin is expressed in oocytes, cumulus, and granulosa cells, where it acts through several molecular cascades via its specific receptors [6,7]. It is also well known that melatonin, at different doses, can modulate folliculogenesis, steroidogenesis, oocyte maturation, and can also improve oocyte competence [8]. Recently, researchers identified melatonin as a metabolic programmer in mammalian cells since a small disruption in its secretion rhythm led to metabolic syndromes, such as obesity and diabetes [9,10]. In addition to its effects on cancer cells, which are well studied, it was suggested that melatonin controls nutrient metabolism to limit cancer cell proliferation and progression [11].

There is very little knowledge of how melatonin regulates and controls metabolic processes during follicular growth and development and of which potential signaling pathways are used by melatonin to trigger specific gene expression events. A few studies examined the effects of melatonin on oocyte metabolic programming and showed that supplementation of porcine oocyte culture medium can affect lipid metabolism and modulate mitochondrial metabolic functions [12,13], but the underlying processes have not been investigated. 

The overall objective of this study was to investigate the effects of melatonin on metabolic signals during the antral stage of follicular development as assessed by transcriptomic analysis. The human granulosa-like tumor cell line (KGN), which originated from a diagnosed stage-3 granulosa cell tumor from an antral or pre-antral stage follicle of a Japanese woman in 1984, was selected as a model for this study [14]. By using the KGN cell line, controlled and very specific conditions could be established repeatedly, which is not possible with primary cells. The KGN cells were treated with melatonin or forskolin, and the results of RNA-seq and genomic analysis provided new insights into the melatonin-induced molecular pathways implicated in the metabolic processes of the dominant follicle.

## 2. Results

### 2.1. RNAseq Data Analysis

Gene Expression Analysis

Following the appropriate standardization and the application of differential cut-offs for each treatment, 1009 and 922 differentially expressed genes were observed in response to the melatonin and FSK treatments vs. control, respectively, at a *p*-value of 0.05. A full list of DEGs for each treatment is available in supplemental data (Supplemental Tables S1 and S2).

The Ingenuity Pathway Analysis^®^ (IPA) software was used to identify the principal canonical pathways and upstream regulators associated with the observed DEGs in each treatment (Supplemental Table S3). The IPA also predicted upstream regulators most likely activated (z-score ≥ 2) or inhibited (z-score ≤ −2) and highlighted the signaling pathways involved in each treatment. This functional analysis enabled us to use the combination of the information revealed by the gene expression datasets and the knowledge extracted from the literature to derive the underlying reasons for the observed transcriptional changes and to predict likely results [15]. Upstream regulatory identity inference is an essential way of providing biological insight into the observed change in gene expression in response to each treatment.

### 2.2. Upstream Regulators of Melatonin Effects

The major upstream regulators of MLT-induced gene expression change in our experiment are shown in Table 1. Death-associated protein 3, also known as a mitochondrial ribosomal protein (DAP3); mitochondrially encoded tRNA-glutamic acid (MT-TE); and mitochondrial Lon protease (LONP1) are all related to mitochondrial ribosome biogenesis and mitochondrial proteins synthesis. The DAP3 is a highly conserved GTP pro-apoptotic protein, and MT-TE is involved in the oxidative phosphorylation by maintaining the assembly of respiratory chain proteins. Moreover, storkhead box 1 (STOX1) was also a major upstream regulator in this treatment. It regulates metabolism, response to hypoxia, redox balance, and nitric oxide [16,17] via the AKT signaling pathway. Actinonin, also identified as a major upstream, is an antibiotic that induces in vitro mitophagy via a specific mitochondrial ribosomal and RNA decay pathway [18]. Furthermore, hypoxia-inducible factor 1-alpha (HIF1α), a transcription factor activated under hypoxic conditions, was a major upstream in this treatment. Mitochondrial assembly of ribosomal large subunit 1 (MALSU1, also known as C70rf30) is a member of the large family of DUF143 proteins. The 39S ribosomal protein L14, mitochondrial (MRPL14) was also identified as a major upstream in this treatment. It maintains mitochondrial translation and mitochondrial ribosome biogenesis [19] to maintain oxidative phosphorylation (OXPHOS) and protein synthesis. The transcription factor AP-2 Alpha (TFAP2A) is involved in lipid homeostasis, regulation of lipid droplets biogenesis, and cholesterol storage in response to Wnt-stimulation. It was also identified as an upstream regulator [20]. Besides, the gene SIRT3, which encodes for NAD-dependent deacetylase sirtuin-3, located in the mitochondrial matrix, was identified by IPA upstream analysis as a major upstream factor in this treatment. It is an important downstream mediator of melatonin, involved in a variety of cellular events, including fatty acid and acetyl-CoA metabolism, endogenous antioxidant defense, and electron transport chain function [21].

### 2.3. Upstream Regulators of FSK Effects

Forskolin was used to identify the effects of melatonin linked to PKA activation in granulosa cells. Among the upstream regulators highlighted by IPA in the FSK treatment (Table 2), the gene Zinc Finger and BTB Domain Containing 17 (ZBTB17, known also as MIZ-1) encodes an MYC-interacting Zinc-Finger protein. Vascular endothelial growth factor A (VEGF), prostaglandin E receptor 2 (PTGER2), tumor protein p53 (TP53), and hepatocyte growth factor (HGF) were also identified as upstream regulators. Cyclin-dependent kinase inhibitor 1A (CDKN1A), also named p21, represents a major target of p53 activity and thus is associated with linking DNA damage to cell cycle arrest. Forkhead box M1 (FOXM1), nuclear receptor subfamily 1 group H member 3 (NR1H3), amphiregulin (AREG), E2F transcription factor 4 (E2F4), transcription factor 3 (TCF3), and transcription factor 4 (TCF4) were also identified as important upstream signaling regulators in this treatment as well. Almost all these upstream regulators are involved in granulosa cell growth and differentiation. Contrarily to the melatonin treatment, the most significant upstream regulators highlighted in this treatment were related to glucose uptake, angiogenesis, lipogenesis, and oxidative phosphorylation functions, and most were inhibited according to their z-scores.

Interestingly, in addition to the activation of PKA and PKC signaling pathways, some predicted regulators known to be PKB downstream targets, such as tumor protein 53, forkhead boxO3, and P38 MAP kinase (MAPK1, MAPK7, MAPK8, MAPK9, and MAPK14, AKT), were also identified in the FSK treatment. Vascular endothelial growth factor A (VEGFa), a major upstream regulator, was inhibited (z-score = −2.090) following PKA stimulation. Studies showed that inhibition of VEGF stimulates fatty acid oxidation and decreases glucose uptake and anaerobic glycolysis in endothelial cells [22]; however, FGF2 was also inhibited (z-score = 1.436) in the FSK treatment. 

Tumor proteins TP53 and TP73 were scored as activated in the FSK treatment. The TP53 regulates glucose metabolism by repressing the expression of glucose transporters and downregulating rate-limiting enzymes of glycolysis. It activates the pyruvate dehydrogenase complex (PDH) to reduce the conversion of pyruvate to lactate and to increase the conversion of pyruvate to acetyl-CoA to maintain oxidative phosphorylation. Besides, TP73 is a stimulator of glutamine uptake to replenish the tricarboxylic acid (TCA) cycle by decreasing the expression of the transcription factor MYC, which was inhibited in our treatment (z-score = −2.976). In turn, this gene is responsible for suppressing the mRNA expression of the glutamine uptake receptor SLC1A5 [23]. HGF was scored as inhibited by IPA upstream analysis (z-score = −3.168). Erb-b2 receptor tyrosine kinase 2 (ERBB2), a member of the epidermal growth factor receptor (EGFR) family, and AREG were also inhibited following PKA stimulation. Studies showed that inhibition of these genes stimulates glucose deprivation and downregulates glycolysis and lipogenesis pathways [24,25].

### 2.4. Major Canonical Pathway

To obtain a biological interpretation of the combination of differentially expressed genes, we used IPA to highlight the functions and pathways affected in each treatment. A list of canonical pathways and the principal putative regulators that were more significant to the input data set was identified by IPA (Table 3).

## 3. Discussion

Granulosa cells are specialized cells that act as a barrier to protect the oocyte against the extra ovarian microenvironment by metabolic and signaling interplay. An increased understanding of their roles in metabolism and reproduction continues to be instrumental in the improvement of infertility prevention methods and treatments to ameliorate IVF outcomes. During the final stages of maturation, granulosa cells supply the growing follicle with energy substrates to produce the necessary ATP for the oocyte to reach the preovulatory stage. This process is highly regulated by the metabolic and signaling coupling between cumulus and granulosa cells and between cumulus cells and the oocyte.

The use of the KGN granulosa cell line as opposed to in vivo granulosa cells or primary cultures gave us the possibility to repeatedly establish very specific and controlled conditions that allowed us to explore the molecular pathways involved in the regulation of granulosa cell metabolism by melatonin. The metabolic profile of granulosa cells obtained from growing follicles seemed to resemble the profile of highly proliferating cells, such as cancer cells and pre-implementation embryonic cells [26]. While normal cells rely on oxidative phosphorylation to produce ATP, most highly proliferating cells, such as follicular granulosa cells, rely on aerobic glycolysis (Warburg effect) [27]. To the best of our knowledge, this study is the first to attempt to study the effects of melatonin on the metabolism of human granulosa cells and to identify the signaling cascades involved.

### 3.1. Melatonin Balances Oxidative Phosphorylation and Anaerobic Glycolysis Using Different Signaling Networks in Human Granulosa Cells

It is well known that reproduction and metabolism are interconnected and reciprocally regulated. Several studies showed that female fertility depends on the maternal metabolic environment, which affects the intrafollicular microenvironment and consequently oocyte quality [28,29]. The effects of melatonin on porcine oocyte metabolism were recently studied. While one study found that this hormone could maintain lipid homeostasis by stimulation of lipogenesis, lipolysis, fatty acid β-oxidation, and mitochondria biogenesis [12], another study, however, showed that melatonin inhibited mitochondrial functions and that the nucleotide salvage pathway may be the compensatory path to provide the necessary energy (ATP) to the oocyte [13]. To date, no previous studies attempted to demystify the molecular pathways implicated in the effects of melatonin on the metabolism of human granulosa cells.

This study revealed key target genes implicated in the downstream signaling network used by melatonin to exert its function of “metabolic programmer” in human granulosa-like cells (KNG). Some reports claimed that the effects of melatonin on the modulation of many reproductive functions are transduced by two G-protein-coupled receptors, MTNR1A and MTNR1B, which were detected in human granulosa cells [30] The signaling mediated by these receptors affected cellular metabolism and also led to the scavenging of reactive oxygen and nitrogen species [31,32]. Stimulation of melatonin receptors decreased cAMP (cyclic adenosine 3′,5′-monophosphate) levels, which led to inhibition of protein kinase A (PKA) and CREB phosphorylation but promoted cGMP synthesis (cyclic guanosine 5-monophosphate) [33,34]. Some studies reported that melatonin acts through the PKC signal pathway [35], while other studies suggested that it acts by triggering crosstalk between the cAMP/PKA and PLC/PKC signal pathways [36].

In line with these findings, our data confirmed that melatonin is a regulator of the granulosa cell metabolism-related factors DAP3, MT-TE, LONP1, MALSU1, and MRPL14, which are mitoribosomal proteins involved in the regulation of mitochondrial translation, mitochondrial ribosome biogenesis, mtDNA metabolism, and cell-stress response. Moreover, the most significant upstream regulators in this treatment, DAP3, LONP1, and STOX1, are PKB signaling downstream factors. DAP3, a substrate of Akt kinase, has a dual function in both mitochondrial physiology and cell death regulation [37], but its proapoptotic effect could be nullified by PKB activation [38]. This factor was inhibited by melatonin in our data, which suggest that this hormone may prevent follicular atresia at the dominant follicular stage by inhibiting apoptosis and mitophagy in human granulosa cells of dominant follicles through activation of the PKB/Akt pathway. The strong activation of the antiproliferative antibiotic actinonin, an aminopeptidase inhibitor that inhibits tumor cell growth through MAPK and WNT signaling pathways [39,40], suggests that melatonin kept granulosa cells of antral follicles in a cell growth deceleration phase by stalling mitochondrial protein synthesis and mitochondrial reticulum fragmentation [41]. Nevertheless, the antiangiogenic role of actinonin was previously highlighted [42]. The ability of melatonin to inhibit granulosa cell proliferation and angiogenesis could be part of the mechanism driving granulosa cell early differentiation by highlighting beta-catenin (CTNNB1) expression in our results. The CTNNB1 protein is an estrogen synthesis regulator and a key factor of the Wnt signaling pathway [43].

Our results confirmed that melatonin plays an essential role in controlling human granulosa cell metabolism homeostasis and apoptosis under hypoxia conditions. The actions of melatonin are mediated via HIF1α and HIF2α (EPAS1) expression, transcription factors expressed in response to low cellular oxygen levels in granulosa cells of numerous species [44,45]. In the follicular fluid of the human ovary, as folliculogenesis progresses, the oxygen tension diminishes [46]. Moreover, the activity of hypoxia-inducible factor 1-alpha (HIF1α) increased in ovulating and differentiating follicles [47,48]. It is a master activator of glycolysis [49] that orchestrates the switch from oxidative to glycolytic metabolism to produce the required energy (ATP) for cells under hypoxia conditions [50]. Nevertheless, HIF1α is a significant regulator of ovarian gene expression and plays an essential role in ovulation. It is believed that HIF1A expression, in response to gonadotropins hCG and FSH, activates downstream processes of steroidogenesis, cell proliferation, angiogenesis, and inflammation in granulosa cells, all processes that are critical for ovulation [51,52]. In vivo studies showed that HIF1α is critical for follicle rupture in mice [53], while some authors found that HIF1α can also be implicated in the suppression of cellular proliferation when cells do not have an adequate supply of energy sources, such as ATP, under hypoxia conditions [54]. As described in recent studies, hypoxia induces Akt accumulation in mitochondria, which in turn stimulates tumor metabolic reprogramming-related factors, such as HIF1-2α through PI3K/Akt/mTOR signal pathway activation and LKB1/AMPK and cMYC downregulation [55,56].

**Figure 1:**
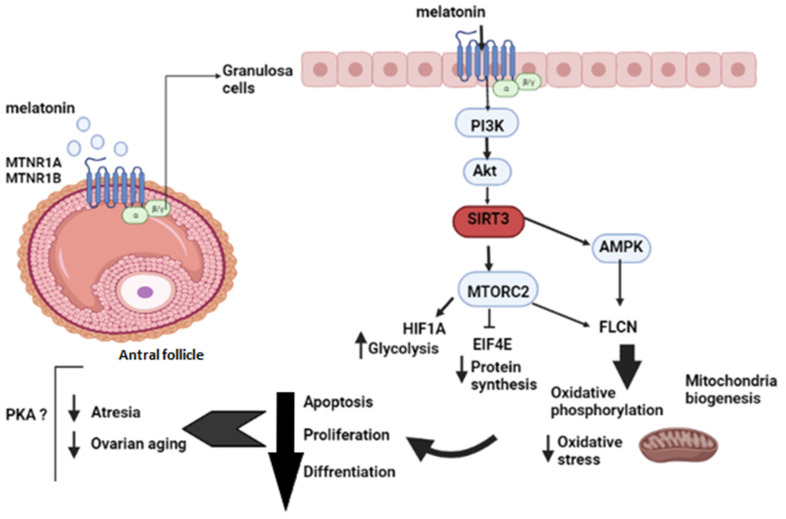
Melatonin effects on follicular development at the antral stage.

The expression of aging-related sirtuin genes in mural granulosa cells and KGN cells is well documented [57,58]. The most studied mitochondrial sirtuin, SIRT3, catalyzes post-translational modifications of proteins and was activated by melatonin in our data. It is implicated in ATP production, β-oxidation, reactive oxygen species regulation, apoptosis, and ketogenesis [57,58,59]. It has been mentioned that SIRT3 plays an essential role in folliculogenesis and luteinization and may act directly through activation of mitochondrial proteins involved in OXPHOS and TCA and indirectly by activating peroxisome proliferator-activated receptor-gamma coactivator 1α (PGC-1α) and AMP-activated protein kinase [60]. However, PGC1β, which stimulates mitochondrial biogenesis and function, was downregulated in our data [61]. Moreover, SIRT3 may act, in response to melatonin, like a guardian of the redox state and steroidogenic metabolism in granulosa cells by activating FoxO3a nuclear translocation, which stimulates antioxidant gene transactivation [62]. SIRT3 mediates metabolic reprogramming via destabilizing HIF1α and the expression of its target genes that are involved in anaerobic glycolysis and by inhibiting the mTOR signaling pathway, which is involved in follicle reserve regulation in response to nutrient status [63]. Strangely, Rictor, a subunit of mTORC2 and a novel binding partner in the mTOR pathway, had a strong tendency toward activation in our data. Although its role in ovarian cells has not been well studied, it may be activated by growth factors, and it was suggested that it could be implicated in granulosa cell growth, proliferation, and metabolism [64]. Indeed, Rictor can be involved in the control of follicular reserve pool by slowing the growth of follicles and preserving their viability [65,66]. Some authors suggested that mTORC2 regulates both aerobic glycolysis and OXPHOS through the insulin-Akt pathway [67], activation of the AMPK pathway [68], and possibly through the PKA and PKC pathways to maintain cytoskeletal remodeling and cell survival [69]. Besides, mTORC2 regulates protein synthesis via controlling many genes involved in transcription, such as initiation and elongation factors, ribosomal proteins, and rRNA [70]. Since the translation initiation factor EIF4E was inhibited in our data, it could indicate that melatonin inhibits protein transcription through the mTORC2/PKB signaling pathways. Melatonin may be involved, through SIRT3 activation of Rictor expression, in the balance between OXPHOS and glycolysis to provide energy and between mitochondrial fusion and fission to control cell proliferation and follicular aging (Figure 1).

Folliculin (FLCN) is a tumor suppressor causing the Birt–Hogg–Dubé syndrome and is involved in energy homeostasis regulation by controlling several metabolic pathways. Its roles in several organ cells are well studied; however, to the best of our knowledge, FLCN expression in human granulosa cells was reported in our data for the first time. It is known that the transcriptional activity of TFE3, a member of the MiTF/TFE transcription factor family, is negatively regulated by FLCN, which was confirmed in our data. The knockdown of FLCN in salivary glands and cystic kidneys triggered metabolic reprogramming pathways by modulating PGC1α and TFE3 transcription and the AMPK-mTOR pathway to support cell proliferation [71,72]. Loss of FLCN also upregulated glycolysis, glycogen synthesis, and ATP production [73]. Melatonin may upregulate FLCN expression in human granulosa cells at the antral stage to slow cell cycle progression and to decrease granulosa cell proliferation.

Melatonin seems to be implicated in regulating steroid receptors in human granulosa cells The G protein-coupled estrogen receptor 1 (GPER1, also named GPR30) is expressed in KGN cells [74] and had a strong tendency to be activated in our data (z=1.242). This receptor plays an important role in cell proliferation and differentiation, and it is also involved in cellular metabolism in response to estrogens. It stimulates cAMP accumulation and PKA activation to maintain meiotic arrest during the later stages of zebrafish oocyte development to prevent maturation [75]. Moreover, it participates in cell cycle arrest and angiogenesis inhibition through activation of the Erk1/2 pathway [76,77]. Interestingly, prohibitin 2 (PHB2), a repressor of estrogen receptor activity, was also activated by melatonin in our results. Studies showed that this protein is regulated in granulosa cells via MEK1 and p38 MAPK to maintain differentiation and survival [78]. These findings indicate, for the first time, that melatonin may reduce cell cycle progression and steroid production to prevent early differentiation of granulosa cells. In summary, our study suggests that melatonin may activate PKB signaling in KGN cells to regulate upstream factors related to mitochondrial biogenesis, anaerobic glycolysis, and OXPHOS. Melatonin downstream activity could also involve other signaling pathways, such as mTOR and AMPK, to reduce granulosa cell proliferation, prevent early differentiation, and promote survival (Figure 1).

### 3.2. FSK Effects on KGN Granulosa Cells

The effects of melatonin are mediated through specific receptors, which activate numerous signaling pathways, such as the PKA pathway, in several tissues [36,37,38,39,40,41,42,43,44,45,46,47,48,49,50,51,52,53,54,55,56,57,58,59,60,61,62,63,64,65,66,67,68,69,70,71,72,73,74,75,76,77,78,79,80]. Therefore, forskolin was used to identify the effects of melatonin linked to PKA activation. Vascular endothelial growth factor (VEGF), a proangiogenic factor essential for dominant follicle growth and development, was the most significant upstream regulator inhibited according to IPA in the forskolin treatment. Strangely, VEGF was reported to be upregulated in KGN cells after cAMP stimulation [81,82]. Decreasing VEGF (or VEGFa) expression downregulated the PI3K/Akt pathway and reduced glucose uptake and glycolysis in peripheral organs [83]. It potentiated FSH effects in inducing proliferation and survival in bovine and porcine granulosa cells through the ERK1/2 pathway and in regulating follicular cell steroidogenesis at a later stage of follicle differentiation [82,83,84,85,86]. Prostaglandin E Receptor 2 (PTGER2), the second major upstream factor in the FSK treatment, was inhibited according to IPA analysis. Strangely, PTGER2 is known as an adenylate cyclase-coupled receptor that increases cAMP and protein kinase A signaling and is implicated in follicle rupture, ovulation, and fertilization processes [87]. It mediates the effects of prostaglandins on progesterone production in human granulosa-lutein cells [88]. FSK may prevent premature differentiation of granulosa cells by suppressing progesterone production and stimulating fibroblast growth factor 2 (FGF2), a well-known luteal proangiogenic factor, which is stimulated by elevated PGE2 levels during the periovulatory period in bovine granulosa cells [89].

The cell cycle inhibitors TP53 and CDKN1A, which are related to granulosa cell differentiation, were among the upstream regulators identified by IPA and had a strong tendency to be activated through PKA activation in the FSK treatment. Characterized by its ability to induce cell cycle arrest, apoptosis, and senescence, TP53 was also reported to play an important role in metabolic regulation [90] through the maintenance of mitochondrial oxidative phosphorylation to promote fatty acid β-oxidation [91]. In granulosa cells, TP53 appeared to control steroidogenesis, follicle atresia, and remodeling [92]. It was also previously described as an activated upstream regulator in response to PKB and PKC activation in granulosa cells from follicles at the plateau phase with a reduced cell growth rate [43,44,45,46,47,48,49,50,51,52,53,54,55,56,57,58,59,60,61,62,63,64,65,66,67,68,69,70,71,72,73,74,75,76,77,78,79,80,81,82,83,84,85,86,87,88,89,90,91,92,93,94].

Hepatocyte growth factor (HGF) was highlighted as an inhibited upstream gene by IPA in response to PKA activation by FSK in agreement with previous reports from our group [95]. This growth factor, which was initially characterized as a mitogen for mature hepatocytes via the tyrosine kinase receptor c-Met, is now known to act on a wide variety of cells, predominantly epithelial and endothelial cells. Previously, HGF was described as an essential factor in the inhibition of differentiation and the stimulation of proliferation of granulosa cells by decreasing progesterone production and aromatase activity [96]. These results were confirmed by another study with KGN cells, which concluded that HGF, via activation of the MAPK pathway, may suppress progesterone production, thereby stimulating granulosa cell proliferation and preventing premature differentiation [97]. These studies confirmed the link between LH stimulation and the role of HGF in granulosa cells. Based on our results, we suggest that HGF promotes the differentiation/maturation process in granulosa cells, but its action is downregulated in response to PKA activation.

Adenylate cyclase activation regulates the expression of several cell proliferation and cell cycle genes, such as FOXM1, CCDN1, CDKN2A, E2F4, RABL6, and CDK4. According to IPA, most of these upstream regulators were inhibited in our FSK treatment. These results revealed that PKA activation may inhibit granulosa cell proliferation, which was previously highlighted by our team [43]. Both cAMP and PKA activation were suggested to stimulate the PKB pathway, which is in line with our findings, where some predicted regulators involved in the PKB pathway, such as p38 mitogen-activated protein kinase (MAPK14) and forkhead box O1 (FOXO1), were identified [98,99]. The FOXO1 protein was inhibited following PKB activation. Crosstalk between PKA and PKB in KGN cells was previously described [43].

**Figure 2:**
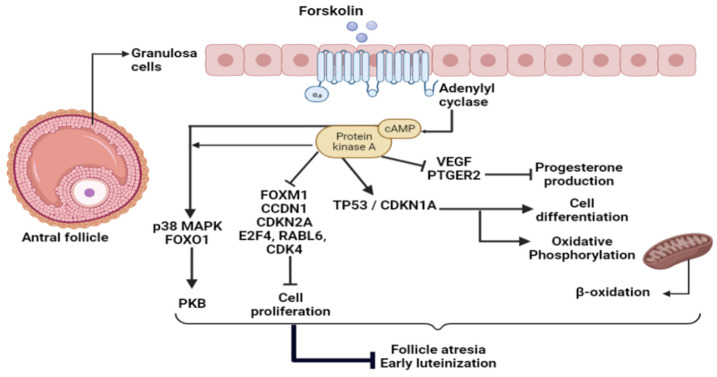
FSK effects on follicular development at the antral stage.

ERBB2 and AREG, two ovulation-related factors, were among the most significant upstream regulators and were inhibited by PKA stimulation. It is known that ERBB2 and AREG stimulate glucose uptake and could regulate mitochondrial functions through switching cancer cell metabolism from OXPHOS to glycolysis to enhance cell growth [24,25,26,27,28,29,30,31,32,33,34,35,36,37,38,39,40,41,42,43,44,45,46,47,48,49,50,51,52,53,54,55,56,57,58,59,60,61,62,63,64,65,66,67,68,69,70,71,72,73,74,75,76,77,78,79,80,81,82,83,84,85,86,87,88,89,90,91,92,93,94,95,96,97,98,99,100]. At the dominant follicular stage, when the follicle enters the plateau phase, PKA activation by FSH was involved in cell proliferation inhibition [43]. This is consistent with our present data, where the yes-associated protein 1 (YAP1) was identified as inhibited by IPA. The YAP1 protein, which is the major effector of Hippo signaling, was identified as a promoter of KGN cell proliferation and a suppressor of cell differentiation. Treatment with FSK induced suppression of YAP1 activity in KGN cells [101], confirming the suggestion that the antral follicle passes from a growing phase to a plateau phase, where cell proliferation slows down in a PKA-dependent manner [102]. Taken together, our results suggest that PKA activation, through cAMP accumulation in response to FSK or other cAMP-inducing hormones, such as LH or prostaglandins, may control ovarian processes during the periovulatory period to improve granulosa cell survival and prevent follicular atresia (Figure 2).

## 4. Materials and Methods

### 4.1. Chemicals

Unless otherwise stated, all chemicals, such as 4-androstene-3,17-dione (product A9630), melatonin (MLT) (product M5250), and forskolin (FSK) (product F3917), were purchased from Sigma-Aldrich (St. Louis, MO, USA). Each was dissolved in 95% ethanol to make stock solutions.

### 4.2. Human Granulosa-like Tumor Cell Line (KGN)

The granulosa cell line KGN was purchased from the RIKEN Bioresource Centre (Tsukuba, Japan). This cell line presents some physiological characteristics of normal human granulosa cells, such as steroidogenic activities and functional FSH receptors [14].

### 4.3. Cell Culture

The KGN cells were cultured as previously described [43]. Briefly, the cells were cultured in DMEM/F12 medium (Life technologies) supplemented with 10% fetal bovine serum (Corning, NY, USA) and ×100 Penicillin-Streptomycin (10,000 U/mL) (Life technologies, Waltham, MA, USA) in an atmosphere of 5% CO_2_/95% O_2_ at 37 °C. The cells were thawed on day 1, placed in a cell culture flask (75 cm^3^, Sigma-Aldrich), and sub-cultured on day 3. Confluence was never over 80%. On day 6, cells were placed in a 6-well dish at a density of 1.510^5^ cells per well in 1.5 mL of medium and grown for 72 h, at which point they were reaching almost full confluency. For all experimental conditions, the medium was replaced with charcoal-stripped (SVFA) DMEM/F12 containing antibiotics and 100 nmol L^−1^ of 4-androstene-3,17-dione. The 24 h treatments consisted of adding either ethanol as a control (less than 0.05% of the final culture volume) or additional stimulators in ethanol to obtain either 1 nmol of melatonin L^−1^ or 1 μmol of FSK L^−1^ as final concentrations. The experiment was repeated four times (four different weeks) to generate four biological replicates for RNAseq analysis.

### 4.4. RNA Purification and Deep Sequencing

Total RNA was isolated with the AllPrep DNA/RNA Mini Kit (Qiagen, Mississauga, ON, Canada) according to the manufacturer’s instructions. Total RNA integrity and concentrations were evaluated on a 2100-Bioanalyzer (Agilent Technologies, Palo Alto, CA, USA). The RNA Integrity Number of all samples was 10. The total RNA input used for sequencing was 360 ng for each treatment, which consisted of 90 ng from each biological replicate. The mRNA was isolated from total RNA using the NEBNext Poly (A) mRNA Magnetic Isolation Module (E7490S; NEB).

PolyA-selected mRNA was fragmented to a mean size of 200 nt, reverse transcribed to generate double-stranded cDNA, and converted to a paired-end library using the NEBNext Ultra RNA Library Prep kit for Illumina (E7530S; NEB) according to the manufacturer’s instructions, with Agencourt AMPure XP beads and NEBNext Mutliplex Oligos for Illumina (set1, E7335S; NEB). Libraries were sent to the Génome Québec Innovation Centre at McGill University for quality control tests. Libraries were then pooled at equimolar concentrations and sequenced on an Illumina HiSeq4000 in paired-end mode with 100 base pair reads (PE100) to a depth between 55 and 67 million reads.

### 4.5. Transcriptome Assembly and Expression Level Estimate from Reading Counts

Ensembl (release 91) was used as the source of annotated genes and transcript isoforms. Trimming of adapters was performed using Trimmomatic; sequencing adapters were removed, and base calls with a quality score below 30 were removed from the end of the reads [103]. Trimming was performed with the minimal length set at 32 nt, and these reads were kept for further processing. Pseudoalignment of all transcripts described in release 91 of ENSEMBL cDNA gene annotation was accomplished by the kallisto tool [104]. Differential expression of genes was then assessed using pairwise comparisons in EdgeR [105]. Because of the lack of replicates, dispersion within EdgeR was evaluated by grouping samples for similar comparisons (first for control, then for melatonin (MLT) and FSK samples) and dropping the factor with the lowest impact as suggested by the EdgeR manual. According to the Transcript Support Level (TSL) used by the Ensembl gene annotation system, normalized data from RNAseq analysis should be first sorted for significance and then filtered to highlight the well-supported and poorly supported transcript models that rely on the comparison of the mRNA and EST alignments to the GENCODE transcripts, and the transcripts are scored according to how well the alignment matches over its full length. Differentially expressed genes selected in this present analysis were assigned to the evaluated annotation tsl1, which means that all splice junctions of the transcript were supported by at least one non-suspect mRNA.

### 4.6. Ingenuity Pathway Analysis

Functional analysis was applied using the ingenuity pathway analysis software (IPA), a web-based software that enables the interpretation of data derived from omics experiments, to create a global picture of the principal upstream regulators and genomic mechanisms implicated in the treatments. This software can compare our dataset to the ingenuity knowledge Base to provide biological interactions and functional annotations and attribute a probability of association between genes in our data and biological functions. We uploaded, separately, the lists of differential expressed genes from RNA-seq analysis of each treatment into the IPA software to be analyzed. Then, we extracted the major biological functions as well as the most significant upstream regulators to our dataset.

## 5. Conclusions

The results of this study suggest that melatonin supplementation in a dominant-like plateau phase follicular context may activate PKB/mTOR signaling to program granulosa cell metabolism during the switch in the hormone-dependent state and following the FSH decline. These metabolic effects include balancing between glycolysis and oxidative phosphorylation and between mitochondrial fission and fusion to support slower growth and proliferation of granulosa cells as well as balancing between follicular atresia and cell survival and regulating granulosa cell early differentiation. Protein kinaseA activation by FSK stimulation of cAMP synthesis exerted similar effects as melatonin. Additionally, the PKA and cAMP-dependent activation of PKB suggests that melatonin may act through PKA and PKB simultaneously in human granulosa cells via its membrane receptors and its free radical scavenging activity to prevent follicular atresia or luteinization at the antral stage. These findings confirmed previous reports suggesting that melatonin, acting through the PKB/mTOR signaling pathway, plays an essential role in follicular somatic cell metabolic programming to improve oocyte developmental competence. To the best of our knowledge, this is the first study to investigate the signaling pathways involved in the “metabolic programmer” role of melatonin in human granulosa cells. Given the crosstalk between PKA and PKB signaling in ovarian granulosa cells, this study supports these findings and suggests that melatonin may act through both PKB/mTOR and PKA pathways to prevent follicular atresia and delay ovarian aging. We also showed that the KGN cell line is a unique and useful in vitro model to study the transcriptomics and metabolomics of follicular dominance/growth and oocyte competence acquisition processes. Future studies focused on the effects of specific activators and inhibitors of the melatonin signaling pathways and metabolic activity are required to demystify further the intricate PKB signaling involved in granulosa cell metabolism and antral follicle growth.

## Figures and Tables

**Table 1:** Significant upstream regulators were identified following IPA upstream analysis of differential gene expression in KGN granulosa cells treated with melatonin (*p* < 0.05).

Upstream Regulator	Predicted Activation State	Activation z-Score	*p*-Value of Overlap
DAP3	Inhibited	−2.449	2.64 × 10^−6^
MT-TE			4.96 × 10^−5^
LONP1		−0.522	7.91 × 10^−5^
STOX1			8.31 × 10^−5^
Actinonin	Activated	2.236	2.35 × 10^−4^
HIF1A		0.520	6.20 × 10^−4^
MALSU1			8.09 × 10^−4^
AP2α			8.09 × 10^−4^
MRPL14			1.38 × 10^−3^
SIRT3	Activated	2.020	1.99 × 10^−3^

**Table 2:** Significant upstream regulators were identified following IPA upstream analysis of differential gene expression in KGN granulosa cells treated with FSK (*p* < 0.05).

Upstream Regulator	Predicted Activation State	Activation z-Score	*p*-Value of Overlap
ZBTB17			1.55 × 10^−26^
Vegf	Inhibited	−2.090	5.96 × 10^−18^
PTGER2	Inhibited	−3.394	1.09 × 10^−15^
TP53		1.729	5.11 × 10^−15^
HGF	Inhibited	−2.072	7.72 × 10^−15^
CDKN1A		1.907	8.50 × 10^−15^
FOXM1	Inhibited	−3.168	8.99 × 10^−14^
NR1H3			1.10 × 10^−13^
AREG	Inhibited	−3.357	3.02 × 10^−13^

**Table 3:** Canonical pathways affected by the treatments and related upstream regulators identified by IPA.

Canonical Pathways	Upstream Regulators	z-Score
Melatonin		
Oxidative Phosphorylation	ATP5PF, COX11, COX4I1, MT-ATP6, MT-CO3, MT-CYB, MT-ND1, MT-ND4, NDUFA2, NDUFA6, NDUFB8, NDUFV3, UQCRFS1	−1.941
Mitochondrial Dysfunction	ATP5PF, COX11, COX4I1, FIS1, HSD17B10, MT-ATP6, MT-CO3, MT-CYB, MT-ND1, MT-ND4, NDUFA2, NDUFA6, NDUFB8, NDUFV3, PDHA1, UQCRFS1	
Sirtuin Signaling Pathway	ATG14, ATG16L1, ATP5PF, CPS1, H3F3A/H3F3B, LDHD, MT-ATP6, MT-CYB, MT-ND1, MT-ND4, NDUFA2, NDUFA6, NDUFB8, NDUFV3, PDHA1, POLR1E, PPARA, TIMM13, TOMM40L, UQCRFS1	1.941
TGF-β Signaling	ACVR1C, MAP3K7, MAPK11, PIAS4, RALB, RUNX2, TGFB2, TGIF1	1.134
Thiamin Salvage III	TPK1	
FSK		
tRNA Splicing	PDE1A, PDE3A, PDE4B, PDE4D, PDE7B	2.236
Protein Kinase A Signaling	ADCY1, CDKN3, DUSP5, GNB3, LEF1, MYLK2, PDE1A, PDE3A, PDE4B, PDE4D, PDE7B, PLCL1, PTPRN, PTPRR	1.732
Glutamate Receptor Signaling	GNB3, GRIA4, SLC1A3, SLC1A7	
TGF-β Signaling	BCL2, INHA, PITX2, RASD1, VDR	
Methylglyoxal Degradation VI	LDHD	

## Data Availability

The data discussed in this publication have been deposited in NCBI’s Gene Expression Omnibus (GEO) and are accessible through GEO Series accession number GSE189124. https://www.ncbi.nlm.nih.gov/geo/query/acc.cgi?acc=GSE189124 (accessed on 16 January 2021).

## Supplementary Materials

The following are available online at https://www.mdpi.com/article/10.3390/ijms23062988/s1.
