# Supplementary material for: Melatonin Signaling Pathways Implicated in Metabolic Processes in Human Granulosa Cells (KGN)
Source: Int J Mol Sci. 2022 Mar 10;23(6):2988. doi: 10.3390/ijms23062988 (PMC8950389; doi:10.3390/ijms23062988)
Supplement: Supplementary file 1 [file ijms-23-02988-s001.zip › ijms-1608887-supplementary.pdf]

Table S1. List of DEGs in melatonin treatment (FC = 0, adjusted *p*-value ≤ 0.05).

Symbol	*Entrez Gene Name*	*ENSEMBL*	*Expr Log Ratio*	*Expr p-Value*
AMOTL2	angiomotin like 2	ENSG00000114019	0.277	1.00E-02
AP4S1	adaptor related protein complex 4 subunit sigma 1	ENSG00000100478	-0.711	1.00E-02
HELQ	helicase, POLQ like	ENSG00000163312	0.739	1.00E-02
ORC2	origin recognition complex subunit 2	ENSG00000115942	-0.427	1.00E-02
PREX1	phosphatidylinositol-3,4,5-trisphosphate dependent Rac exchange factor 1	ENSG00000124126	-0.636	1.00E-02
TIMM13	translocase of inner mitochondrial membrane 13	ENSG00000099800	-0.284	1.00E-02
TOMM40L	translocase of outer mitochondrial membrane 40 like	ENSG00000158882	0.626	1.00E-02
TRMT112	tRNA methyltransferase subunit 11-2	ENSG00000173113	-0.244	1.00E-02
ZNF707	zinc finger protein 707	ENSG00000181135	-0.590	1.00E-02
OXLD1	oxidoreductase like domain containing 1	ENSG00000204237	0.597	1.00E-03
THOC1	THO complex 1	ENSG00000079134	-0.633	1.00E-03
AE00065822		ENSG00000257096	-1.515	1.10E-02
C4orf36	chromosome 4 open reading frame 36	ENSG00000163633	-1.918	1.10E-02
COL8A2	collagen type VIII alpha 2 chain	ENSG00000171812	-1.918	1.10E-02
COLQ	collagen like tail subunit of asymmetric acetylcholinesterase	ENSG00000206561	-1.189	1.10E-02
FHL3	four and a half LIM domains 3	ENSG00000183386	0.459	1.10E-02
GAS1	growth arrest specific 1	ENSG00000180447	-1.276	1.10E-02
KMT5C	lysine methyltransferase 5C	ENSG00000133247	0.581	1.10E-02
LINC00894	long intergenic non-protein coding RNA 894	ENSG00000235703	1.837	1.10E-02
LOC101928069	uncharacterized LOC101928069	ENSG00000255517	0.906	1.10E-02
NEK11	NIMA related kinase 11	ENSG00000114670	-0.730	1.10E-02
PCCA-DT	PCCA divergent transcript	ENSG00000274605	1.625	1.10E-02
PLAUR	plasminogen activator, urokinase receptor	ENSG00000011422	-0.332	1.10E-02
RIPOR1	RHO family interacting cell polarization regulator 1	ENSG00000039523	0.541	1.10E-02
RP9P	RP9 pseudogene	ENSG00000205763	-0.646	1.10E-02
SEC23B	SEC23 homolog B, coat complex II component	ENSG00000101310	0.325	1.10E-02
SLC27A3	solute carrier family 27 member 3	ENSG00000143554	-1.189	1.10E-02
SLFN12L	schlafen family member 12 like	ENSG00000205045	1.837	1.10E-02
TDRD7	tudor domain containing 7	ENSG00000196116	-0.505	1.10E-02
ZNF674	zinc finger protein 674	ENSG00000251192	1.311	1.10E-02
AC0059436		ENSG00000279009	0.428	1.20E-02
CHCHD10	coiled-coil-helix-coiled-coil-helix domain containing 10	ENSG00000250479	-2.612	1.20E-02
CSF1	colony stimulating factor 1	ENSG00000184371	-0.417	1.20E-02
CTSL	cathepsin L	ENSG00000135047	-0.222	1.20E-02
DCTN3	dynactin subunit 3	ENSG00000137100	-0.315	1.20E-02
ELMO3	engulfment and cell motility 3	ENSG00000102890	1.025	1.20E-02
ELP2	elongator acetyltransferase complex subunit 2	ENSG00000134759	-0.319	1.20E-02
FAM111A-DT	FAM111A divergent transcript	ENSG00000245571	-0.915	1.20E-02

ITGA10	integrin subunit alpha 10	ENSG00000143127	1.414	1.20E-02
JAK2	Janus kinase 2	ENSG00000096968	0.438	1.20E-02
KANK1	KN motif and ankyrin repeat domains 1	ENSG00000107104	-0.391	1.20E-02
LIMD1-AS1	LIMD1 antisense RNA 1	ENSG00000230530	0.810	1.20E-02
LOC101926943	uncharacterized LOC101926943	ENSG00000232729	0.428	1.20E-02
NANOS3	nanos C2HC-type zinc finger 3	ENSG00000187556	-2.612	1.20E-02
NPY1R	neuropeptide Y receptor Y1	ENSG00000164128	-0.437	1.20E-02
PALM2AKAP2	PALM2 and AKAP2 fusion	ENSG00000157654	-0.766	1.20E-02
PCLAF	PCNA clamp associated factor	ENSG00000166803	0.523	1.20E-02
RP11_2B62		ENSG00000269929	-2.612	1.20E-02
RP11_315D164		ENSG00000260657	0.934	1.20E-02
RP11_399B171		ENSG00000278962	-1.983	1.20E-02
RP11_603J245		ENSG00000258317	0.432	1.20E-02
RP11_63M222		ENSG00000260465	0.392	1.20E-02
RPS26	ribosomal protein S26	ENSG00000197728	-0.227	1.20E-02
SLC37A4	solute carrier family 37 member 4	ENSG00000137700	0.458	1.20E-02
SSSCA1-AS1	SSSCA1 antisense RNA 1 (head to head)	ENSG00000260233	2.097	1.20E-02
TMEM80	transmembrane protein 80	ENSG00000177042	-0.478	1.20E-02
ZFP3	ZFP3 zinc finger protein	ENSG00000180787	-0.766	1.20E-02
ZFPM1	zinc finger protein, FOG family member 1	ENSG00000179588	0.381	1.20E-02
COX4I1	cytochrome c oxidase subunit 4I1	ENSG00000131143	-0.290	1.20E-03
TPST2	tyrosylprotein sulfotransferase 2	ENSG00000128294	-0.478	1.20E-03
ZC3H8	zinc finger CCCH-type containing 8	ENSG00000144161	-1.069	1.20E-03
ADSS1	adenylosuccinate synthase 1	ENSG00000185100	3.153	1.30E-02
GLYR1	glyoxylate reductase 1 homolog	ENSG00000140632	0.267	1.30E-02
KIAA0930	KIAA0930	ENSG00000100364	0.328	1.30E-02
METTL25	methyltransferase like 25	ENSG00000127720	1.252	1.30E-02
MIR181A1HG	MIR181A1 host gene	ENSG00000229989	-2.073	1.30E-02
MPV17	mitochondrial inner membrane protein MPV17	ENSG00000115204	-0.326	1.30E-02
PRPF40B	pre-mRNA processing factor 40 homolog B	ENSG00000110844	0.312	1.30E-02
RNF208	ring finger protein 208	ENSG00000212864	-1.513	1.30E-02
RP11-753C18.12		ENSG00000282772	1.023	1.30E-02
RP11_120K92		ENSG00000259684	3.153	1.30E-02
RP11_27M241		ENSG00000256013	-0.753	1.30E-02
RP11_459E51		ENSG00000253125	-0.904	1.30E-02
TUBE1	tubulin epsilon 1	ENSG00000074935	0.782	1.30E-02
USP12	ubiquitin specific peptidase 12	ENSG00000152484	0.398	1.30E-02
ZBED1	zinc finger BED-type containing 1	ENSG00000214717	-0.277	1.30E-02
LIME1	Lck interacting transmembrane adaptor 1	ENSG00000203896	1.408	1.30E-03
AC0936735		ENSG00000232533	-0.655	1.40E-02
ARL4A	ADP ribosylation factor like GTPase 4A	ENSG00000122644	-0.324	1.40E-02
BRCA2	BRCA2 DNA repair associated	ENSG00000139618	0.554	1.40E-02
GLI4	GLI family zinc finger 4	ENSG00000250571	0.547	1.40E-02
LINC00511	long intergenic non-protein coding RNA 511	ENSG00000227036	-1.325	1.40E-02
MARCHF7	membrane associated ring-CH-type finger 7	ENSG00000136536	0.258	1.40E-02
NKILA		ENSG00000278709	-1.449	1.40E-02
OAS3	2'-5'-oligoadenylate synthetase 3	ENSG00000111331	0.351	1.40E-02
RLF	rearranged L-myc fusion	ENSG00000117000	0.431	1.40E-02
SLC9A3-AS1	SLC9A3 antisense RNA 1	ENSG00000225138	0.286	1.40E-02
SMYD5	SMYD family member 5	ENSG00000135632	0.427	1.40E-02
SUGT1	SGT1 homolog, MIS12 kinetochore complex	ENSG00000165416	-0.296	1.40E-02

	assembly cochaperone			
ZFP69B	ZFP69 zinc finger protein B	ENSG00000187801	0.892	1.40E-02
RNF215	ring finger protein 215	ENSG00000099999	-0.641	1.40E-03
ZBTB11	zinc finger and BTB domain containing 11	ENSG00000066422	0.492	1.40E-03
B3GNT7	UDP-GlcNAc:betaGal beta-1,3-N-acetylglucosaminyltransferase 7	ENSG00000156966	-1.511	1.50E-02
CIRBP-AS1	CIRBP antisense RNA 1	ENSG00000267493	-1.221	1.50E-02
DIS3L2	DIS3 like 3'-5' exoribonuclease 2	ENSG00000144535	0.426	1.50E-02
EEPD1	endonuclease/exonuclease/phosphatase family domain containing 1	ENSG00000122547	1.270	1.50E-02
GALM	galactose mutarotase	ENSG00000143891	-0.543	1.50E-02
MAPT	microtubule associated protein tau	ENSG00000186868	-1.221	1.50E-02
NAA20	N(alpha)-acetyltransferase 20, NatB catalytic subunit	ENSG00000173418	-0.318	1.50E-02
PIGP	phosphatidylinositol glycan anchor biosynthesis class P	ENSG00000185808	0.791	1.50E-02
PPP4R3B	protein phosphatase 4 regulatory subunit 3B	ENSG00000275052	0.289	1.50E-02
PSMF1	proteasome inhibitor subunit 1	ENSG00000125818	0.274	1.50E-02
RADX	RPA1 related single stranded DNA binding protein, X-linked	ENSG00000147231	1.165	1.50E-02
RP11_1055B84		ENSG00000262877	-0.833	1.50E-02
STX4	syntaxin 4	ENSG00000103496	-0.297	1.50E-02
SYDE1	synapse defective Rho GTPase homolog 1	ENSG00000105137	0.260	1.50E-02
TMEM116	transmembrane protein 116	ENSG00000198270	0.927	1.50E-02
UTP15	UTP15 small subunit processome component	ENSG00000164338	-0.493	1.50E-02
SUSD6	sushi domain containing 6	ENSG00000100647	-0.497	1.50E-03
SLX4IP	SLX4 interacting protein	ENSG00000149346	1.258	1.50E-04
ALDH3B1	aldehyde dehydrogenase 3 family member B1	ENSG00000006534	0.336	1.60E-02
APBA1	amyloid beta precursor protein binding family A member 1	ENSG00000107282	0.812	1.60E-02
APOBEC3G	apolipoprotein B mRNA editing enzyme catalytic subunit 3G	ENSG00000239713	-0.883	1.60E-02
APOL1	apolipoprotein L1	ENSG00000100342	-0.417	1.60E-02
B4GAT1	beta-1,4-glucuronyltransferase 1	ENSG00000174684	0.318	1.60E-02
JCAD	junctional cadherin 5 associated	ENSG00000165757	0.231	1.60E-02
KLHDC8B	kelch domain containing 8B	ENSG00000185909	0.496	1.60E-02
RRP1	ribosomal RNA processing 1	ENSG00000160214	-0.344	1.60E-02
TMEM79	transmembrane protein 79	ENSG00000163472	0.424	1.60E-02
TPK1	thiamin pyrophosphokinase 1	ENSG00000196511	-0.773	1.60E-02
TTI2	TELO2 interacting protein 2	ENSG00000129696	-0.432	1.60E-02
ZNF691	zinc finger protein 691	ENSG00000164011	-0.789	1.60E-02
ST7L	suppression of tumorigenicity 7 like	ENSG00000007341	-0.649	1.60E-03
MT-ATP6	ATP synthase F0 subunit 6	ENSG00000198899	-0.292	1.60E-04
ACAD8	acyl-CoA dehydrogenase family member 8	ENSG00000151498	0.415	1.70E-02
AP00306812		ENSG00000255173	-0.561	1.70E-02
ARMC1	armadillo repeat containing 1	ENSG00000104442	-0.330	1.70E-02
BAIAP2-DT	BAIAP2 divergent transcript	ENSG00000226137	-0.330	1.70E-02
CHL1	cell adhesion molecule L1 like	ENSG00000134121	0.415	1.70E-02
COG2	component of oligomeric golgi complex 2	ENSG00000135775	-0.543	1.70E-02
CTB_12A173		ENSG00000269292	-0.308	1.70E-02

FARSA	phenylalanyl-tRNA synthetase subunit alpha	ENSG00000179115	-0.254	1.70E-02
FLCN	folliculin	ENSG00000154803	-0.361	1.70E-02
GIT1	GIT ArfGAP 1	ENSG00000108262	-0.272	1.70E-02
LINC01852		ENSG00000236914	-1.441	1.70E-02
MANEA	mannosidase endo-alpha	ENSG00000172469	0.580	1.70E-02
PLA2G4A	phospholipase A2 group IVA	ENSG00000116711	0.540	1.70E-02
RP11_264B174		ENSG00000260367	0.540	1.70E-02
RPS27	ribosomal protein S27	ENSG00000177954	-0.204	1.70E-02
SNX18	sorting nexin 18	ENSG00000178996	0.218	1.70E-02
SYNPO2	synaptopodin 2	ENSG00000172403	0.231	1.70E-02
TCTN2	tectonic family member 2	ENSG00000168778	-0.503	1.70E-02
ITPKB	inositol-trisphosphate 3-kinase B	ENSG00000143772	0.591	1.70E-03
ZNF843	zinc finger protein 843	ENSG00000176723	-2.005	1.70E-03
ACSS1	acyl-CoA synthetase short chain family member 1	ENSG00000154930	1.318	1.80E-02
ALG1L9P	ALG1 like 9, pseudogene	ENSG00000248671	-1.608	1.80E-02
BHLHE40	basic helix-loop-helix family member e40	ENSG00000134107	-0.245	1.80E-02
FAM222A	family with sequence similarity 222 member A	ENSG00000139438	1.214	1.80E-02
FANCB	FA complementation group B	ENSG00000181544	0.948	1.80E-02
FNBP1	formin binding protein 1	ENSG00000187239	-0.286	1.80E-02
GPN2	GPN-loop GTPase 2	ENSG00000142751	-0.383	1.80E-02
KCNAB3	potassium voltage-gated channel subfamily A regulatory beta subunit 3	ENSG00000170049	1.318	1.80E-02
KCNIP2	potassium voltage-gated channel interacting protein 2	ENSG00000120049	1.318	1.80E-02
KCNK3	potassium two pore domain channel subfamily K member 3	ENSG00000171303	0.410	1.80E-02
KIF15	kinesin family member 15	ENSG00000163808	0.428	1.80E-02
NEIL3	nei like DNA glycosylase 3	ENSG00000109674	0.716	1.80E-02
OTUB1	OTU deubiquitinase, ubiquitin aldehyde binding 1	ENSG00000167770	0.244	1.80E-02
RP11_229E134		ENSG00000274630	0.428	1.80E-02
RP11_490E152		ENSG00000233021	1.214	1.80E-02
SPEF2	sperm flagellar 2	ENSG00000152582	1.768	1.80E-02
STAG3L5P-PVRIG2P-PILRB		ENSG00000272752	-1.608	1.80E-02
TBRG1	transforming growth factor beta regulator 1	ENSG00000154144	-0.331	1.80E-02
TRIM66	tripartite motif containing 66	ENSG00000166436	-0.561	1.80E-02
GLYCTK	glycerate kinase	ENSG00000168237	-1.028	1.80E-03
CPS1	carbamoyl-phosphate synthase 1	ENSG00000021826	0.382	1.90E-02
IFITM1	interferon induced transmembrane protein 1	ENSG00000185885	-0.645	1.90E-02
MAPK11	mitogen-activated protein kinase 11	ENSG00000185386	0.823	1.90E-02
MIR1915HG	MIR1915 host gene	ENSG00000204682	-0.731	1.90E-02
NDUFC1	NADH:ubiquinone oxidoreductase subunit C1	ENSG00000109390	-0.416	1.90E-02
PIGBOS1	PIGB opposite strand 1	ENSG00000225973	-0.731	1.90E-02
PLA2G15	phospholipase A2 group XV	ENSG00000103066	0.401	1.90E-02
PPP4R3A	protein phosphatase 4 regulatory subunit 3A	ENSG00000100796	0.278	1.90E-02
SLC35F5	solute carrier family 35 member F5	ENSG00000115084	0.333	1.90E-02

TEAD2	TEA domain transcription factor 2	ENSG00000074219	-0.645	1.90E-02
TMEM60	transmembrane protein 60	ENSG00000135211	-0.731	1.90E-02
VGLL4	vestigial like family member 4	ENSG00000144560	-0.225	1.90E-02
ZNFX1	zinc finger NFX1-type containing 1	ENSG00000124201	0.258	1.90E-02
PSMD12	proteasome 26S subunit, non-ATPase 12	ENSG00000197170	0.404	1.90E-03
SLC25A32	solute carrier family 25 member 32	ENSG00000164933	0.569	1.90E-03
AP0016105		ENSG00000228318	2.020	2.00E-02
C3	complement C3	ENSG00000125730	-1.431	2.00E-02
COA8	cytochrome c oxidase assembly factor 8	ENSG00000256053	0.471	2.00E-02
DLX5	distal-less homeobox 5	ENSG00000105880	-1.030	2.00E-02
DSTNP2	DSTN pseudogene 2	ENSG00000248593	1.653	2.00E-02
DUSP4	dual specificity phosphatase 4	ENSG00000120875	-0.219	2.00E-02
EZH1	enhancer of zeste 1 polycomb repressive complex 2 subunit	ENSG00000108799	0.414	2.00E-02
G3BP1	G3BP stress granule assembly factor 1	ENSG00000145907	0.218	2.00E-02
INE1	inactivation escape 1	ENSG00000224975	1.653	2.00E-02
KAT8	lysine acetyltransferase 8	ENSG00000103510	-0.339	2.00E-02
MBTD1	mbt domain containing 1	ENSG00000011258	-0.456	2.00E-02
ORC6	origin recognition complex subunit 6	ENSG00000091651	-0.417	2.00E-02
PTPN4	protein tyrosine phosphatase non-receptor type 4	ENSG00000088179	0.471	2.00E-02
RP11_860B131		ENSG00000256747	0.588	2.00E-02
RP11_867G234		ENSG00000254452	-0.280	2.00E-02
SELENOM	selenoprotein M	ENSG00000198832	0.399	2.00E-02
SIVA1	SIVA1 apoptosis inducing factor	ENSG00000184990	0.318	2.00E-02
SLC35A5	solute carrier family 35 member A5	ENSG00000138459	0.308	2.00E-02
ST3GAL4	ST3 beta-galactoside alpha-2,3-sialyltransferase 4	ENSG00000110080	-0.293	2.00E-02
TOR2A	torsin family 2 member A	ENSG00000160404	0.709	2.00E-02
AC0056143		ENSG00000269296	6.362	2.00E-03
PIGH	phosphatidylinositol glycan anchor biosynthesis class H	ENSG00000100564	0.840	2.00E-03
RP11_400K93		ENSG00000254204	6.238	2.00E-03
RP11_507J182		ENSG00000259773	-1.090	2.00E-04
ARFGEF3	ARFGEF family member 3	ENSG00000112379	-0.925	2.10E-02
ATP6V0E1	ATPase H+ transporting V0 subunit e1	ENSG00000113732	-0.234	2.10E-02
CBX8	chromobox 8	ENSG00000141570	-0.641	2.10E-02
CORO2A	coronin 2A	ENSG00000106789	0.990	2.10E-02
DHFR2	dihydrofolate reductase 2	ENSG00000178700	-0.782	2.10E-02
HLX-AS1	HLX antisense RNA 1	ENSG00000257551	-1.255	2.10E-02
IGFBP1	insulin like growth factor binding protein 1	ENSG00000146678	-0.782	2.10E-02
ILVBL	ilvB acetolactate synthase like	ENSG00000105135	-0.317	2.10E-02
KCTD7	potassium channel tetramerization domain containing 7	ENSG00000243335	-0.612	2.10E-02
KRT81	keratin 81	ENSG00000205426	-0.848	2.10E-02
LOC440434	aminopeptidase puromycin sensitive pseudogene	ENSG00000274487	-1.379	2.10E-02
NDUFA6	NADH:ubiquinone oxidoreductase subunit A6	ENSG00000184983	-0.359	2.10E-02
PIGW	phosphatidylinositol glycan anchor biosynthesis class W	ENSG00000277161	0.568	2.10E-02
POLR1E	RNA polymerase I subunit E	ENSG00000137054	-0.414	2.10E-02
PRPF38A	pre-mRNA processing factor 38A	ENSG00000134748	-0.330	2.10E-02

RCN1P2	reticulocalbin 1 pseudogene 2	ENSG00000214455	-0.513	2.10E-02
RP11_527N222		ENSG00000253746	0.857	2.10E-02
S100A6	S100 calcium binding protein A6	ENSG00000197956	-0.181	2.10E-02
SACM1L	SAC1 like phosphatidylinositide phosphatase	ENSG00000211456	-0.332	2.10E-02
THOC3	THO complex 3	ENSG00000051596	-0.403	2.10E-02
UBASH3B	ubiquitin associated and SH3 domain containing B	ENSG00000154127	0.405	2.10E-02
XRCC1	X-ray repair cross complementing 1	ENSG00000073050	-0.296	2.10E-02
ZNF506	zinc finger protein 506	ENSG00000081665	-0.697	2.10E-02
APOOL	apolipoprotein O like	ENSG00000155008	0.480	2.20E-02
C3orf52	chromosome 3 open reading frame 52	ENSG00000114529	1.199	2.20E-02
C8G	complement C8 gamma chain	ENSG00000176919	-2.444	2.20E-02
CDKN1C	cyclin dependent kinase inhibitor 1C	ENSG00000129757	-2.444	2.20E-02
CTD_2026D202		ENSG00000253347	-2.444	2.20E-02
GMPR	guanosine monophosphate reductase	ENSG00000137198	-0.513	2.20E-02
GRTP1	growth hormone regulated TBC protein 1	ENSG00000139835	-1.949	2.20E-02
GS1_293C51		ENSG00000273138	-0.586	2.20E-02
HASPIN	histone H3 associated protein kinase	ENSG00000177602	0.554	2.20E-02
ITGB7	integrin subunit beta 7	ENSG00000139626	2.674	2.20E-02
KCNB1	potassium voltage-gated channel subfamily B member 1	ENSG00000158445	2.674	2.20E-02
LINC02298	long intergenic non-protein coding RNA 2298	ENSG00000257556	2.674	2.20E-02
LOC105375787	uncharacterized LOC105375787	ENSG00000254019	2.674	2.20E-02
NIPAL1	NIPA like domain containing 1	ENSG00000163293	-1.018	2.20E-02
PCF11	PCF11 cleavage and polyadenylation factor subunit	ENSG00000165494	-0.340	2.20E-02
RIN1	Ras and Rab interactor 1	ENSG00000174791	-0.376	2.20E-02
RNF227	ring finger protein 227	ENSG00000179859	-1.172	2.20E-02
RP11_263K196		ENSG00000236263	-2.444	2.20E-02
RP11_344N105		ENSG00000272630	2.674	2.20E-02
RP11_432J222		ENSG00000238058	2.674	2.20E-02
RP11_467L137		ENSG00000276900	-2.444	2.20E-02
RP11_531A245		ENSG00000253636	-2.444	2.20E-02
RP11_689P113		ENSG00000251186	2.674	2.20E-02
RPL10AP6	ribosomal protein L10a pseudogene 6	ENSG00000226360	-1.949	2.20E-02
SEPTIN9-DT		ENSG00000263718	2.674	2.20E-02
SLC22A20P	solute carrier family 22 member 20, pseudogene	ENSG00000197847	2.674	2.20E-02
SSBP3-AS1	SSBP3 antisense RNA 1	ENSG00000198711	-2.444	2.20E-02
TIGD3	tigger transposable element derived 3	ENSG00000173825	2.674	2.20E-02
WI2-87327B8.2		ENSG00000273812	-2.444	2.20E-02
ZWINT	ZW10 interacting kinetochore protein	ENSG00000122952	-0.252	2.20E-02
C22orf46	CTA-216E10.6	ENSG00000184208	-0.556	2.20E-03
CDC14A	cell division cycle 14A	ENSG00000079335	1.433	2.20E-03
GPAT3	glycerol-3-phosphate acyltransferase 3	ENSG00000138678	-1.519	2.20E-03
TRIM52-AS1	TRIM52 antisense RNA 1 (head to head)	ENSG00000248275	-1.080	2.20E-03
USP36	ubiquitin specific peptidase 36	ENSG00000055483	-0.395	2.20E-03
ZFYVE27	zinc finger FYVE-type containing 27	ENSG00000155256	-0.441	2.20E-03
ZNF185	zinc finger protein 185 with LIM domain	ENSG00000147394	-1.080	2.20E-03
PDHA1	pyruvate dehydrogenase E1 alpha 1 subunit	ENSG00000131828	-0.461	2.20E-04

AC0107619		ENSG00000265474	-0.839	2.30E-02
ADPRHL2	ADP-ribosylhydrolase like 2	ENSG00000116863	-0.324	2.30E-02
APOA1	apolipoprotein A1	ENSG00000118137	-2.211	2.30E-02
ATPSCKMT	ATP synthase c subunit lysine N-methyltransferase	ENSG00000150756	0.590	2.30E-02
FAM118A	family with sequence similarity 118 member A	ENSG00000100376	0.538	2.30E-02
FOXRED1	FAD dependent oxidoreductase domain containing 1	ENSG00000110074	-0.378	2.30E-02
LARP6	La ribonucleoprotein domain family member 6	ENSG00000166173	-0.270	2.30E-02
LIPT2	lipoyl(octanoyl) transferase 2	ENSG00000175536	-0.912	2.30E-02
LOC102724684	uncharacterized LOC102724684	ENSG00000267026	-2.211	2.30E-02
MT-ND1	NADH dehydrogenase, subunit 1 (complex I)	ENSG00000198888	-0.187	2.30E-02
MTHFR	methylenetetrahydrofolate reductase	ENSG00000177000	0.397	2.30E-02
NCEH1	neutral cholesterol ester hydrolase 1	ENSG00000144959	-0.357	2.30E-02
OBI1	ORC ubiquitin ligase 1	ENSG00000152193	0.538	2.30E-02
POPDC3	popeye domain containing 3	ENSG00000132429	-1.009	2.30E-02
RPGR	retinitis pigmentosa GTPase regulator	ENSG00000156313	-0.397	2.30E-02
TMEM68	transmembrane protein 68	ENSG00000167904	-0.564	2.30E-02
WASHC5-AS1		ENSG00000253167	-0.776	2.30E-02
SLC44A3-AS1	SLC44A3 antisense RNA 1	ENSG00000224081	1.237	2.30E-03
ZNF528-AS1	ZNF528 antisense RNA 1	ENSG00000269834	1.212	2.30E-03
ABHD10	abhydrolase domain containing 10	ENSG00000144827	-0.410	2.40E-02
B4GALT7	beta-1,4-galactosyltransferase 7	ENSG00000027847	0.367	2.40E-02
CINP	cyclin dependent kinase 2 interacting protein	ENSG00000100865	0.374	2.40E-02
DANCR	differentiation antagonizing non-protein coding RNA	ENSG00000226950	-0.352	2.40E-02
DYNC1LI2	dynein cytoplasmic 1 light intermediate chain 2	ENSG00000135720	-0.216	2.40E-02
LINC00863	long intergenic non-protein coding RNA 863	ENSG00000224914	-0.564	2.40E-02
PDLIM4	PDZ and LIM domain 4	ENSG00000131435	-0.406	2.40E-02
RCBTB2	RCC1 and BTB domain containing protein 2	ENSG00000136161	0.575	2.40E-02
RING1	ring finger protein 1	ENSG00000204227	-0.261	2.40E-02
TACC2	transforming acidic coiled-coil containing protein 2	ENSG00000138162	-0.382	2.40E-02
ZNF180	zinc finger protein 180	ENSG00000167384	-0.588	2.40E-02
AC0926875		ENSG00000234818	0.999	2.40E-03
L340794		ENSG00000269583	1.053	2.40E-03
ANKS3	ankyrin repeat and sterile alpha motif domain containing 3	ENSG00000168096	0.401	2.50E-02
ATG16L1	autophagy related 16 like 1	ENSG00000085978	-0.395	2.50E-02
BDKRB2	bradykinin receptor B2	ENSG00000168398	-1.364	2.50E-02
CAMK2D	calcium/calmodulin dependent protein kinase II delta	ENSG00000145349	0.378	2.50E-02
CARNS1	carnosine synthase 1	ENSG00000172508	0.874	2.50E-02
CEP295NL	CEP295 N-terminal like	ENSG00000178404	1.514	2.50E-02
COQ5	coenzyme Q5, methyltransferase	ENSG00000110871	-0.343	2.50E-02
EIF5B	eukaryotic translation initiation factor 5B	ENSG00000158417	-0.231	2.50E-02
FIS1	fission, mitochondrial 1	ENSG00000214253	-0.305	2.50E-02

GABBR2	gamma-aminobutyric acid type B receptor subunit 2	ENSG00000136928	-0.819	2.50E-02
GFER	growth factor, augmenter of liver regeneration	ENSG00000127554	-0.329	2.50E-02
LOC101929240	U5 small nuclear ribonucleoprotein 200 kDa helicase pseudogene	ENSG00000265298	1.019	2.50E-02
LOC339803	uncharacterized LOC339803	ENSG00000212978	0.640	2.50E-02
MLST8	MTOR associated protein, LST8 homolog	ENSG00000167965	-0.302	2.50E-02
MRPS31	mitochondrial ribosomal protein S31	ENSG00000102738	-0.512	2.50E-02
NAGLU	N-acetyl-alpha-glucosaminidase	ENSG00000108784	0.248	2.50E-02
PHLDB2	pleckstrin homology like domain family B member 2	ENSG00000144824	0.362	2.50E-02
RP11_338I211		ENSG00000271933	-0.395	2.50E-02
C8orf44-SGK3/SGK3	serum/glucocorticoid regulated kinase family member 3	ENSG00000104205	1.588	2.50E-03
EMILIN2	elastin microfibril interfacer 2	ENSG00000132205	-0.828	2.50E-03
NOL3	nucleolar protein 3	ENSG00000140939	-0.582	2.50E-03
ZNF23	zinc finger protein 23	ENSG00000167377	1.588	2.50E-03
ZSWIM5	zinc finger SWIM-type containing 5	ENSG00000162415	0.379	2.50E-03
PSMC3IP	PSMC3 interacting protein	ENSG00000131470	0.685	2.50E-04
AC0073087		ENSG00000272600	1.268	2.60E-02
AC0374451		ENSG00000233635	1.268	2.60E-02
ACVR1C	activin A receptor type 1C	ENSG00000123612	0.686	2.60E-02
APOE	apolipoprotein E	ENSG00000130203	-0.592	2.60E-02
CALM1 (includes others)	calmodulin 1	ENSG00000160014	-0.193	2.60E-02
DIPK1A	divergent protein kinase domain 1A	ENSG00000154511	0.471	2.60E-02
FBXO31	F-box protein 31	ENSG00000103264	0.349	2.60E-02
H3-3A/H3-3B	H3.3 histone A	ENSG00000163041	0.253	2.60E-02
LENG9	leukocyte receptor cluster member 9	ENSG00000275183	0.620	2.60E-02
PKD2L2	polycystin 2 like 2, transient receptor potential cation channel	ENSG00000078795	0.686	2.60E-02
RBM6	RNA binding motif protein 6	ENSG00000004534	-0.250	2.60E-02
RBX1	ring-box 1	ENSG00000100387	-0.322	2.60E-02
RHBDL1	rhomboid like 1	ENSG00000103269	-0.687	2.60E-02
RNA5SP216	RNA, 5S ribosomal pseudogene 216	ENSG00000251920	-1.211	2.60E-02
RP2	RP2 activator of ARL3 GTPase	ENSG00000102218	0.433	2.60E-02
RPS29	ribosomal protein S29	ENSG00000213741	-0.215	2.60E-02
RPS8	ribosomal protein S8	ENSG00000142937	-0.173	2.60E-02
TSNARE1	t-SNARE domain containing 1	ENSG00000171045	-0.619	2.60E-02
ZNF81	zinc finger protein 81	ENSG00000197779	0.686	2.60E-02
ZSCAN30	zinc finger and SCAN domain containing 30	ENSG00000186814	0.447	2.60E-02
MT-RNR1	s-rRNA	ENSG00000211459	-0.285	2.60E-04
CASTOR2	cytosolic arginine sensor for mTORC1 subunit 2	ENSG00000274070	-0.881	2.70E-02
CCDC71L	coiled-coil domain containing 71 like	ENSG00000253276	0.387	2.70E-02
EBAG9	estrogen receptor binding site associated antigen 9	ENSG00000147654	0.652	2.70E-02
GTF2IRD1	GTF2I repeat domain containing 1	ENSG00000006704	-0.375	2.70E-02
HIP1	huntingtin interacting protein 1	ENSG00000127946	-0.251	2.70E-02
INSIG2	insulin induced gene 2	ENSG00000125629	0.471	2.70E-02
MSRB2	methionine sulfoxide reductase B2	ENSG00000148450	-0.397	2.70E-02
MZT2B	mitotic spindle organizing protein 2B	ENSG00000152082	-0.204	2.70E-02

PATZ1	POZ/BTB and AT hook containing zinc finger 1	ENSG00000100105	0.389	2.70E-02
RAPGEFL1	Rap guanine nucleotide exchange factor like 1	ENSG00000108352	-0.764	2.70E-02
RP11-352M15.2		ENSG00000273015	-0.591	2.70E-02
RP11_56G102		ENSG00000257386	-0.425	2.70E-02
RP13_131K191		ENSG00000235236	-0.591	2.70E-02
SLC25A17	solute carrier family 25 member 17	ENSG00000100372	0.586	2.70E-02
TAB3	TGF-beta activated kinase 1 (MAP3K7) binding protein 3	ENSG00000157625	0.387	2.70E-02
TTC26	tetratricopeptide repeat domain 26	ENSG00000105948	-0.591	2.70E-02
ASB3/GPR75-ASB3	ankyrin repeat and SOCS box containing 3	ENSG00000115239	1.259	2.70E-03
EFNB3	ephrin B3	ENSG00000108947	-0.678	2.70E-03
KANK3	KN motif and ankyrin repeat domains 3	ENSG00000186994	-2.393	2.70E-03
RP11_1277A32		ENSG00000246596	2.029	2.70E-03
ST20-MTHFS	ST20-MTHFS readthrough	ENSG00000259332	2.087	2.70E-03
THAP5	THAP domain containing 5	ENSG00000177683	0.703	2.70E-03
ZNF780A	zinc finger protein 780A	ENSG00000197782	0.774	2.70E-03
AAGAB	alpha and gamma adaptin binding protein	ENSG00000103591	0.310	2.80E-02
CCN1	cellular communication network factor 1	ENSG00000142871	0.181	2.80E-02
CTD_2263F211		ENSG00000251257	1.694	2.80E-02
CTPS1	CTP synthase 1	ENSG00000171793	-0.292	2.80E-02
DDX54	DEAD-box helicase 54	ENSG00000123064	0.232	2.80E-02
DUSP1	dual specificity phosphatase 1	ENSG00000120129	0.223	2.80E-02
ELP1	elongator complex protein 1	ENSG00000070061	0.275	2.80E-02
ERBB2	erb-b2 receptor tyrosine kinase 2	ENSG00000141736	0.229	2.80E-02
ERICH3	glutamate rich 3	ENSG00000178965	-1.509	2.80E-02
GAR1	GAR1 ribonucleoprotein	ENSG00000109534	-0.439	2.80E-02
HS2ST1	heparan sulfate 2-O-sulfotransferase 1	ENSG00000153936	0.269	2.80E-02
MBNL3	muscleblind like splicing regulator 3	ENSG00000076770	0.558	2.80E-02
MME	membrane metalloendopeptidase	ENSG00000196549	-0.308	2.80E-02
NNMT	nicotinamide N-methyltransferase	ENSG00000166741	-0.402	2.80E-02
NTAN1	N-terminal asparagine amidase	ENSG00000157045	-0.294	2.80E-02
PARL	presenilin associated rhomboid like	ENSG00000175193	-0.337	2.80E-02
PIGQ	phosphatidylinositol glycan anchor biosynthesis class Q	ENSG00000007541	0.279	2.80E-02
PNPLA7	patatin like phospholipase domain containing 7	ENSG00000130653	1.050	2.80E-02
PRMT3	protein arginine methyltransferase 3	ENSG00000185238	0.412	2.80E-02
RP11_344N102		ENSG00000237768	0.718	2.80E-02
RP11_958N242		ENSG00000227827	0.941	2.80E-02
RP5_1021I205		ENSG00000258891	0.718	2.80E-02
RPSAP47	ribosomal protein SA pseudogene 47	ENSG00000188856	1.089	2.80E-02
SF3A2	splicing factor 3a subunit 2	ENSG00000104897	0.238	2.80E-02
SNRPGP10	small nuclear ribonucleoprotein polypeptide G pseudogene 10	ENSG00000235363	-1.509	2.80E-02
SOCS5	suppressor of cytokine signaling 5	ENSG00000171150	0.319	2.80E-02
TMEM161A	transmembrane protein 161A	ENSG00000064545	-0.406	2.80E-02
WDR12	WD repeat domain 12	ENSG00000138442	0.395	2.80E-02
ZNF48	zinc finger protein 48	ENSG00000180035	-0.487	2.80E-02
ZNF503-AS2	ZNF503 antisense RNA 2	ENSG00000237149	0.481	2.80E-02
ZNF696	zinc finger protein 696	ENSG00000185730	0.441	2.80E-02
GPHN	gephyrin	ENSG00000171723	-0.786	2.80E-03

PRICKLE2-AS3	PRICKLE2 antisense RNA 3	ENSG00000226017	-1.420	2.80E-03
RIOX2	ribosomal oxygenase 2	ENSG00000170854	0.467	2.80E-03
CISD3	CDGSH iron sulfur domain 3	ENSG00000277972	0.296	2.90E-02
EGLN2	egl-9 family hypoxia inducible factor 2	ENSG00000269858	-0.512	2.90E-02
GRIN3B	glutamate ionotropic receptor NMDA type subunit 3B	ENSG00000116032	-0.566	2.90E-02
LARP4	La ribonucleoprotein domain family member 4	ENSG00000161813	0.306	2.90E-02
MAN2B2	mannosidase alpha class 2B member 2	ENSG00000013288	0.252	2.90E-02
MRPS28	mitochondrial ribosomal protein S28	ENSG00000147586	-0.483	2.90E-02
MTRR	5-methyltetrahydrofolate-homocysteine methyltransferase reductase	ENSG00000124275	-0.417	2.90E-02
OXCT1	3-oxoacid CoA-transferase 1	ENSG00000083720	-0.392	2.90E-02
PRKCD	protein kinase C delta	ENSG00000163932	-0.377	2.90E-02
RAMAC	RNA guanine-7 methyltransferase activating subunit	ENSG00000169612	-0.547	2.90E-02
STARD3NL	STARD3 N-terminal like	ENSG00000010270	-0.270	2.90E-02
TGFB2	transforming growth factor beta 2	ENSG00000092969	0.791	2.90E-02
TMEM19	transmembrane protein 19	ENSG00000139291	0.365	2.90E-02
TMEM250	transmembrane protein 250	ENSG00000238227	0.247	2.90E-02
VIRMA	vir like m6A methyltransferase associated	ENSG00000164944	0.259	2.90E-02
XXbac-BPG283O16.9		ENSG00000280128	0.737	2.90E-02
ZNF407	zinc finger protein 407	ENSG00000215421	0.433	2.90E-02
POMT1	protein O-mannosyltransferase 1	ENSG00000130714	-0.445	2.90E-03
TRAF3IP3	TRAF3 interacting protein 3	ENSG00000009790	1.694	2.90E-03
CARD11	caspase recruitment domain family member 11	ENSG00000198286	-0.540	2.90E-04
APOBEC3F	apolipoprotein B mRNA editing enzyme catalytic subunit 3F	ENSG00000128394	0.892	3.00E-02
BICC1	BicC family RNA binding protein 1	ENSG00000122870	0.241	3.00E-02
CRABP2	cellular retinoic acid binding protein 2	ENSG00000143320	-1.344	3.00E-02
CTD_3193O138		ENSG00000269139	0.375	3.00E-02
FAM86C1	family with sequence similarity 86 member C1	ENSG00000158483	-0.828	3.00E-02
MSS51	MSS51 mitochondrial translational activator	ENSG00000166343	1.589	3.00E-02
MTFR1L	mitochondrial fission regulator 1 like	ENSG00000117640	-0.293	3.00E-02
MZF1-AS1	MZF1 antisense RNA 1	ENSG00000267858	0.514	3.00E-02
PMM1	phosphomannomutase 1	ENSG00000100417	-0.367	3.00E-02
PUS10	pseudouridine synthase 10	ENSG00000162927	-0.803	3.00E-02
RP11_283G65		ENSG00000255750	-1.106	3.00E-02
RP11_408P141		ENSG00000243199	-1.344	3.00E-02
RP11_746M11		ENSG00000263986	-0.266	3.00E-02
RP4_530I159		ENSG00000232043	0.692	3.00E-02
TIA1	TIA1 cytotoxic granule associated RNA binding protein	ENSG00000116001	-0.238	3.00E-02
UBAP2	ubiquitin associated protein 2	ENSG00000137073	-0.310	3.00E-02
YTHDF3	YTH N6-methyladenosine RNA binding protein 3	ENSG00000185728	0.239	3.00E-02
ASB16-AS1	ASB16 antisense RNA 1	ENSG00000267080	-0.838	3.00E-03
SPRED1	sprouty related EVH1 domain containing 1	ENSG00000166068	0.281	3.00E-03
STRBP	spermatid perinuclear RNA binding protein	ENSG00000165209	-0.711	3.00E-03
UBQLN4	ubiquilin 4	ENSG00000160803	0.365	3.00E-03
AC0738343		ENSG00000237655	1.151	3.10E-02

CPM	carboxypeptidase M	ENSG00000135678	0.588	3.10E-02
CUL7	cullin 7	ENSG00000044090	0.250	3.10E-02
FAM189B	family with sequence similarity 189 member B	ENSG00000160767	0.307	3.10E-02
HSD17B10	hydroxysteroid 17-beta dehydrogenase 10	ENSG00000072506	-0.300	3.10E-02
HTR1D	5-hydroxytryptamine receptor 1D	ENSG00000179546	-0.497	3.10E-02
KCP	kielin cysteine rich BMP regulator	ENSG00000135253	-0.286	3.10E-02
KLHDC1	kelch domain containing 1	ENSG00000197776	1.352	3.10E-02
MIPEP	mitochondrial intermediate peptidase	ENSG00000027001	0.480	3.10E-02
MSRB3	methionine sulfoxide reductase B3	ENSG00000174099	0.245	3.10E-02
MTMR4	myotubularin related protein 4	ENSG00000108389	0.310	3.10E-02
NPIPA8 (includes others)	nuclear pore complex interacting protein family member A3	ENSG00000183426	0.535	3.10E-02
RCCD1	RCC1 domain containing 1	ENSG00000166965	-0.380	3.10E-02
REXO1	RNA exonuclease 1 homolog	ENSG00000079313	0.289	3.10E-02
RP1_39G227		ENSG00000259943	-1.105	3.10E-02
RPS10P7	ribosomal protein S10 pseudogene 7	ENSG00000223396	-1.306	3.10E-02
SLC25A11	solute carrier family 25 member 11	ENSG00000108528	-0.248	3.10E-02
TRIM73/TRIM74	tripartite motif containing 74	ENSG00000174353	-1.306	3.10E-02
TRIP11	thyroid hormone receptor interactor 11	ENSG00000100815	0.292	3.10E-02
URB1-AS1	URB1 antisense RNA 1 (head to head)	ENSG00000256073	-1.306	3.10E-02
ZBTB33	zinc finger and BTB domain containing 33	ENSG00000177485	0.350	3.10E-02
ZDHHC16	zinc finger DHHC-type containing 16	ENSG00000171307	0.301	3.10E-02
JAZF1-AS1	JAZF1 antisense RNA 1	ENSG00000234336	1.186	3.10E-03
NDUFB8	NADH:ubiquinone oxidoreductase subunit B8	ENSG00000166136	0.518	3.10E-03
ASAH2B	N-acylsphingosine amidohydrolase 2B	ENSG00000204147	1.013	3.20E-02
CAPN10-DT	CAPN10 divergent transcript	ENSG00000260942	-1.762	3.20E-02
CRYZL1	crystallin zeta like 1	ENSG00000205758	-0.382	3.20E-02
EBF1	EBF transcription factor 1	ENSG00000164330	-0.397	3.20E-02
EPHX4	epoxide hydrolase 4	ENSG00000172031	-1.762	3.20E-02
LCA5	lebercilin LCA5	ENSG00000135338	0.605	3.20E-02
LOC100287808	uncharacterized LOC100287808	ENSG00000277182	1.851	3.20E-02
LOC105375304	uncharacterized LOC105375304	ENSG00000225792	1.851	3.20E-02
LRMDA	leucine rich melanocyte differentiation associated	ENSG00000148655	0.756	3.20E-02
LUCAT1	lung cancer associated transcript 1	ENSG00000248323	-1.762	3.20E-02
MN1	MN1 proto-oncogene, transcriptional regulator	ENSG00000169184	0.410	3.20E-02
MTATP6P1	MT-ATP6 pseudogene 1	ENSG00000248527	-0.238	3.20E-02
RETREG1	reticulophagy regulator 1	ENSG00000154153	-0.662	3.20E-02
RP11_304L192		ENSG00000260447	1.851	3.20E-02
RP11_762I75		ENSG00000257390	0.506	3.20E-02
WDR91	WD repeat domain 91	ENSG00000105875	-0.357	3.20E-02
XRCC4	X-ray repair cross complementing 4	ENSG00000152422	-0.597	3.20E-02
ZNF517	zinc finger protein 517	ENSG00000197363	0.572	3.20E-02
ZSCAN21	zinc finger and SCAN domain containing 21	ENSG00000166529	0.756	3.20E-02
CKB	creatine kinase B	ENSG00000166165	-1.789	3.20E-04
MT-CO3	cytochrome c oxidase III	ENSG00000198938	-0.273	3.20E-04
MXI1	MAX interactor 1, dimerization protein	ENSG00000119950	-0.703	3.20E-04
ASRGL1	asparaginase and isoaspartyl peptidase 1	ENSG00000162174	-0.894	3.30E-02
CHMP1B	charged multivesicular body protein 1B	ENSG00000255112	0.267	3.30E-02

CREG1	cellular repressor of E1A stimulated genes 1	ENSG00000143162	0.314	3.30E-02
DNAJC9-AS1	DNAJC9 antisense RNA 1	ENSG00000236756	-0.474	3.30E-02
LA16C_313D1112		ENSG00000261659	-0.547	3.30E-02
MYBL2	MYB proto-oncogene like 2	ENSG00000101057	0.252	3.30E-02
POU6F1	POU class 6 homeobox 1	ENSG00000184271	-0.632	3.30E-02
RP11_1055B88		ENSG00000263271	0.986	3.30E-02
ZNF624	zinc finger protein 624	ENSG00000197566	0.986	3.30E-02
DCAF10	DDB1 and CUL4 associated factor 10	ENSG00000122741	-0.297	3.40E-02
DLGAP4-AS1	DLGAP4 antisense RNA 1	ENSG00000232907	0.334	3.40E-02
GNAQ	G protein subunit alpha q	ENSG00000156052	0.249	3.40E-02
GYPC	glycophorin C (Gerbich blood group)	ENSG00000136732	-0.278	3.40E-02
LINC01605	long intergenic non-protein coding RNA 1605	ENSG00000253414	1.092	3.40E-02
LL22NC03_80A106		ENSG00000272779	-0.997	3.40E-02
NFKBID	NFKB inhibitor delta	ENSG00000167604	1.092	3.40E-02
NMT2	N-myristoyltransferase 2	ENSG00000152465	0.263	3.40E-02
P3H3	prolyl 3-hydroxylase 3	ENSG00000110811	0.550	3.40E-02
RABGGTA	Rab geranylgeranyltransferase subunit alpha	ENSG00000100949	0.406	3.40E-02
RP11_345P49		ENSG00000272106	-0.733	3.40E-02
RP11_436D103		ENSG00000238280	0.576	3.40E-02
RP11_843P142		ENSG00000250909	-0.474	3.40E-02
SEMA5A	semaphorin 5A	ENSG00000112902	0.203	3.40E-02
SHANK3	SH3 and multiple ankyrin repeat domains 3	ENSG00000251322	0.461	3.40E-02
SOX15	SRY-box transcription factor 15	ENSG00000129194	-0.997	3.40E-02
TMSB4XP4	TMSB4X pseudogene 4	ENSG00000223551	0.533	3.40E-02
TNFAIP2	TNF alpha induced protein 2	ENSG00000185215	-0.733	3.40E-02
UPP1	uridine phosphorylase 1	ENSG00000183696	-0.230	3.40E-02
WDR47	WD repeat domain 47	ENSG00000085433	-0.344	3.40E-02
WLS	Wnt ligand secretion mediator	ENSG00000116729	-0.179	3.40E-02
ZNF800	zinc finger protein 800	ENSG00000048405	0.517	3.40E-02
G0S2	G0/G1 switch 2	ENSG00000123689	-0.993	3.40E-03
AP0014695		ENSG00000223901	0.640	3.50E-02
ATG14	autophagy related 14	ENSG00000126775	0.335	3.50E-02
DTNBP1	dystrobrevin binding protein 1	ENSG00000047579	0.611	3.50E-02
EIF4B	eukaryotic translation initiation factor 4B	ENSG00000063046	0.171	3.50E-02
LZTS3	leucine zipper tumor suppressor family member 3	ENSG00000088899	-0.481	3.50E-02
MRPL40	mitochondrial ribosomal protein L40	ENSG00000185608	-0.548	3.50E-02
MTCO1P12	MT-CO1 pseudogene 12	ENSG00000237973	0.491	3.50E-02
NT5E	5'-nucleotidase ecto	ENSG00000135318	-0.218	3.50E-02
PARP11	poly(ADP-ribose) polymerase family member 11	ENSG00000111224	0.555	3.50E-02
RAB10	RAB10, member RAS oncogene family	ENSG00000084733	0.201	3.50E-02
RP11_18I1411		ENSG00000273262	0.386	3.50E-02
RP11_304L198		ENSG00000261532	-0.547	3.50E-02
RP11_347C1211		ENSG00000274653	0.803	3.50E-02
RP11_649E75		ENSG00000258377	0.235	3.50E-02
SEMA6D	semaphorin 6D	ENSG00000137872	0.492	3.50E-02
SSBP3	single stranded DNA binding protein 3	ENSG00000157216	-0.291	3.50E-02
USP38	ubiquitin specific peptidase 38	ENSG00000170185	0.315	3.50E-02
VMA21	vacuolar ATPase assembly factor VMA21	ENSG00000160131	-0.326	3.50E-02
ZFP36	ZFP36 ring finger protein	ENSG00000128016	0.640	3.50E-02

MMP15	matrix metallopeptidase 15	ENSG00000102996	0.608	3.50E-03
ZNF416	zinc finger protein 416	ENSG00000083817	1.076	3.50E-03
RP11_135F93		ENSG00000280088	2.601	3.50E-04
AC0071926		ENSG00000269145	1.453	3.60E-02
BRICD5	BRICHOS domain containing 5	ENSG00000182685	-0.528	3.60E-02
CACNA1C-AS1	CACNA1C antisense RNA 1	ENSG00000246627	-1.813	3.60E-02
CCDC144NL-AS1	CCDC144NL antisense RNA 1	ENSG00000233098	0.250	3.60E-02
CCDC181	coiled-coil domain containing 181	ENSG00000117477	-1.813	3.60E-02
CTB_79E83		ENSG00000253683	2.222	3.60E-02
DDX28	DEAD-box helicase 28	ENSG00000182810	-0.469	3.60E-02
DICER1-AS1	DICER1 antisense RNA 1	ENSG00000235706	-1.074	3.60E-02
FAM72C/FAM72D	family with sequence similarity 72 member D	ENSG00000263513	-1.813	3.60E-02
FUT2	fucosyltransferase 2	ENSG00000176920	-1.813	3.60E-02
HIBCH	3-hydroxyisobutyryl-CoA hydrolase	ENSG00000198130	0.453	3.60E-02
LINC01572	long intergenic non-protein coding RNA 1572	ENSG00000261008	2.222	3.60E-02
LOC100130548	uncharacterized LOC100130548	ENSG00000235138	2.222	3.60E-02
LOC339192	uncharacterized LOC339192	ENSG00000267121	2.222	3.60E-02
MIATNB	MIAT neighbor	ENSG00000244625	-1.419	3.60E-02
MLPH	melanophilin	ENSG00000115648	-0.693	3.60E-02
OR7E38P	olfactory receptor family 7 subfamily E member 38 pseudogene	ENSG00000183444	-1.813	3.60E-02
OSBP	oxysterol binding protein	ENSG00000110048	0.241	3.60E-02
PCDHGB1	protocadherin gamma subfamily B, 1	ENSG00000254221	0.453	3.60E-02
PHB	prohibitin	ENSG00000167085	-0.207	3.60E-02
PPCS	phosphopantothenoylcysteine synthetase	ENSG00000127125	0.327	3.60E-02
RP11_135A244		ENSG00000233825	-0.797	3.60E-02
RP11_316O141		ENSG00000268603	2.222	3.60E-02
RP11_332H171		ENSG00000232959	0.927	3.60E-02
RP11_797A183		ENSG00000259652	2.222	3.60E-02
TMEM126A	transmembrane protein 126A	ENSG00000171202	-0.449	3.60E-02
UQCRFS1	ubiquinol-cytochrome c reductase, Rieske iron-sulfur polypeptide 1	ENSG00000169021	0.277	3.60E-02
ZNF205	zinc finger protein 205	ENSG00000122386	-0.404	3.60E-02
ZNF497	zinc finger protein 497	ENSG00000174586	-0.458	3.60E-02
ZNF605	zinc finger protein 605	ENSG00000196458	-0.449	3.60E-02
CTD_2524L63		ENSG00000260037	-1.985	3.60E-04
AP0007045		ENSG00000224790	1.216	3.70E-02
C5orf34	chromosome 5 open reading frame 34	ENSG00000172244	0.767	3.70E-02
HCG4P7	HLA complex group 4 pseudogene 7	ENSG00000230521	-0.174	3.70E-02
HUNK	hormonally up-regulated Neu-associated kinase	ENSG00000142149	0.437	3.70E-02
IGSF9	immunoglobulin superfamily member 9	ENSG00000085552	-1.141	3.70E-02
MED19	mediator complex subunit 19	ENSG00000156603	-0.441	3.70E-02
MORF4L2-AS1	MORF4L2 antisense RNA 1	ENSG00000231154	1.395	3.70E-02
MTRNR2L12	MT-RNR2 like 12	ENSG00000269028	-1.282	3.70E-02
MYEF2	myelin expression factor 2	ENSG00000104177	-0.297	3.70E-02
PAXIP1-AS1	PAXIP1 antisense RNA 1 (head to head)	ENSG00000273344	0.767	3.70E-02
RP11_23P136		ENSG00000174171	1.395	3.70E-02
RP11_257O52		ENSG00000279059	-1.282	3.70E-02
RP11_483I135		ENSG00000260879	0.673	3.70E-02
STK16	serine/threonine kinase 16	ENSG00000115661	0.320	3.70E-02

SYDE2	synapse defective Rho GTPase homolog 2	ENSG00000097096	0.767	3.70E-02
TGDS	TDP-glucose 4,6-dehydratase	ENSG00000088451	0.692	3.70E-02
USP53	ubiquitin specific peptidase 53	ENSG00000145390	0.325	3.70E-02
XPO4	exportin 4	ENSG00000132953	0.349	3.70E-02
ZNF449	zinc finger protein 449	ENSG00000173275	-0.679	3.70E-02
AC1147307		ENSG00000234793	1.305	3.70E-03
AREG	amphiregulin	ENSG00000109321	1.010	3.80E-02
CTTNBP2NL	CTTNBP2 N-terminal like	ENSG00000143079	-0.270	3.80E-02
FNIP1	folliculin interacting protein 1	ENSG00000217128	0.322	3.80E-02
MCCC1	methylcrotonoyl-CoA carboxylase 1	ENSG00000078070	0.423	3.80E-02
MED15	mediator complex subunit 15	ENSG00000099917	-0.248	3.80E-02
MGARP	mitochondria localized glutamic acid rich protein	ENSG00000137463	0.239	3.80E-02
NDUFV3	NADH:ubiquinone oxidoreductase subunit V3	ENSG00000160194	-0.383	3.80E-02
RAB24	RAB24, member RAS oncogene family	ENSG00000169228	-0.375	3.80E-02
RGL4	ral guanine nucleotide dissociation stimulator like 4	ENSG00000159496	-0.976	3.80E-02
RP5_1057I204		ENSG00000268069	0.452	3.80E-02
RRAD	RRAD, Ras related glycolysis inhibitor and calcium channel regulator	ENSG00000166592	-0.242	3.80E-02
RTN2	reticulon 2	ENSG00000125744	-0.417	3.80E-02
SLC29A1	solute carrier family 29 member 1 (Augustine blood group)	ENSG00000112759	-0.260	3.80E-02
SRRM5	serine/arginine repetitive matrix 5	ENSG00000226763	0.294	3.80E-02
STX12	syntaxin 12	ENSG00000117758	-0.348	3.80E-02
SARNP	SAP domain containing ribonucleoprotein	ENSG00000205323	-1.462	3.80E-03
AC0159332		ENSG00000227279	-0.310	3.90E-02
AFG3L1P	AFG3 like matrix AAA peptidase subunit 1, pseudogene	ENSG00000223959	0.403	3.90E-02
ANGEL2	angel homolog 2	ENSG00000174606	0.359	3.90E-02
BAG4	BCL2 associated athanogene 4	ENSG00000156735	-0.389	3.90E-02
BAK1	BCL2 antagonist/killer 1	ENSG00000030110	0.352	3.90E-02
BTRC	beta-transducin repeat containing E3 ubiquitin protein ligase	ENSG00000166167	-0.349	3.90E-02
CH17_264L241		ENSG00000279278	-2.061	3.90E-02
DCK	deoxycytidine kinase	ENSG00000156136	0.376	3.90E-02
DDX31	DEAD-box helicase 31	ENSG00000125485	0.452	3.90E-02
DNASE2	deoxyribonuclease 2, lysosomal	ENSG00000105612	0.273	3.90E-02
ENAH	ENAH actin regulator	ENSG00000154380	0.158	3.90E-02
GOLT1B	golgi transport 1B	ENSG00000111711	0.299	3.90E-02
HOXA2	homeobox A2	ENSG00000105996	2.933	3.90E-02
LDAH	lipid droplet associated hydrolase	ENSG00000118961	0.563	3.90E-02
LOC105372097		ENSG00000270112	-2.061	3.90E-02
PCDHB13	protocadherin beta 13	ENSG00000187372	0.416	3.90E-02
RBM25	RNA binding motif protein 25	ENSG00000119707	-0.222	3.90E-02
ROGDI	rogdi atypical leucine zipper	ENSG00000067836	0.451	3.90E-02
RP11_251G235		ENSG00000272604	-2.061	3.90E-02
RP11_495P103		ENSG00000224481	-2.061	3.90E-02
RP11_513D52		ENSG00000254898	0.764	3.90E-02
SPATC1L	spermatogenesis and centriole associated 1 like	ENSG00000160284	0.482	3.90E-02
TMEM14A	transmembrane protein 14A	ENSG00000096092	0.372	3.90E-02

TMPRSS6	transmembrane serine protease 6	ENSG00000187045	-2.061	3.90E-02
USF3	upstream transcription factor family member 3	ENSG00000176542	0.399	3.90E-02
ZNF730	zinc finger protein 730	ENSG00000183850	-2.061	3.90E-02
ZNF574	zinc finger protein 574	ENSG00000105732	0.492	3.90E-03
ANKLE1	ankyrin repeat and LEM domain containing 1	ENSG00000160117	2.524	4.00E-02
ASGR1	asialoglycoprotein receptor 1	ENSG00000141505	2.524	4.00E-02
CHRM3	cholinergic receptor muscarinic 3	ENSG00000133019	2.524	4.00E-02
COMMD5	COMM domain containing 5	ENSG00000170619	-0.293	4.00E-02
EHHADH-AS1	EHHADH antisense RNA 1	ENSG00000223358	2.524	4.00E-02
FAM162A	family with sequence similarity 162 member A	ENSG00000114023	-0.414	4.00E-02
FEM1B	fem-1 homolog B	ENSG00000169018	0.226	4.00E-02
GFPT2	glutamine-fructose-6-phosphate transaminase 2	ENSG00000131459	-0.322	4.00E-02
GPR173	G protein-coupled receptor 173	ENSG00000184194	0.537	4.00E-02
HMGN1P38	high mobility group nucleosome binding domain 1 pseudogene 38	ENSG00000253954	2.524	4.00E-02
KCNH3	potassium voltage-gated channel subfamily H member 3	ENSG00000135519	0.872	4.00E-02
LA16C_316G122		ENSG00000260425	2.524	4.00E-02
LA16C_360H63		ENSG00000262899	2.524	4.00E-02
MIS18A	MIS18 kinetochore protein A	ENSG00000159055	-0.414	4.00E-02
MZT2A	mitotic spindle organizing protein 2A	ENSG00000173272	-0.246	4.00E-02
PADI1	peptidyl arginine deiminase 1	ENSG00000142623	2.524	4.00E-02
PSORS1C2	psoriasis susceptibility 1 candidate 2	ENSG00000204538	2.524	4.00E-02
RP11-596C23.6		ENSG00000282885	2.524	4.00E-02
RP11_288I211		ENSG00000237938	-0.457	4.00E-02
RP11_29G83		ENSG00000261553	2.524	4.00E-02
RP11_677M145		ENSG00000254941	2.524	4.00E-02
RP1_239B225		ENSG00000260196	2.524	4.00E-02
SLC9A1	solute carrier family 9 member A1	ENSG00000090020	-0.275	4.00E-02
SULT1B1	sulfotransferase family 1B member 1	ENSG00000173597	2.524	4.00E-02
TGIF1	TGFB induced factor homeobox 1	ENSG00000177426	-0.236	4.00E-02
UBOX5	U-box domain containing 5	ENSG00000185019	0.506	4.00E-02
ZNF442	zinc finger protein 442	ENSG00000198342	2.524	4.00E-02
ZNF625	zinc finger protein 625	ENSG00000257591	2.524	4.00E-02
POTEI	POTE ankyrin domain family member I	ENSG00000196834	6.103	4.00E-03
RP11_428J14		ENSG00000271978	6.103	4.00E-03
TBX20	T-box transcription factor 20	ENSG00000164532	6.103	4.00E-03
ANKRD18B	ankyrin repeat domain 18B	ENSG00000230453	1.141	4.10E-02
CAPS2	calcyphosine 2	ENSG00000180881	0.614	4.10E-02
CEP78	centrosomal protein 78	ENSG00000148019	-0.314	4.10E-02
GMNN	geminin DNA replication inhibitor	ENSG00000112312	-0.338	4.10E-02
LOC101927572	uncharacterized LOC101927572	ENSG00000267698	0.614	4.10E-02
MOSMO	modulator of smoothened	ENSG00000185716	-0.465	4.10E-02
MRPL22	mitochondrial ribosomal protein L22	ENSG00000082515	-0.355	4.10E-02
PKD1P5	polycystin 1, transient receptor potential channel interacting pseudogene 5	ENSG00000254681	0.350	4.10E-02
PMS2P1	PMS1 homolog 2, mismatch repair system component pseudogene 1	ENSG00000078319	-0.576	4.10E-02
PRRG4	proline rich and Gla domain 4	ENSG00000135378	1.141	4.10E-02

RPL27	ribosomal protein L27	ENSG00000131469	-0.169	4.10E-02
TSPAN4	tetraspanin 4	ENSG00000214063	-0.216	4.10E-02
CCDC68	coiled-coil domain containing 68	ENSG00000166510	-0.605	4.10E-03
LINC01391	long intergenic non-protein coding RNA 1391	ENSG00000244578	0.506	4.10E-03
TMEFF2	transmembrane protein with EGF like and two follistatin like domains 2	ENSG00000144339	0.799	4.10E-03
A1BG-AS1	A1BG antisense RNA 1	ENSG00000268895	-0.548	4.20E-02
AC00913315		ENSG00000259952	0.213	4.20E-02
CLDN12	claudin 12	ENSG00000157224	-0.548	4.20E-02
HMGN3	high mobility group nucleosomal binding domain 3	ENSG00000118418	-0.284	4.20E-02
LINC02693	long intergenic non-protein coding RNA 2693	ENSG00000212719	0.405	4.20E-02
LOC101928140	uncharacterized LOC101928140	ENSG00000227945	0.444	4.20E-02
MAP3K7	mitogen-activated protein kinase kinase kinase 7	ENSG00000135341	0.261	4.20E-02
NAA40	N(alpha)-acetyltransferase 40, NatD catalytic subunit	ENSG00000110583	-0.325	4.20E-02
PSD3	pleckstrin and Sec7 domain containing 3	ENSG00000156011	0.172	4.20E-02
PTPDC1	protein tyrosine phosphatase domain containing 1	ENSG00000158079	-0.550	4.20E-02
SNRNP25	small nuclear ribonucleoprotein U11/U12 subunit 25	ENSG00000161981	-0.325	4.20E-02
TMEM25	transmembrane protein 25	ENSG00000149582	0.359	4.20E-02
TSPAN11	tetraspanin 11	ENSG00000110900	0.375	4.20E-02
YPEL2	yippee like 2	ENSG00000175155	0.452	4.20E-02
ZACN	zinc activated ion channel	ENSG00000186919	-0.217	4.20E-02
LOXL4	lysyl oxidase like 4	ENSG00000138131	0.700	4.20E-03
PPAT	phosphoribosyl pyrophosphate amidotransferase	ENSG00000128059	0.726	4.20E-04
ARHGAP5	Rho GTPase activating protein 5	ENSG00000100852	0.267	4.30E-02
BCDIN3D-AS1	BCDIN3D antisense RNA 1	ENSG00000258057	0.700	4.30E-02
BST2	bone marrow stromal cell antigen 2	ENSG00000130303	0.936	4.30E-02
ENGASE	endo-beta-N-acetylglucosaminidase	ENSG00000167280	0.317	4.30E-02
ERI1	exoribonuclease 1	ENSG00000104626	-0.411	4.30E-02
GCC1	GRIP and coiled-coil domain containing 1	ENSG00000179562	0.312	4.30E-02
GUCY1A2	guanylate cyclase 1 soluble subunit alpha 2	ENSG00000152402	1.100	4.30E-02
LAMTOR3	late endosomal/lysosomal adaptor, MAPK and MTOR activator 3	ENSG00000109270	0.378	4.30E-02
LINC-PINT	long intergenic non-protein coding RNA, p53 induced transcript	ENSG00000226380	-1.620	4.30E-02
LINC00674	long intergenic non-protein coding RNA 674	ENSG00000237854	0.622	4.30E-02
LOC101928728	uncharacterized LOC101928728	ENSG00000260063	-1.620	4.30E-02
LOC102724050	uncharacterized LOC102724050	ENSG00000258086	0.336	4.30E-02
LOC439933	uncharacterized LOC439933	ENSG00000247193	1.535	4.30E-02
LRRC2	leucine rich repeat containing 2	ENSG00000163827	0.387	4.30E-02
NFIA	nuclear factor I A	ENSG00000162599	0.819	4.30E-02
RP11_289I102		ENSG00000223612	1.617	4.30E-02
RP11_517O131		ENSG00000258378	-0.815	4.30E-02
RP11_582J163		ENSG00000254230	0.310	4.30E-02
SENP8	SUMO peptidase family member, NEDD8	ENSG00000166192	-0.815	4.30E-02

	specific			
SLC4A5	solute carrier family 4 member 5	ENSG00000188687	-0.815	4.30E-02
SNX12	sorting nexin 12	ENSG00000147164	0.236	4.30E-02
SPATA6	spermatogenesis associated 6	ENSG00000132122	0.822	4.30E-02
TEAD1	TEA domain transcription factor 1	ENSG00000187079	0.173	4.30E-02
TNFRSF11B	TNF receptor superfamily member 11b	ENSG00000164761	0.622	4.30E-02
TRIT1	tRNA isopentenyltransferase 1	ENSG00000043514	0.495	4.30E-02
TRPV4	transient receptor potential cation channel subfamily V member 4	ENSG00000111199	-0.781	4.30E-02
ZBTB21	zinc finger and BTB domain containing 21	ENSG00000173276	0.347	4.30E-02
ZNF345	zinc finger protein 345	ENSG00000251247	-1.403	4.30E-02
ZNF7	zinc finger protein 7	ENSG00000147789	-0.374	4.30E-02
ZSCAN5A	zinc finger and SCAN domain containing 5A	ENSG00000131848	0.822	4.30E-02
COL24A1	collagen type XXIV alpha 1 chain	ENSG00000171502	-1.580	4.30E-03
HOXA-AS3	HOXA cluster antisense RNA 3	ENSG00000254369	-1.091	4.30E-03
MTERF4	mitochondrial transcription termination factor 4	ENSG00000122085	0.522	4.30E-03
NOXO1	NADPH oxidase organizer 1	ENSG00000196408	-1.091	4.30E-03
RP11_486F171		ENSG00000257045	-1.580	4.30E-03
RP4_742J242		ENSG00000236526	1.011	4.30E-03
AC0742125		ENSG00000259605	0.305	4.40E-02
ANXA4	annexin A4	ENSG00000196975	-0.236	4.40E-02
BLMH	bleomycin hydrolase	ENSG00000108578	-0.294	4.40E-02
DDAH2	dimethylarginine dimethylaminohydrolase 2	ENSG00000213722	0.311	4.40E-02
EMC6	ER membrane protein complex subunit 6	ENSG00000127774	-0.295	4.40E-02
RP11_152P232		ENSG00000260350	-0.412	4.40E-02
RP11_566K114		ENSG00000259006	-0.908	4.40E-02
SLC7A11	solute carrier family 7 member 11	ENSG00000151012	0.182	4.40E-02
STS	steroid sulfatase	ENSG00000101846	0.420	4.40E-02
TGM2	transglutaminase 2	ENSG00000198959	-0.303	4.40E-02
ZSCAN25	zinc finger and SCAN domain containing 25	ENSG00000197037	-0.575	4.40E-02
CTD_2017D111		ENSG00000268362	-1.787	4.40E-03
DAW1	dynein assembly factor with WD repeats 1	ENSG00000123977	3.344	4.40E-03
DLEC1	DLEC1 cilia and flagella associated protein	ENSG00000008226	0.656	4.40E-03
LOC101928663	uncharacterized LOC101928663	ENSG00000168405	-2.471	4.40E-03
PPP1R35	protein phosphatase 1 regulatory subunit 35	ENSG00000160813	-0.538	4.40E-03
RP11_592N211		ENSG00000212664	1.968	4.40E-03
ZNF197	zinc finger protein 197	ENSG00000186448	0.494	4.40E-03
AC0021171		ENSG00000224505	-1.232	4.40E-04
AC022007.1		ENSG00000206567	1.523	4.50E-02
ANKH	ANKH inorganic pyrophosphate transport regulator	ENSG00000154122	-0.195	4.50E-02
C1orf112	chromosome 1 open reading frame 112	ENSG00000000460	0.344	4.50E-02
C1QTNF6	C1q and TNF related 6	ENSG00000133466	0.471	4.50E-02
DCHS1	dachsous cadherin-related 1	ENSG00000166341	-1.228	4.50E-02
ENO2	enolase 2	ENSG00000111674	-0.373	4.50E-02
H2BC5	H2B clustered histone 5	ENSG00000158373	1.295	4.50E-02
NEK10	NIMA related kinase 10	ENSG00000163491	1.523	4.50E-02
OLFML2B	olfactomedin like 2B	ENSG00000162745	1.295	4.50E-02

OXA1L	OXA1L mitochondrial inner membrane protein	ENSG00000155463	-0.247	4.50E-02
RAB3A	RAB3A, member RAS oncogene family	ENSG00000105649	1.295	4.50E-02
RILPL2	Rab interacting lysosomal protein like 2	ENSG00000150977	0.441	4.50E-02
RP11_240G225		ENSG00000274554	1.523	4.50E-02
RP11_413H222		ENSG00000260621	0.441	4.50E-02
RP11_537A69		ENSG00000233144	-0.344	4.50E-02
RPL30	ribosomal protein L30	ENSG00000156482	-0.169	4.50E-02
RPS17	ribosomal protein S17	ENSG00000182774	-0.161	4.50E-02
SCAND2P	SCAN domain containing 2 pseudogene	ENSG00000176700	0.300	4.50E-02
SERPINF1	serpin family F member 1	ENSG00000132386	1.523	4.50E-02
SLFN12	schlafen family member 12	ENSG00000172123	-0.373	4.50E-02
STMP1	short transmembrane mitochondrial protein 1	ENSG00000243317	-0.287	4.50E-02
TGS1	trimethylguanosine synthase 1	ENSG00000137574	-0.328	4.50E-02
TMCO6	transmembrane and coiled-coil domains 6	ENSG00000113119	0.654	4.50E-02
TMUB2	transmembrane and ubiquitin like domain containing 2	ENSG00000168591	-0.302	4.50E-02
TTC3-AS1	TTC3 antisense RNA 1	ENSG00000228677	-0.446	4.50E-02
ZC3H4	zinc finger CCCH-type containing 4	ENSG00000130749	0.222	4.50E-02
CTC_506B81		ENSG00000249180	-0.495	4.50E-03
MKKS	McKusick-Kaufman syndrome	ENSG00000125863	-0.470	4.50E-03
PIN4	peptidylprolyl cis/trans isomerase, NIMA-interacting 4	ENSG00000102309	-0.588	4.50E-03
ARAP1	ArfGAP with RhoGAP domain, ankyrin repeat and PH domain 1	ENSG00000186635	0.200	4.60E-02
ASTE1	asteroid homolog 1	ENSG00000034533	0.419	4.60E-02
CIPC	CLOCK interacting pacemaker	ENSG00000198894	-0.456	4.60E-02
DIXDC1	DIX domain containing 1	ENSG00000150764	0.346	4.60E-02
FBXO48	F-box protein 48	ENSG00000204923	1.049	4.60E-02
HAS3	hyaluronan synthase 3	ENSG00000103044	0.351	4.60E-02
METTL17	methyltransferase like 17	ENSG00000165792	-0.285	4.60E-02
PHLPP1	PH domain and leucine rich repeat protein phosphatase 1	ENSG00000081913	-0.493	4.60E-02
RAB30	RAB30, member RAS oncogene family	ENSG00000137502	-0.384	4.60E-02
RASSF2	Ras association domain family member 2	ENSG00000101265	0.525	4.60E-02
RP11-96L14.7		ENSG00000236782	0.405	4.60E-02
RP11_758P172		ENSG00000241357	-0.650	4.60E-02
WDR83OS	WD repeat domain 83 opposite strand	ENSG00000105583	0.242	4.60E-02
ZNF26	zinc finger protein 26	ENSG00000198393	0.572	4.60E-02
ELOVL2	ELOVL fatty acid elongase 2	ENSG00000197977	2.241	4.60E-03
H2AZ1-DT	H2AZ1 divergent transcript	ENSG00000245322	-0.352	4.60E-04
ABCF2	ATP binding cassette subfamily F member 2	ENSG00000033050	-0.239	4.70E-02
CLASRP	CLK4 associating serine/arginine rich protein	ENSG00000104859	-0.240	4.70E-02
CTTNBP2	cortactin binding protein 2	ENSG00000077063	0.742	4.70E-02
CUL1	cullin 1	ENSG00000055130	-0.233	4.70E-02
FZD1	frizzled class receptor 1	ENSG00000157240	-0.295	4.70E-02
GPATCH1	G-patch domain containing 1	ENSG00000076650	-0.528	4.70E-02
KLHL26	kelch like family member 26	ENSG00000167487	0.548	4.70E-02
LPCAT4	lysophosphatidylcholine acyltransferase 4	ENSG00000176454	-0.331	4.70E-02
PPARA	peroxisome proliferator activated receptor alpha	ENSG00000186951	0.386	4.70E-02

PTPRU	protein tyrosine phosphatase receptor type U	ENSG00000060656	0.346	4.70E-02
TAF8	TATA-box binding protein associated factor 8	ENSG00000137413	-0.339	4.70E-02
TNFSF4	TNF superfamily member 4	ENSG00000117586	0.494	4.70E-02
UACA	uveal autoantigen with coiled-coil domains and ankyrin repeats	ENSG00000137831	-0.222	4.70E-02
NUDT15	nudix hydrolase 15	ENSG00000136159	-0.550	4.70E-03
NXN	nucleoredoxin	ENSG00000167693	0.440	4.70E-03
RP4_773N104		ENSG00000258634	-0.743	4.70E-03
TBC1D10A	TBC1 domain family member 10A	ENSG00000099992	0.717	4.70E-03
CDKN2AIP	CDKN2A interacting protein	ENSG00000168564	0.632	4.70E-04
AC0052622		ENSG00000267688	-0.340	4.80E-02
AC005301.9		ENSG00000283633	0.860	4.80E-02
DNAJC25	DnaJ heat shock protein family (Hsp40) member C25	ENSG00000059769	-0.610	4.80E-02
FAM72A	family with sequence similarity 72 member A	ENSG00000196550	0.860	4.80E-02
FOXP4	forkhead box P4	ENSG00000137166	0.239	4.80E-02
GON7	GON7 subunit of KEOPS complex	ENSG00000170270	-0.658	4.80E-02
GSDMB	gasdermin B	ENSG00000073605	-0.835	4.80E-02
HECA	hdc homolog, cell cycle regulator	ENSG00000112406	0.291	4.80E-02
LOC103611081	uncharacterized LOC103611081	ENSG00000255455	0.645	4.80E-02
MAL2	mal, T cell differentiation protein 2 (gene/pseudogene)	ENSG00000147676	-0.886	4.80E-02
MED17	mediator complex subunit 17	ENSG00000042429	0.304	4.80E-02
MICU3	mitochondrial calcium uptake family member 3	ENSG00000155970	0.893	4.80E-02
NEXN-AS1	NEXN antisense RNA 1	ENSG00000235927	-0.647	4.80E-02
POLH	DNA polymerase eta	ENSG00000170734	0.354	4.80E-02
PPIL4	peptidylprolyl isomerase like 4	ENSG00000131013	-0.341	4.80E-02
PRRX1	paired related homeobox 1	ENSG00000116132	-0.320	4.80E-02
RAB11FIP1P1	RAB11 family interacting protein 1 pseudogene 1	ENSG00000228492	-0.886	4.80E-02
RAP1GAP2	RAP1 GTPase activating protein 2	ENSG00000132359	0.630	4.80E-02
REXO2	RNA exonuclease 2	ENSG00000076043	-0.192	4.80E-02
SOAT1	sterol O-acyltransferase 1	ENSG00000057252	0.275	4.80E-02
STK26	serine/threonine kinase 26	ENSG00000134602	0.471	4.80E-02
STX5	syntaxin 5	ENSG00000162236	0.244	4.80E-02
TMEM134	transmembrane protein 134	ENSG00000172663	0.321	4.80E-02
U731662		ENSG00000230454	-0.835	4.80E-02
ZNF121	zinc finger protein 121	ENSG00000197961	-0.415	4.80E-02
NT5C	5', 3'-nucleotidase, cytosolic	ENSG00000125458	-0.371	4.80E-03
ARSJ	arylsulfatase family member J	ENSG00000180801	0.460	4.90E-02
C12orf65	chromosome 12 open reading frame 65	ENSG00000130921	-0.360	4.90E-02
C3orf38	chromosome 3 open reading frame 38	ENSG00000179021	0.357	4.90E-02
CFAP97	cilia and flagella associated protein 97	ENSG00000164323	-0.263	4.90E-02
GNAS	GNAS complex locus	ENSG00000087460	-0.152	4.90E-02
RANGRF	RAN guanine nucleotide release factor	ENSG00000108961	-0.394	4.90E-02
SCLT1	sodium channel and clathrin linker 1	ENSG00000151466	-0.416	4.90E-02
TGFBI	transforming growth factor beta induced	ENSG00000120708	-0.229	4.90E-02
TRAPPC5	trafficking protein particle complex 5	ENSG00000181029	-0.552	4.90E-02
WTAP	WT1 associated protein	ENSG00000146457	-0.229	4.90E-02

ABCA8	ATP binding cassette subfamily A member 8	ENSG00000141338	-0.550	5.00E-02
AC0052532		ENSG00000268030	-0.507	5.00E-02
ANXA10	annexin A10	ENSG00000109511	0.497	5.00E-02
ARHGAP24	Rho GTPase activating protein 24	ENSG00000138639	0.273	5.00E-02
BFSP1	beaded filament structural protein 1	ENSG00000125864	-1.637	5.00E-02
CAND2	cullin associated and neddylation dissociated 2 (putative)	ENSG00000144712	1.759	5.00E-02
FAM102B	family with sequence similarity 102 member B	ENSG00000162636	0.460	5.00E-02
FSD1L	fibronectin type III and SPRY domain containing 1 like	ENSG00000106701	-0.671	5.00E-02
FSIP1	fibrous sheath interacting protein 1	ENSG00000150667	1.759	5.00E-02
LOC729732	uncharacterized LOC729732	ENSG00000283674	1.759	5.00E-02
LRIG3	leucine rich repeats and immunoglobulin like domains 3	ENSG00000139263	-1.007	5.00E-02
MLH3	mutL homolog 3	ENSG00000119684	0.399	5.00E-02
MT-ND4	NADH dehydrogenase, subunit 4 (complex I)	ENSG00000198886	-0.147	5.00E-02
NDUFA2	NADH:ubiquinone oxidoreductase subunit A2	ENSG00000131495	-0.268	5.00E-02
NDUFAF3	NADH:ubiquinone oxidoreductase complex assembly factor 3	ENSG00000178057	0.255	5.00E-02
PLEKHM3	pleckstrin homology domain containing M3	ENSG00000178385	-0.405	5.00E-02
RP11_143K117		ENSG00000277728	-1.007	5.00E-02
RP11_144G612		ENSG00000254929	-1.637	5.00E-02
RP11_334E610		ENSG00000245385	-0.361	5.00E-02
RP11_481J24		ENSG00000276259	-0.581	5.00E-02
RP11_65L32		ENSG00000270277	-1.637	5.00E-02
RPL35	ribosomal protein L35	ENSG00000136942	-0.162	5.00E-02
RPL37A	ribosomal protein L37a	ENSG00000197756	-0.157	5.00E-02
SFI1	SFI1 centrin binding protein	ENSG00000198089	0.388	5.00E-02
SPIN1	spindlin 1	ENSG00000106723	0.195	5.00E-02
TCEAL9	transcription elongation factor A like 9	ENSG00000185222	0.229	5.00E-02
XKR6	XK related 6	ENSG00000171044	0.570	5.00E-02
CABLES1	Cdk5 and Abl enzyme substrate 1	ENSG00000134508	0.553	5.00E-03
XYLB	xylulokinase	ENSG00000093217	1.727	5.00E-03
TFPI2	tissue factor pathway inhibitor 2	ENSG00000105825	-1.261	5.00E-04
LINC01138	long intergenic non-protein coding RNA 1138	ENSG00000274020	0.897	5.10E-03
RASSF10	Ras association domain family member 10	ENSG00000189431	-6.761	5.10E-04
ARHGEF28	Rho guanine nucleotide exchange factor 28	ENSG00000214944	-0.434	5.20E-04
MYLIP	myosin regulatory light chain interacting protein	ENSG00000007944	-0.817	5.30E-04
ING1	inhibitor of growth family member 1	ENSG00000153487	-0.421	5.40E-03
SF3A3	splicing factor 3a subunit 3	ENSG00000183431	-0.314	5.40E-03
SPACA6	sperm acrosome associated 6	ENSG00000182310	-0.642	5.40E-03
TAF1A	TATA-box binding protein associated factor, RNA polymerase I subunit A	ENSG00000143498	-1.321	5.40E-04
PIAS4	protein inhibitor of activated STAT 4	ENSG00000105229	0.428	5.50E-03
ANKRD34A	ankyrin repeat domain 34A	ENSG00000272031	1.997	5.50E-04
ATP5PF	ATP synthase peripheral stalk subunit F6	ENSG00000154723	-0.341	5.60E-03
HAND2-AS1	HAND2 antisense RNA 1	ENSG00000237125	-1.664	5.60E-03

MAT2B	methionine adenosyltransferase 2B	ENSG00000038274	-0.310	5.60E-03
MEIS3	Meis homeobox 3	ENSG00000105419	-1.010	5.60E-03
RNPC3	RNA binding region (RNP1, RRM) containing 3	ENSG00000185946	-0.819	5.60E-03
SLC25A13	solute carrier family 25 member 13	ENSG00000004864	-0.494	5.60E-03
STX1A	syntaxin 1A	ENSG00000106089	-0.509	5.60E-03
TCF7	transcription factor 7	ENSG00000081059	0.989	5.60E-03
ZNF502	zinc finger protein 502	ENSG00000196653	1.883	5.60E-03
SHROOM2	shroom family member 2	ENSG00000146950	0.723	5.60E-04
MRPL45	mitochondrial ribosomal protein L45	ENSG00000278845	0.443	5.70E-03
RELL2	RELT like 2	ENSG00000164620	-0.698	5.70E-04
AGAP5 (includes others)	ArfGAP with GTPase domain, ankyrin repeat and PH domain 5	ENSG00000188234	1.134	5.80E-03
ARSG	arylsulfatase G	ENSG00000141337	-0.551	5.80E-03
CCDC91	coiled-coil domain containing 91	ENSG00000123106	-0.509	5.80E-03
LOC102606465	uncharacterized LOC102606465	ENSG00000233184	-1.003	5.80E-03
TBC1D12	TBC1 domain family member 12	ENSG00000108239	0.527	5.80E-03
SSBP2	single stranded DNA binding protein 2	ENSG00000145687	-0.595	5.90E-03
AC0057899		ENSG00000267090	2.490	5.90E-04
ST7-AS2	ST7 antisense RNA 2	ENSG00000226367	2.490	5.90E-04
COX11	cytochrome c oxidase copper chaperone COX11	ENSG00000166260	0.413	6.00E-03
RP11_110I16		ENSG00000255114	-0.378	6.00E-03
LOC101928525	uncharacterized LOC101928525	ENSG00000226706	0.566	6.10E-03
RP11_109D202		ENSG00000259352	-0.785	6.10E-03
TEX30	testis expressed 30	ENSG00000151287	0.728	6.10E-03
RP11_843B152		ENSG00000257530	1.326	6.20E-03
TAF4B	TATA-box binding protein associated factor 4b	ENSG00000141384	1.213	6.20E-03
VGLL3	vestigial like family member 3	ENSG00000206538	0.559	6.20E-04
CNIH3-AS2	CNIH3 antisense RNA 2	ENSG00000233384	-1.201	6.30E-03
PLXNB3	plexin B3	ENSG00000198753	0.483	6.30E-03
MRPL32	mitochondrial ribosomal protein L32	ENSG00000106591	0.438	6.40E-03
S100A13	S100 calcium binding protein A13	ENSG00000189171	-0.325	6.40E-03
ZNF337-AS1	ZNF337 antisense RNA 1	ENSG00000213742	-0.579	6.40E-03
RASSF8	Ras association domain family member 8	ENSG00000123094	0.381	6.50E-03
ROBO4	roundabout guidance receptor 4	ENSG00000154133	-2.762	6.50E-03
LINC02603	long intergenic non-protein coding RNA 2603	ENSG00000230262	-4.042	6.50E-05
CIB2	calcium and integrin binding family member 2	ENSG00000136425	-1.296	6.70E-04
KAT2B	lysine acetyltransferase 2B	ENSG00000114166	0.496	6.80E-03
ZBTB43	zinc finger and BTB domain containing 43	ENSG00000169155	0.563	6.80E-03
DIP2B	disco interacting protein 2 homolog B	ENSG00000066084	0.285	6.90E-03
CPD	carboxypeptidase D	ENSG00000108582	0.270	7.00E-03
FBXW9	F-box and WD repeat domain containing 9	ENSG00000132004	0.523	7.00E-03
MT-CYB	cytochrome b	ENSG00000198727	-0.211	7.00E-03
PEX16	peroxisomal biogenesis factor 16	ENSG00000121680	0.594	7.00E-03
ANO7	anoctamin 7	ENSG00000146205	-1.356	7.10E-03
MT-ATP8	ATP synthase F0 subunit 8	ENSG00000228253	-0.298	7.10E-03
TRIP6	thyroid hormone receptor interactor 6	ENSG00000087077	-0.320	7.10E-03
SCD	stearoyl-CoA desaturase	ENSG00000099194	0.219	7.20E-03
ZBTB3	zinc finger and BTB domain containing 3	ENSG00000185670	-1.068	7.20E-03

MAP4K3	mitogen-activated protein kinase kinase kinase kinase 3	ENSG00000011566	0.429	7.30E-03
TNFRSF10D	TNF receptor superfamily member 10d	ENSG00000173530	-0.387	7.30E-03
PPP3CA	protein phosphatase 3 catalytic subunit alpha	ENSG00000138814	0.273	7.40E-03
URI1	URI1 prefoldin like chaperone	ENSG00000105176	0.358	7.40E-03
LOC102723582	uncharacterized LOC102723582	ENSG00000272758	0.421	7.50E-03
CCDC151	coiled-coil domain containing 151	ENSG00000198003	-0.431	7.60E-03
CD2BP2-DT	CD2BP2 divergent transcript	ENSG00000260219	2.171	7.60E-03
CNBD2	cyclic nucleotide binding domain containing 2	ENSG00000149646	-1.585	7.60E-03
ESR1	estrogen receptor 1	ENSG00000091831	2.171	7.60E-03
FGF22	fibroblast growth factor 22	ENSG00000070388	3.252	7.60E-03
HNRNPLP2	heterogeneous nuclear ribonucleoprotein L pseudogene 2	ENSG00000259917	2.171	7.60E-03
LDHD	lactate dehydrogenase D	ENSG00000166816	3.252	7.60E-03
MORN2	MORN repeat containing 2	ENSG00000188010	-0.735	7.60E-03
PPP1R32	protein phosphatase 1 regulatory subunit 32	ENSG00000162148	3.252	7.60E-03
RPL34	ribosomal protein L34	ENSG00000109475	-0.240	7.60E-03
RP1_198K115		ENSG00000275457	0.701	7.70E-03
ZNF585B	zinc finger protein 585B	ENSG00000245680	0.701	7.70E-03
ABI3BP	ABI family member 3 binding protein	ENSG00000154175	-0.805	7.80E-03
JARID2	jumonji and AT-rich interaction domain containing 2	ENSG00000008083	0.374	7.80E-03
PPHLN1	periphilin 1	ENSG00000134283	-0.328	7.80E-03
ITGB1-DT	ITGB1 divergent transcript	ENSG00000229656	-2.188	7.90E-03
LHX1-DT	LHX1 divergent transcript	ENSG00000277268	-2.188	7.90E-03
LINC01504	long intergenic non-protein coding RNA 1504	ENSG00000225434	-2.188	7.90E-03
LOC105375519		ENSG00000273297	2.581	7.90E-03
MCTS2P	MCTS family member 2, pseudogene	ENSG00000101898	2.581	7.90E-03
RP11_197N182		ENSG00000256028	2.581	7.90E-03
SEMA4A	semaphorin 4A	ENSG00000196189	2.499	7.90E-03
TBC1D32	TBC1 domain family member 32	ENSG00000146350	-0.941	7.90E-03
CENPV	centromere protein V	ENSG00000166582	0.643	8.00E-03
RALB	RAS like proto-oncogene B	ENSG00000144118	-0.319	8.00E-03
ZNRF2	zinc and ring finger 2	ENSG00000180233	0.484	8.00E-03
COPS7B	COP9 signalosome subunit 7B	ENSG00000144524	-0.510	8.10E-04
RP11_159D122		ENSG00000264112	-0.768	8.20E-03
RUNX2	RUNX family transcription factor 2	ENSG00000124813	0.411	8.20E-03
C3orf33	chromosome 3 open reading frame 33	ENSG00000174928	1.460	8.30E-03
CES3	carboxylesterase 3	ENSG00000172828	1.081	8.30E-03
GDPGP1	GDP-D-glucose phosphorylase 1	ENSG00000183208	-0.365	8.30E-03
ZNF688	zinc finger protein 688	ENSG00000229809	0.546	8.30E-03
AC1425281		ENSG00000235078	-0.403	8.40E-03
CD82	CD82 molecule	ENSG00000085117	-0.251	8.40E-03
PIMREG	PICALM interacting mitotic regulator	ENSG00000129195	-0.362	8.40E-03
TTF1	transcription termination factor 1	ENSG00000125482	-0.508	8.40E-03
BDNF-AS	BDNF antisense RNA	ENSG00000245573	-0.752	8.40E-04
RPUSD4	RNA pseudouridine synthase D4	ENSG00000165526	-0.481	8.60E-03
TPTEP2	TPTE pseudogene 2	ENSG00000244627	1.956	8.60E-04
IQCD	IQ motif containing D	ENSG00000166578	1.829	8.70E-03

TRIM23	tripartite motif containing 23	ENSG00000113595	0.540	8.70E-03
SEC14L1	SEC14 like lipid binding 1	ENSG00000129657	0.278	8.80E-03
JADE3	jade family PHD finger 3	ENSG00000102221	-0.467	8.90E-03
RP11_205M53		ENSG00000280064	0.809	9.10E-03
ABCF3	ATP binding cassette subfamily F member 3	ENSG00000161204	-0.339	9.20E-03
GALNT18	polypeptide N-acetylgalactosaminyltransferase 18	ENSG00000110328	-0.541	9.20E-03
UTP14A	UTP14A small subunit processome component	ENSG00000156697	-0.439	9.30E-03
DPY19L4	dpy-19 like 4	ENSG00000156162	0.362	9.40E-03
MYO1D	myosin ID	ENSG00000176658	0.746	9.40E-03
LRFN4	leucine rich repeat and fibronectin type III domain containing 4	ENSG00000173621	0.445	9.40E-04
MROH8	maestro heat like repeat family member 8	ENSG00000101353	-0.346	9.50E-03
PRRT3	proline rich transmembrane protein 3	ENSG00000163704	0.640	9.50E-03
TP53I11	tumor protein p53 inducible protein 11	ENSG00000175274	0.357	9.50E-03
ZBTB39	zinc finger and BTB domain containing 39	ENSG00000166860	0.596	9.50E-03
BNC2	basonuclin 2	ENSG00000173068	0.382	9.60E-03
CWC27	CWC27 spliceosome associated cyclophilin	ENSG00000153015	-0.417	9.60E-03
LA16C_390E64		ENSG00000260051	1.527	9.70E-04
SNAP29	synaptosome associated protein 29	ENSG00000099940	0.449	9.80E-03
EDN1	endothelin 1	ENSG00000078401	1.114	9.90E-03
MRPL41	mitochondrial ribosomal protein L41	ENSG00000182154	-0.309	9.90E-03
SMG1P2	SMG1 pseudogene 2	ENSG00000205534	-1.598	9.90E-03
CRACR2B	calcium release activated channel regulator 2B	ENSG00000177685	2.432	9.90E-04

Table S2. List of DEGs in FSK treatment (FC = 0, adjusted *p*-value ≤ 0.05).

Symbol	*Entrez Gene Name*	*ENSEMBL*	*Expr Log Ratio*	*Expr p-Value*
A4GALT	alpha 1,4-galactosyltransferase (P blood group)	ENSG00000128274	1.561	7.10E-04
AASS	aminoadipate-semialdehyde synthase	ENSG00000008311	0.942	3.40E-02
ABCA13	ATP binding cassette subfamily A member 13	ENSG00000179869	2.148	3.90E-03
ABCA8	ATP binding cassette subfamily A member 8	ENSG00000141338	1.503	7.20E-04
ABCC4	ATP binding cassette subfamily C member 4	ENSG00000125257	1.291	1.40E-03
ABL1	ABL proto-oncogene 1, non-receptor tyrosine kinase	ENSG00000097007	0.778	4.90E-02
AC0053392		ENSG00000268565	-1.170	3.30E-02
AC0075635		ENSG00000236886	1.080	6.40E-03
AC0107616		ENSG00000265073	2.034	1.60E-02
AC0374451		ENSG00000233635	1.696	1.50E-02
AC0695134		ENSG00000229178	-3.453	2.00E-02
AC1147308		ENSG00000215692	1.008	4.50E-02
ACBD4	acyl-CoA binding domain containing 4	ENSG00000181513	0.962	3.70E-02
ACE	angiotensin I converting enzyme	ENSG00000159640	2.004	4.20E-02
ACHE	acetylcholinesterase (Cartwright blood group)	ENSG00000087085	-3.603	1.30E-02
ACTG2	actin gamma 2, smooth muscle	ENSG00000163017	-1.011	2.20E-02
ADAMTS15	ADAM metallopeptidase with thrombospondin type 1 motif 15	ENSG00000166106	0.964	1.90E-02
ADAMTS7	ADAM metallopeptidase with thrombospondin type 1 motif 7	ENSG00000136378	-0.873	2.90E-02
ADAMTSL3	ADAMTS like 3	ENSG00000156218	1.743	1.80E-02
ADCY1	adenylate cyclase 1	ENSG00000164742	-2.520	7.10E-03
ADCY3	adenylate cyclase 3	ENSG00000138031	-0.976	1.80E-02
ADGRG1	adhesion G protein-coupled receptor G1	ENSG00000205336	-1.135	1.40E-02
ADSS1	adenylosuccinate synthase 1	ENSG00000185100	3.315	4.80E-03
AFAP1L2	actin filament associated protein 1 like 2	ENSG00000169129	-1.354	1.60E-02
AFF3	AF4/FMR2 family member 3	ENSG00000144218	-1.598	7.70E-03
AHI1	Abelson helper integration site 1	ENSG00000135541	1.261	2.60E-03
ALDH1A1	aldehyde dehydrogenase 1 family member A1	ENSG00000165092	1.503	1.30E-02
ALDH1A3	aldehyde dehydrogenase 1 family member A3	ENSG00000184254	-1.076	9.70E-03
ALDH1L2	aldehyde dehydrogenase 1 family member L2	ENSG00000136010	-0.889	3.70E-02
ALDH3B1	aldehyde dehydrogenase 3 family member B1	ENSG00000006534	0.968	1.80E-02
ALPL	alkaline phosphatase, biomineralization associated	ENSG00000162551	2.153	8.50E-03
AMDHD1	amidohydrolase domain containing 1	ENSG00000139344	2.737	3.30E-02
AMPD3	adenosine monophosphate deaminase 3	ENSG00000133805	-0.992	2.60E-02
ANGPT1	angiopoietin 1	ENSG00000154188	1.770	1.10E-04
ANGPTL2	angiopoietin like 2	ENSG00000136859	1.085	1.30E-02
ANGPTL4	angiopoietin like 4	ENSG00000167772	-1.605	4.30E-04

ANKRD34A	ankyrin repeat domain 34A	ENSG00000272031	1.842	1.00E-02
ANLN	anillin actin binding protein	ENSG00000011426	-1.582	9.20E-05
ANO7	anoctamin 7	ENSG00000146205	-3.060	1.20E-04
ANTXR1	ANTXR cell adhesion molecule 1	ENSG00000169604	1.620	5.00E-05
AP1S3	adaptor related protein complex 1 subunit sigma 3	ENSG00000152056	1.043	2.30E-02
AP3M2	adaptor related protein complex 3 subunit mu 2	ENSG00000070718	-0.938	3.30E-02
APCDD1L	APC down-regulated 1 like	ENSG00000198768	-1.692	2.00E-04
APCDD1L-DT	APCDD1L divergent transcript	ENSG00000231290	-1.518	3.10E-02
APOA1	apolipoprotein A1	ENSG00000118137	-2.698	1.30E-02
APOE	apolipoprotein E	ENSG00000130203	-1.521	3.00E-03
AQP2	aquaporin 2	ENSG00000167580	7.381	1.60E-05
ARHGAP11A	Rho GTPase activating protein 11A	ENSG00000198826	-1.212	3.40E-03
ARHGAP19	Rho GTPase activating protein 19	ENSG00000213390	-1.044	3.50E-02
ARHGDIB	Rho GDP dissociation inhibitor beta	ENSG00000111348	-1.197	6.70E-03
ARHGEF19	Rho guanine nucleotide exchange factor 19	ENSG00000142632	0.905	2.70E-02
ARHGEF19-AS1	ARHGEF19 antisense RNA 1	ENSG00000234166	1.362	1.20E-02
ARHGEF34P	Rho guanine nucleotide exchange factor 34, pseudogene	ENSG00000204959	1.487	2.90E-02
ARNTL2-AS1	ARNTL2 antisense RNA 1	ENSG00000245311	-1.400	3.60E-02
ARRDC3	arrestin domain containing 3	ENSG00000113369	1.275	1.90E-03
ARSB	arylsulfatase B	ENSG00000113273	0.797	5.00E-02
ARSD-AS1	ARSD antisense RNA 1	ENSG00000229851	0.870	4.20E-02
ASF1B	anti-silencing function 1B histone chaperone	ENSG00000105011	-1.570	1.70E-04
ASNS	asparagine synthetase (glutamine-hydrolyzing)	ENSG00000070669	-1.018	1.70E-02
ASPM	abnormal spindle microtubule assembly	ENSG00000066279	-1.467	3.20E-04
ASRGL1	asparaginase and isoaspartyl peptidase 1	ENSG00000162174	-1.239	4.00E-02
ATAD2	ATPase family AAA domain containing 2	ENSG00000156802	-1.056	1.10E-02
ATP23	ATP23 metallopeptidase and ATP synthase assembly factor homolog	ENSG00000166896	-1.883	4.10E-02
ATP2A1-AS1	ATP2A1 antisense RNA 1	ENSG00000260442	-2.015	2.10E-02
ATP8B4	ATPase phospholipid transporting 8B4 (putative)	ENSG00000104043	3.330	4.80E-04
AURKA	aurora kinase A	ENSG00000087586	-1.284	2.20E-03
AURKB	aurora kinase B	ENSG00000178999	-1.592	2.50E-04
AVPI1	arginine vasopressin induced 1	ENSG00000119986	2.031	4.70E-06
BAG1	BCL2 associated athanogene 1	ENSG00000107262	0.851	3.50E-02
BARD1	BRCA1 associated RING domain 1	ENSG00000138376	-1.307	5.70E-03
BCAR3	BCAR3 adaptor protein, NSP family member	ENSG00000137936	-0.802	4.90E-02
BCL2	BCL2 apoptosis regulator	ENSG00000171791	-2.168	8.10E-04
BCL2L11	BCL2 like 11	ENSG00000153094	0.901	3.20E-02
BCYRN1	brain cytoplasmic RNA 1	ENSG00000236824	-1.329	2.60E-03
BDNF	brain derived neurotrophic factor	ENSG00000176697	1.342	1.10E-03
BDNF-AS	BDNF antisense RNA	ENSG00000245573	0.860	4.60E-02
BEX2	brain expressed X-linked 2	ENSG00000133134	2.280	2.20E-03
BIRC5	baculoviral IAP repeat containing 5	ENSG00000089685	-1.547	1.70E-04
BLM	BLM RecQ like helicase	ENSG00000197299	-1.457	4.50E-03
BMP3	bone morphogenetic protein 3	ENSG00000152785	2.564	1.90E-02

BOLA3-AS1	BOLA3 divergent transcript	ENSG00000225439	1.571	2.40E-02
BRCA1	BRCA1 DNA repair associated	ENSG00000012048	-1.447	8.10E-04
BRIP1	BRCA1 interacting protein C-terminal helicase 1	ENSG00000136492	-1.107	1.40E-02
BUB1	BUB1 mitotic checkpoint serine/threonine kinase	ENSG00000169679	-1.080	8.40E-03
BUB1B	BUB1 mitotic checkpoint serine/threonine kinase B	ENSG00000156970	-1.945	7.40E-06
C10orf25	chromosome 10 open reading frame 25	ENSG00000165511	3.407	3.00E-03
C10orf55	chromosome 10 open reading frame 55	ENSG00000222047	-1.059	8.60E-03
C11orf71	chromosome 11 open reading frame 71	ENSG00000180425	1.696	1.50E-02
C11orf96	chromosome 11 open reading frame 96	ENSG00000187479	3.029	1.30E-10
C12orf60	chromosome 12 open reading frame 60	ENSG00000182993	-0.789	5.00E-02
C17orf107	chromosome 17 open reading frame 107	ENSG00000205710	2.873	2.00E-02
C17orf53	chromosome 17 open reading frame 53	ENSG00000125319	-1.385	1.30E-02
C1QL4	complement C1q like 4	ENSG00000186897	-1.759	4.20E-02
C1QTNF7	C1q and TNF related 7	ENSG00000163145	1.151	2.00E-02
C1RL	complement C1r subcomponent like	ENSG00000139178	0.895	2.80E-02
C2orf81	chromosome 2 open reading frame 81	ENSG00000159239	1.136	2.20E-02
C4orf46	chromosome 4 open reading frame 46	ENSG00000205208	-1.282	4.70E-03
C7orf57	chromosome 7 open reading frame 57	ENSG00000164746	1.903	3.10E-02
C9orf131	chromosome 9 open reading frame 131	ENSG00000174038	2.737	3.30E-02
CA9	carbonic anhydrase 9	ENSG00000107159	2.079	1.40E-03
CABLES1	Cdk5 and Abl enzyme substrate 1	ENSG00000134508	1.071	1.30E-02
CACNA2D2	calcium voltage-gated channel auxiliary subunit alpha2delta 2	ENSG00000007402	3.576	4.70E-03
CADM1	cell adhesion molecule 1	ENSG00000182985	1.461	4.60E-04
CALB2	calbindin 2	ENSG00000172137	2.034	2.00E-06
CARD10	caspase recruitment domain family member 10	ENSG00000100065	-1.129	1.00E-02
CARD11	caspase recruitment domain family member 11	ENSG00000198286	-1.068	1.10E-02
CARD16	caspase recruitment domain family member 16	ENSG00000204397	0.808	4.80E-02
CARNS1	carnosine synthase 1	ENSG00000172508	3.087	1.30E-09
CASC2	cancer susceptibility 2	ENSG00000177640	1.211	4.30E-02
CBR3-AS1	CBR3 antisense RNA 1	ENSG00000236830	-0.925	4.40E-02
CC2D2B	coiled-coil and C2 domain containing 2B	ENSG00000188649	-2.008	2.80E-02
CCBE1	collagen and calcium binding EGF domains 1	ENSG00000183287	-1.130	3.30E-02
CCDC114	coiled-coil domain containing 114	ENSG00000105479	1.487	2.90E-02
CCDC120	coiled-coil domain containing 120	ENSG00000147144	1.081	2.70E-02
CCDC138	coiled-coil domain containing 138	ENSG00000163006	-1.519	5.70E-03
CCDC34	coiled-coil domain containing 34	ENSG00000109881	-0.910	4.10E-02
CCDC85B	coiled-coil domain containing 85B	ENSG00000175602	-0.808	4.10E-02
CCN3	cellular communication network factor 3	ENSG00000136999	1.415	1.00E-02
CCNA2	cyclin A2	ENSG00000145386	-0.939	2.10E-02
CCNB1	cyclin B1	ENSG00000134057	-1.599	1.00E-04
CCNB2	cyclin B2	ENSG00000157456	-1.478	5.80E-04
CCNE1	cyclin E1	ENSG00000105173	-1.010	3.10E-02
CCNF	cyclin F	ENSG00000162063	-1.106	8.30E-03
CCP110	centriolar coiled-coil protein 110	ENSG00000103540	-0.833	5.00E-02
CD200	CD200 molecule	ENSG00000091972	1.146	4.80E-03

CD2BP2-DT	CD2BP2 divergent transcript	ENSG00000260219	2.163	2.10E-02
CD55	CD55 molecule (Cromer blood group)	ENSG00000196352	1.707	2.20E-05
CD74	CD74 molecule	ENSG00000019582	1.101	5.90E-03
CD9	CD9 molecule	ENSG00000010278	-1.010	1.10E-02
CDC20	cell division cycle 20	ENSG00000117399	-1.696	4.10E-05
CDC42EP2	CDC42 effector protein 2	ENSG00000149798	-1.030	2.10E-02
CDC45	cell division cycle 45	ENSG00000093009	-2.088	1.10E-05
CDC6	cell division cycle 6	ENSG00000094804	-1.370	1.40E-03
CDCA2	cell division cycle associated 2	ENSG00000184661	-1.749	1.00E-04
CDCA3	cell division cycle associated 3	ENSG00000111665	-0.969	2.60E-02
CDCA4	cell division cycle associated 4	ENSG00000170779	-1.355	1.30E-03
CDCA5	cell division cycle associated 5	ENSG00000146670	-1.298	1.90E-03
CDCA7	cell division cycle associated 7	ENSG00000144354	-1.249	8.20E-03
CDCA8	cell division cycle associated 8	ENSG00000134690	-1.752	8.30E-05
CDH11	cadherin 11	ENSG00000140937	1.014	1.00E-02
CDK1	cyclin dependent kinase 1	ENSG00000170312	-1.310	1.50E-03
CDKN2C	cyclin dependent kinase inhibitor 2C	ENSG00000123080	-1.196	4.20E-03
CDKN3	cyclin dependent kinase inhibitor 3	ENSG00000100526	-1.518	6.60E-04
CDT1	chromatin licensing and DNA replication factor 1	ENSG00000167513	-1.011	2.10E-02
CEBPD	CCAAT enhancer binding protein delta	ENSG00000221869	1.583	1.20E-04
CELF2	CUGBP Elav-like family member 2	ENSG00000048740	1.075	3.00E-02
CENPA	centromere protein A	ENSG00000115163	-1.621	7.00E-04
CENPE	centromere protein E	ENSG00000138778	-1.470	5.20E-04
CENPF	centromere protein F	ENSG00000117724	-1.674	3.90E-05
CENPH	centromere protein H	ENSG00000153044	-0.982	3.70E-02
CENPI	centromere protein I	ENSG00000102384	-1.772	2.80E-04
CENPJ	centromere protein J	ENSG00000151849	-1.096	1.70E-02
CENPK	centromere protein K	ENSG00000123219	-1.651	4.40E-04
CENPL	centromere protein L	ENSG00000120334	-1.124	1.50E-02
CENPM	centromere protein M	ENSG00000100162	-1.123	2.10E-02
CENPU	centromere protein U	ENSG00000151725	-1.425	1.00E-03
CENPW	centromere protein W	ENSG00000203760	-1.934	6.90E-05
CENPX	centromere protein X	ENSG00000169689	-1.203	4.30E-03
CEP128	centrosomal protein 128	ENSG00000100629	-1.610	2.90E-03
CEP152	centrosomal protein 152	ENSG00000103995	-1.087	2.70E-02
CEP55	centrosomal protein 55	ENSG00000138180	-1.735	5.10E-05
CEP78	centrosomal protein 78	ENSG00000148019	-0.946	2.50E-02
CEP83	centrosomal protein 83	ENSG00000173588	-1.264	2.10E-02
CEP85	centrosomal protein 85	ENSG00000130695	-0.867	4.50E-02
CFH	complement factor H	ENSG00000000971	1.328	1.10E-03
CHAC1	ChaC glutathione specific gamma-glutamylcyclotransferase 1	ENSG00000128965	-1.644	6.00E-04
CHAF1A	chromatin assembly factor 1 subunit A	ENSG00000167670	-0.982	1.70E-02
CHGB	chromogranin B	ENSG00000089199	1.673	6.10E-03
CHL1	cell adhesion molecule L1 like	ENSG00000134121	1.896	7.20E-06
CHL1-AS1	CHL1 antisense RNA 1	ENSG00000234661	1.425	1.60E-02
CHL1-AS2	CHL1 antisense RNA 2	ENSG00000224318	1.971	2.00E-03
CHMP1B	charged multivesicular body protein 1B	ENSG00000255112	0.814	4.40E-02
CHRM2	cholinergic receptor muscarinic 2	ENSG00000181072	1.761	4.20E-03
CHST1	carbohydrate sulfotransferase 1	ENSG00000175264	-1.611	4.10E-04
CHTF18	chromosome transmission fidelity factor 18	ENSG00000127586	-0.904	2.70E-02

CIB2	calcium and integrin binding family member 2	ENSG00000136425	-1.902	1.20E-03
CIP2A	cell proliferation regulating inhibitor of protein phosphatase 2A	ENSG00000163507	-1.388	1.60E-03
CIT	citron rho-interacting serine/threonine kinase	ENSG00000122966	-1.538	2.80E-04
CKAP2	cytoskeleton associated protein 2	ENSG00000136108	-1.009	1.40E-02
CKAP2L	cytoskeleton associated protein 2 like	ENSG00000169607	-1.766	4.80E-05
CKS1B	CDC28 protein kinase regulatory subunit 1B	ENSG00000173207	-1.290	2.00E-03
CKS2	CDC28 protein kinase regulatory subunit 2	ENSG00000123975	-1.418	6.60E-04
CLSPN	claspin	ENSG00000092853	-1.244	4.30E-03
CMSS1	cms1 ribosomal small subunit homolog	ENSG00000184220	-0.920	3.60E-02
CNBD2	cyclic nucleotide binding domain containing 2	ENSG00000149646	-1.383	4.90E-02
CNIH3-AS2	CNIH3 antisense RNA 2	ENSG00000233384	-1.453	1.50E-02
CNTN5	contactin 5	ENSG00000149972	1.104	1.70E-02
CNTRL	centriolin	ENSG00000119397	-1.019	2.30E-02
COL13A1	collagen type XIII alpha 1 chain	ENSG00000197467	-1.838	6.10E-04
COL4A5	collagen type IV alpha 5 chain	ENSG00000188153	0.971	1.60E-02
COL4A6	collagen type IV alpha 6 chain	ENSG00000197565	1.181	4.70E-03
COL6A3	collagen type VI alpha 3 chain	ENSG00000163359	-1.444	6.60E-04
COL8A2	collagen type VIII alpha 2 chain	ENSG00000171812	-1.851	3.10E-02
CORO2B	coronin 2B	ENSG00000103647	-1.438	1.40E-03
CPE	carboxypeptidase E	ENSG00000109472	0.941	1.70E-02
CPEB4	cytoplasmic polyadenylation element binding protein 4	ENSG00000113742	1.651	6.30E-05
CRACR2B	calcium release activated channel regulator 2B	ENSG00000177685	2.004	4.20E-02
CREG1	cellular repressor of E1A stimulated genes 1	ENSG00000143162	0.992	1.60E-02
CRISPLD2	cysteine rich secretory protein LCCL domain containing 2	ENSG00000103196	1.096	2.20E-02
CRYBB2P1	crystallin beta B2 pseudogene 1	ENSG00000100058	-1.109	3.60E-02
CSGALNACT1	chondroitin sulfate N-acetylgalactosaminyltransferase 1	ENSG00000147408	2.703	3.30E-08
CSPG4	chondroitin sulfate proteoglycan 4	ENSG00000173546	-1.327	4.40E-03
CSRP2	cysteine and glycine rich protein 2	ENSG00000175183	-1.099	1.60E-02
CTA_243E71		ENSG00000279110	-1.759	4.20E-02
CTB_193M125		ENSG00000280206	-1.322	1.20E-02
CTB_58E171		ENSG00000277969	1.716	1.80E-02
CTC_453G235		ENSG00000269534	-1.367	8.10E-03
CTC_463A161		ENSG00000280047	2.997	2.00E-02
CTD-2201I18.1	uncharacterized LOC101929215	ENSG00000249825	6.130	6.90E-03
CTD_2006M222		ENSG00000278934	1.834	4.20E-02
CTD_2017F171		ENSG00000275332	2.478	2.80E-02
CTD_2105E1314		ENSG00000268729	2.264	6.20E-03
CTD_2196E143		ENSG00000261723	-1.721	3.10E-02
CTD_2196E145		ENSG00000261266	-1.808	1.50E-04
CTD_2228K21		ENSG00000250645	-2.145	3.90E-02
CTD_2311M213		ENSG00000261821	2.101	4.30E-06
CTD_2510F54		ENSG00000265415	-1.248	2.40E-02

CTD_2544N143		ENSG00000253645	1.685	2.50E-04
CTD_3203P23		ENSG00000274092	1.201	2.50E-02
CTNNA2	catenin alpha 2	ENSG00000066032	2.362	1.20E-03
CTSW	cathepsin W	ENSG00000172543	-1.916	3.00E-02
CYP11A1	cytochrome P450 family 11 subfamily A member 1	ENSG00000140459	2.252	8.50E-08
CYP19A1	cytochrome P450 family 19 subfamily A member 1	ENSG00000137869	3.196	3.50E-13
DACT1	dishevelled binding antagonist of beta catenin 1	ENSG00000165617	0.804	4.50E-02
DAW1	dynein assembly factor with WD repeats 1	ENSG00000123977	4.260	1.30E-04
DBF4	DBF4 zinc finger	ENSG00000006634	-1.245	1.30E-02
DBF4B	DBF4 zinc finger B	ENSG00000161692	-1.085	2.10E-02
DCLK1	doublecortin like kinase 1	ENSG00000133083	1.036	3.00E-02
DCLRE1B	DNA cross-link repair 1B	ENSG00000118655	-0.984	3.10E-02
DCN	decorin	ENSG00000011465	2.797	9.00E-03
DDIAS	DNA damage induced apoptosis suppressor	ENSG00000165490	-1.373	5.70E-03
DDX11	DEAD/H-box helicase 11	ENSG00000013573	-1.158	6.80E-03
DEK	DEK proto-oncogene	ENSG00000124795	-0.807	4.20E-02
DENND5B	DENN domain containing 5B	ENSG00000170456	1.058	1.20E-02
DEPDC1	DEP domain containing 1	ENSG00000024526	-1.012	1.60E-02
DEPDC1B	DEP domain containing 1B	ENSG00000035499	-1.627	1.30E-03
DEPTOR	DEP domain containing MTOR interacting protein	ENSG00000155792	1.345	2.00E-03
DHCR24	24-dehydrocholesterol reductase	ENSG00000116133	0.957	1.50E-02
DIAPH3	diaphanous related formin 3	ENSG00000139734	-1.107	7.90E-03
DIAPH3-AS1	DIAPH3 antisense RNA 1	ENSG00000227528	-1.835	9.40E-03
DIO2	iodothyronine deiodinase 2	ENSG00000211448	2.308	2.70E-06
DKFZP434P228		ENSG00000279232	-0.905	3.00E-02
DLGAP1	DLG associated protein 1	ENSG00000170579	0.892	5.00E-02
DLGAP1-AS4	DLGAP1 antisense RNA 4	ENSG00000263878	6.969	1.70E-04
DLGAP5	DLG associated protein 5	ENSG00000126787	-1.608	1.50E-04
DLSTP1	dihydrolipoamide S-succinyltransferase pseudogene 1	ENSG00000181227	6.390	2.30E-03
DMBT1	deleted in malignant brain tumors 1	ENSG00000187908	2.327	1.20E-05
DNA2	DNA replication helicase/nuclease 2	ENSG00000138346	-1.082	2.30E-02
DNAJC9	DnaJ heat shock protein family (Hsp40) member C9	ENSG00000213551	-1.029	1.20E-02
DNER	delta/notch like EGF repeat containing	ENSG00000187957	3.217	7.80E-03
DNMT3B	DNA methyltransferase 3 beta	ENSG00000088305	-1.235	8.00E-03
DOK3	docking protein 3	ENSG00000146094	-1.424	9.60E-03
DPF1	double PHD fingers 1	ENSG00000011332	-1.428	8.60E-03
DPY19L3	dpy-19 like C-mannosyltransferase 3	ENSG00000178904	0.831	4.70E-02
DRAXIN	dorsal inhibitory axon guidance protein	ENSG00000162490	-2.905	1.40E-03
DSN1	DSN1 component of MIS12 kinetochore complex	ENSG00000149636	-1.244	4.70E-03
DTL	denticleless E3 ubiquitin protein ligase homolog	ENSG00000143476	-1.209	4.60E-03
DTWD1	DTW domain containing 1	ENSG00000104047	1.145	5.20E-03
DTYMK	deoxythymidylate kinase	ENSG00000168393	-1.142	5.30E-03
DUSP1	dual specificity phosphatase 1	ENSG00000120129	1.230	2.10E-03
DUSP5	dual specificity phosphatase 5	ENSG00000138166	-1.716	4.80E-04

DUSP8P5	dual specificity phosphatase 8 pseudogene 5	ENSG00000235316	1.377	3.60E-02
E2F1	E2F transcription factor 1	ENSG00000101412	-0.878	3.40E-02
E2F7	E2F transcription factor 7	ENSG00000165891	-0.920	2.10E-02
E2F8	E2F transcription factor 8	ENSG00000129173	-1.229	2.50E-02
ECT2	epithelial cell transforming 2	ENSG00000114346	-0.849	3.60E-02
EDN1	endothelin 1	ENSG00000078401	1.834	9.00E-04
EEF1G	eukaryotic translation elongation factor 1 gamma	ENSG00000254772	-2.145	3.90E-02
EFCAB14-AS1	EFCAB14 antisense RNA 1	ENSG00000228237	1.206	3.50E-02
EFCAB2	EF-hand calcium binding domain 2	ENSG00000203666	1.070	3.90E-02
EFEMP1	EGF containing fibulin extracellular matrix protein 1	ENSG00000115380	1.128	4.30E-03
EFHD2	EF-hand domain family member D2	ENSG00000142634	-0.984	1.50E-02
EGFL8	EGF like domain multiple 8	ENSG00000241404	1.719	3.10E-02
EGR2	early growth response 2	ENSG00000122877	-1.225	4.10E-02
EGR3	early growth response 3	ENSG00000179388	-1.059	1.20E-02
EMC3-AS1	EMC3 antisense RNA 1	ENSG00000180385	-1.229	2.50E-02
EMP2	epithelial membrane protein 2	ENSG00000213853	-0.866	4.40E-02
ENOSF1	enolase superfamily member 1	ENSG00000132199	-0.987	1.60E-02
EPHA5	EPH receptor A5	ENSG00000145242	1.505	1.20E-03
EPHA5-AS1	EPHA5 antisense RNA 1	ENSG00000250846	1.337	4.40E-02
EPHB2	EPH receptor B2	ENSG00000133216	-0.867	3.00E-02
EPPK1	epiplakin 1	ENSG00000261150	6.266	3.90E-03
ERCC6L	ERCC excision repair 6 like, spindle assembly checkpoint helicase	ENSG00000186871	-1.326	7.70E-03
ERFE	erythroferrone	ENSG00000178752	-1.851	3.10E-02
ERI1	exoribonuclease 1	ENSG00000104626	-1.114	1.30E-02
ERICH3	glutamate rich 3	ENSG00000178965	-4.084	1.20E-03
ERN2	endoplasmic reticulum to nucleus signaling 2	ENSG00000134398	-2.008	2.80E-02
ESCO2	establishment of sister chromatid cohesion N-acetyltransferase 2	ENSG00000171320	-2.029	3.20E-05
ESPL1	extra spindle pole bodies like 1, separase	ENSG00000135476	-1.550	3.00E-04
ETS1	ETS proto-oncogene 1, transcription factor	ENSG00000134954	-0.952	2.10E-02
EXO1	exonuclease 1	ENSG00000174371	-1.480	1.00E-03
EXOSC8	exosome component 8	ENSG00000120699	-0.870	4.40E-02
EZH2	enhancer of zeste 2 polycomb repressive complex 2 subunit	ENSG00000106462	-0.959	2.40E-02
FA2H	fatty acid 2-hydroxylase	ENSG00000103089	2.037	3.30E-03
FAHD2CP	fumarylacetoacetate hydrolase domain containing 2C, pseudogene	ENSG00000231584	-1.186	2.30E-02
FAIM2	Fas apoptotic inhibitory molecule 2	ENSG00000135472	6.266	3.90E-03
FAM110A	family with sequence similarity 110 member A	ENSG00000125898	-1.259	6.50E-03
FAM166A	family with sequence similarity 166 member A	ENSG00000188163	-1.066	1.40E-02
FAM72C/FAM72D	family with sequence similarity 72 member D	ENSG00000263513	-3.603	1.30E-02
FAM83D	family with sequence similarity 83 member D	ENSG00000101447	-1.830	2.20E-05
FANCA	FA complementation group A	ENSG00000187741	-0.939	2.30E-02
FANCD2	FA complementation group D2	ENSG00000144554	-1.145	8.80E-03

FANCG	FA complementation group G	ENSG00000221829	-0.888	3.60E-02
FANCI	FA complementation group I	ENSG00000140525	-0.991	1.50E-02
FANCM	FA complementation group M	ENSG00000187790	-1.141	2.80E-02
FAT1	FAT atypical cadherin 1	ENSG00000083857	1.250	1.60E-03
FBXO5	F-box protein 5	ENSG00000112029	-1.449	1.30E-03
FCMR	Fc fragment of IgM receptor	ENSG00000162894	2.477	5.10E-04
FEN1	flap structure-specific endonuclease 1	ENSG00000168496	-1.169	4.40E-03
FEZF1	FEZ family zinc finger 1	ENSG00000128610	1.258	4.60E-02
FEZF1-AS1	FEZF1 antisense RNA 1	ENSG00000230316	2.038	7.80E-04
FGF1	fibroblast growth factor 1	ENSG00000113578	-1.644	6.10E-03
FGF5	fibroblast growth factor 5	ENSG00000138675	-2.196	1.20E-03
FGF7	fibroblast growth factor 7	ENSG00000140285	2.945	3.80E-05
FHDC1	FH2 domain containing 1	ENSG00000137460	2.564	1.90E-02
FLNC	filamin C	ENSG00000128591	-0.921	2.00E-02
FLRT3	fibronectin leucine rich transmembrane protein 3	ENSG00000125848	2.000	1.60E-03
FNDC1	fibronectin type III domain containing 1	ENSG00000164694	-1.509	2.40E-02
FOSL1	FOS like 1, AP-1 transcription factor subunit	ENSG00000175592	-1.004	1.10E-02
FOXF2	forkhead box F2	ENSG00000137273	1.554	9.80E-04
FOXG1	forkhead box G1	ENSG00000176165	-0.834	4.50E-02
FOXM1	forkhead box M1	ENSG00000111206	-1.262	1.90E-03
FOXO1	forkhead box O1	ENSG00000150907	1.412	1.60E-03
FOXS1	forkhead box S1	ENSG00000179772	-1.981	1.70E-04
FSHR	follicle stimulating hormone receptor	ENSG00000170820	3.080	1.90E-04
FUT10	fucosyltransferase 10	ENSG00000172728	0.935	4.60E-02
GAB2	GRB2 associated binding protein 2	ENSG00000033327	0.925	2.20E-02
GADD45A	growth arrest and DNA damage inducible alpha	ENSG00000116717	-0.783	5.00E-02
GALNT1	polypeptide N-acetylgalactosaminyltransferase 1	ENSG00000141429	1.488	1.90E-04
GALNT15	polypeptide N-acetylgalactosaminyltransferase 15	ENSG00000131386	6.887	2.80E-04
GAREM1	GRB2 associated regulator of MAPK1 subtype 1	ENSG00000141441	0.930	3.10E-02
GAS1	growth arrest specific 1	ENSG00000180447	1.969	7.80E-05
GAS2L3	growth arrest specific 2 like 3	ENSG00000139354	-1.336	5.80E-03
GATA5	GATA binding protein 5	ENSG00000130700	1.186	8.80E-03
GATA6-AS1	GATA6 antisense RNA 1 (head to head)	ENSG00000266010	1.174	3.40E-02
GBP4	guanylate binding protein 4	ENSG00000162654	0.855	3.20E-02
GEM	GTP binding protein overexpressed in skeletal muscle	ENSG00000164949	-1.556	3.50E-04
GFPT2	glutamine-fructose-6-phosphate transaminase 2	ENSG00000131459	-1.328	2.00E-03
GINS1	GINS complex subunit 1	ENSG00000101003	-1.076	1.60E-02
GINS2	GINS complex subunit 2	ENSG00000131153	-0.930	4.10E-02
GINS4	GINS complex subunit 4	ENSG00000147536	-1.820	5.90E-05
GLP2R	glucagon like peptide 2 receptor	ENSG00000065325	2.997	2.00E-02
GLRX	glutaredoxin	ENSG00000173221	0.842	3.70E-02
GMNN	geminin DNA replication inhibitor	ENSG00000112312	-1.018	1.80E-02
GNAI1	G protein subunit alpha i1	ENSG00000127955	1.315	1.50E-03
GNB3	G protein subunit beta 3	ENSG00000111664	-1.691	1.70E-02
GNPTAB	N-acetylglucosamine-1-phosphate trans-	ENSG00000111670	1.212	2.50E-03

	ferase subunits alpha and beta			
GOLGA8J (includes others)	golgin A8 family member K	ENSG00000232653	2.385	4.10E-02
GON7	GON7 subunit of KEOPS complex	ENSG00000170270	-1.395	1.10E-02
GPC4	glypican 4	ENSG00000076716	1.168	4.10E-03
GPC6	glypican 6	ENSG00000183098	1.002	1.20E-02
GPR135	G protein-coupled receptor 135	ENSG00000181619	1.140	3.20E-02
GPR68	G protein-coupled receptor 68	ENSG00000119714	-1.447	4.10E-02
GPR85	G protein-coupled receptor 85	ENSG00000164604	1.193	3.20E-02
GPRASP1	G protein-coupled receptor associated sorting protein 1	ENSG00000198932	1.526	3.00E-02
GPRC5A	G protein-coupled receptor class C group 5 member A	ENSG00000013588	1.518	4.40E-04
GPRIN1	G protein regulated inducer of neurite outgrowth 1	ENSG00000169258	-0.953	4.80E-02
GRAMD1B	GRAM domain containing 1B	ENSG00000023171	1.670	7.90E-04
GREM1	gremlin 1, DAN family BMP antagonist	ENSG00000166923	1.625	4.10E-03
GRIA4	glutamate ionotropic receptor AMPA type subunit 4	ENSG00000152578	2.660	1.10E-04
GRK3	G protein-coupled receptor kinase 3	ENSG00000100077	-1.449	3.90E-02
GTSE1	G2 and S-phase expressed 1	ENSG00000075218	-1.850	2.20E-05
H2AC11	H2A clustered histone 11	ENSG00000196787	-2.122	1.90E-02
H2AX	H2A.X variant histone	ENSG00000188486	-1.106	5.40E-03
H2AZ1	H2A.Z variant histone 1	ENSG00000164032	-1.111	5.20E-03
H2AZ1-DT	H2AZ1 divergent transcript	ENSG00000245322	-1.293	1.40E-03
H3C6	H3 clustered histone 6	ENSG00000274750	1.300	1.80E-02
HASPIN	histone H3 associated protein kinase	ENSG00000177602	-1.056	3.60E-02
HAUS8	HAUS augmin like complex subunit 8	ENSG00000131351	-1.291	5.80E-03
HEG1	heart development protein with EGF like domains 1	ENSG00000173706	1.556	9.70E-05
HES1	hes family bHLH transcription factor 1	ENSG00000114315	-1.392	2.30E-03
HEY1	hes related family bHLH transcription factor with YRPW motif 1	ENSG00000164683	-1.509	2.40E-02
HHAT	hedgehog acyltransferase	ENSG00000054392	1.092	2.60E-02
HIP1	huntingtin interacting protein 1	ENSG00000127946	-0.831	4.00E-02
HJURP	Holliday junction recognition protein	ENSG00000123485	-1.722	5.90E-05
HLA-DMA	major histocompatibility complex, class II, DM alpha	ENSG00000204257	1.217	2.20E-02
HLA-DMB	major histocompatibility complex, class II, DM beta	ENSG00000242574	2.299	6.00E-04
HLA-DOA	major histocompatibility complex, class II, DO alpha	ENSG00000204252	2.737	3.30E-02
HLA-DPA1	major histocompatibility complex, class II, DP alpha 1	ENSG00000231389	2.612	2.70E-03
HLA-DRA	major histocompatibility complex, class II, DR alpha	ENSG00000204287	1.890	4.20E-05
HLA-DRB1	major histocompatibility complex, class II, DR beta 1	ENSG00000196126	1.294	2.40E-02
HMGA1	high mobility group AT-hook 1	ENSG00000137309	-1.301	1.00E-03
HMGA2	high mobility group AT-hook 2	ENSG00000149948	-0.870	3.40E-02
HMGB1	high mobility group box 1	ENSG00000189403	-0.779	4.80E-02
HMGB2	high mobility group box 2	ENSG00000164104	-1.582	1.00E-04
HMGN2P3	high mobility group nucleosomal binding	ENSG00000230330	-1.759	4.20E-02

	domain 2 pseudogene 3			
HMGN2P5	high mobility group nucleosomal binding domain 2 pseudogene 5	ENSG00000234664	-1.633	1.40E-02
HMMR	hyaluronan mediated motility receptor	ENSG00000072571	-1.621	1.90E-04
HMMR-AS1	HMMR antisense RNA 1	ENSG00000251018	-1.641	5.00E-03
HSD17B6	hydroxysteroid 17-beta dehydrogenase 6	ENSG00000025423	-1.383	4.90E-02
HTRA3	HtrA serine peptidase 3	ENSG00000170801	2.910	5.00E-04
ID2	inhibitor of DNA binding 2	ENSG00000115738	1.227	3.30E-03
IGF1R	insulin like growth factor 1 receptor	ENSG00000140443	0.860	3.00E-02
IGF2BP3	insulin like growth factor 2 mRNA binding protein 3	ENSG00000136231	-0.886	3.20E-02
IGFBP3	insulin like growth factor binding protein 3	ENSG00000146674	0.955	1.80E-02
IGFBP4	insulin like growth factor binding protein 4	ENSG00000141753	1.565	1.70E-04
IGFBP5	insulin like growth factor binding protein 5	ENSG00000115461	1.103	5.10E-03
IGSF10	immunoglobulin superfamily member 10	ENSG00000152580	1.425	1.60E-02
IL11	interleukin 11	ENSG00000095752	2.098	4.50E-07
IL16	interleukin 16	ENSG00000172349	1.569	2.90E-03
IL34	interleukin 34	ENSG00000157368	-1.159	1.50E-02
IL4R	interleukin 4 receptor	ENSG00000077238	1.279	1.70E-03
INCENP	inner centromere protein	ENSG00000149503	-1.133	5.60E-03
INHA	inhibin subunit alpha	ENSG00000123999	2.440	6.80E-04
INHBA	inhibin subunit beta A	ENSG00000122641	1.257	4.70E-03
IQGAP3	IQ motif containing GTPase activating protein 3	ENSG00000183856	-1.715	4.60E-05
IRF1	interferon regulatory factor 1	ENSG00000125347	-0.819	4.30E-02
IRF8	interferon regulatory factor 8	ENSG00000140968	-1.264	1.50E-02
ISLR	immunoglobulin superfamily containing leucine rich repeat	ENSG00000129009	-1.902	7.00E-03
ISLR2	immunoglobulin superfamily containing leucine rich repeat 2	ENSG00000167178	-4.084	1.20E-03
ITGA1	integrin subunit alpha 1	ENSG00000213949	0.937	1.70E-02
ITGB1-DT	ITGB1 divergent transcript	ENSG00000229656	-2.520	7.10E-03
ITPKA	inositol-trisphosphate 3-kinase A	ENSG00000137825	-1.359	1.30E-02
ITPR3	inositol 1,4,5-trisphosphate receptor type 3	ENSG00000096433	-1.361	8.10E-04
JCAD	junctional cadherin 5 associated	ENSG00000165757	0.872	2.80E-02
JMJD1C-AS1	JMJD1C antisense RNA 1	ENSG00000272767	-1.301	3.60E-02
JUN	Jun proto-oncogene, AP-1 transcription factor subunit	ENSG00000177606	-0.939	1.80E-02
KANK3	KN motif and ankyrin repeat domains 3	ENSG00000186994	-2.015	2.10E-02
KATNA1	katanin catalytic subunit A1	ENSG00000186625	-0.932	3.80E-02
KB_1471A81		ENSG00000245330	1.455	2.30E-02
KCNB1	potassium voltage-gated channel subfamily B member 1	ENSG00000158445	3.111	1.30E-02
KCND2	potassium voltage-gated channel subfamily D member 2	ENSG00000184408	1.037	3.20E-02
KCNK12	potassium two pore domain channel subfamily K member 12	ENSG00000184261	-0.862	4.30E-02
KCNK3	potassium two pore domain channel subfamily K member 3	ENSG00000171303	-1.072	1.70E-02
KCNN4	potassium calcium-activated channel sub-	ENSG00000104783	-1.276	2.00E-03

	family N member 4			
KCNT2	potassium sodium-activated channel subfamily T member 2	ENSG00000162687	1.157	2.40E-02
KCTD16	potassium channel tetramerization domain containing 16	ENSG00000183775	1.842	3.50E-04
KIAA1958	KIAA1958	ENSG00000165185	0.922	3.60E-02
KIF11	kinesin family member 11	ENSG00000138160	-1.291	1.70E-03
KIF14	kinesin family member 14	ENSG00000118193	-1.689	7.30E-05
KIF15	kinesin family member 15	ENSG00000163808	-1.709	2.50E-04
KIF18B	kinesin family member 18B	ENSG00000186185	-1.660	1.00E-04
KIF20A	kinesin family member 20A	ENSG00000112984	-1.424	5.80E-04
KIF20B	kinesin family member 20B	ENSG00000138182	-1.367	1.10E-03
KIF22	kinesin family member 22	ENSG00000079616	-1.189	3.80E-03
KIF23	kinesin family member 23	ENSG00000137807	-1.407	7.10E-04
KIF24	kinesin family member 24	ENSG00000186638	-1.254	8.20E-03
KIF26A	kinesin family member 26A	ENSG00000066735	4.297	5.00E-17
KIF26B	kinesin family member 26B	ENSG00000162849	-1.771	5.80E-03
KIF2C	kinesin family member 2C	ENSG00000142945	-1.643	1.00E-04
KIF4A	kinesin family member 4A	ENSG00000090889	-1.590	2.00E-04
KIFC1	kinesin family member C1	ENSG00000237649	-1.796	1.80E-05
KLF10	Kruppel like factor 10	ENSG00000155090	-1.297	2.20E-03
KLF9	Kruppel like factor 9	ENSG00000119138	2.213	1.40E-05
KNL1	kinetochore scaffold 1	ENSG00000137812	-1.562	2.80E-04
KNSTRN	kinetochore localized astrin (SPAG5) binding protein	ENSG00000128944	-1.317	2.80E-03
KNTC1	kinetochore associated 1	ENSG00000184445	-0.848	4.60E-02
KPNA2	karyopherin subunit alpha 2	ENSG00000182481	-1.143	4.30E-03
KRT15	keratin 15	ENSG00000171346	-1.480	4.10E-03
KSR1	kinase suppressor of ras 1	ENSG00000141068	1.760	1.60E-05
LBH	LBH regulator of WNT signaling pathway	ENSG00000213626	0.872	3.30E-02
LCP1	lymphocyte cytosolic protein 1	ENSG00000136167	-1.448	4.00E-02
LDHD	lactate dehydrogenase D	ENSG00000166816	3.111	1.30E-02
LEF1	lymphoid enhancer binding factor 1	ENSG00000138795	-1.902	7.00E-03
LFNG	LFNG O-fucosylpeptide 3-beta-N-acetylglucosaminyltransferase	ENSG00000106003	-1.126	8.90E-03
LHX1-DT	LHX1 divergent transcript	ENSG00000277268	-3.073	1.90E-03
LHX9	LIM homeobox 9	ENSG00000143355	1.287	5.50E-03
LIMCH1	LIM and calponin homology domains 1	ENSG00000064042	1.808	8.30E-06
LIME1	Lck interacting transmembrane adaptor 1	ENSG00000203896	1.416	2.20E-02
LIN9	lin-9 DREAM MuvB core complex component	ENSG00000183814	-1.300	1.30E-02
LINC-PINT	long intergenic non-protein coding RNA, p53 induced transcript	ENSG00000231721	1.432	4.10E-03
LINC00106	long intergenic non-protein coding RNA 106	ENSG00000236871	1.212	2.70E-02
LINC00472	long intergenic non-protein coding RNA 472	ENSG00000233237	1.970	2.20E-02
LINC00511	long intergenic non-protein coding RNA 511	ENSG00000227036	-1.518	3.10E-02
LINC00624	long intergenic non-protein coding RNA 624	ENSG00000278811	-1.704	7.70E-03
LINC00685	long intergenic non-protein coding RNA 685	ENSG00000226179	3.111	1.30E-02

LINC00894	long intergenic non-protein coding RNA 894	ENSG00000235703	2.755	2.80E-04
LINC01004	long intergenic non-protein coding RNA 1004	ENSG00000228393	2.737	3.30E-02
LINC01117	long intergenic non-protein coding RNA 1117	ENSG00000224577	-1.765	1.30E-02
LINC01358	long intergenic non-protein coding RNA 1358	ENSG00000237352	-2.145	3.90E-02
LINC01485	long intergenic non-protein coding RNA 1485	ENSG00000254211	1.970	2.20E-02
LINC01605	long intergenic non-protein coding RNA 1605	ENSG00000253161	-2.324	1.50E-06
LINC02615	long intergenic non-protein coding RNA 2615	ENSG00000251432	3.315	4.80E-03
LLGL2	LLGL scribble cell polarity complex component 2	ENSG00000073350	1.075	3.00E-02
LMCD1	LIM and cysteine rich domains 1	ENSG00000071282	-1.031	4.50E-02
LMNA	lamin A/C	ENSG00000160789	-0.797	4.20E-02
LMNB1	lamin B1	ENSG00000113368	-1.625	7.80E-05
LMNB2	lamin B2	ENSG00000176619	-1.477	2.30E-04
LMO2	LIM domain only 2	ENSG00000135363	-2.698	1.30E-02
LMO4	LIM domain only 4	ENSG00000143013	0.939	1.80E-02
LOC100288637	OTU deubiquitinase 7A pseudogene	ENSG00000187951	-0.948	3.80E-02
LOC101927267	uncharacterized LOC101927267	ENSG00000258334	-2.406	1.10E-02
LOC101927552	uncharacterized LOC101927552	ENSG00000226877	2.867	6.20E-03
LOC101927751	uncharacterized LOC101927751	ENSG00000259583	-0.991	2.00E-02
LOC101928728	uncharacterized LOC101928728	ENSG00000260063	-6.961	2.80E-04
LOC102724684	uncharacterized LOC102724684	ENSG00000267026	-6.586	2.30E-03
LOC105369760		ENSG00000258017	-1.238	1.80E-03
LOC105371050		ENSG00000260874	-1.402	7.40E-03
LOC105372097		ENSG00000270112	-2.547	2.00E-02
LOC105375304	uncharacterized LOC105375304	ENSG00000225792	2.163	2.10E-02
LOC339192	uncharacterized LOC339192	ENSG00000267121	2.385	4.10E-02
LOC644135	uncharacterized LOC644135	ENSG00000237596	4.021	8.20E-10
LRR1	leucine rich repeat protein 1	ENSG00000165501	-1.103	1.60E-02
LRRC8C	leucine rich repeat containing 8 VRAC subunit C	ENSG00000171488	-0.900	4.30E-02
LRRK2	leucine rich repeat kinase 2	ENSG00000188906	1.102	3.40E-02
LYAR	Ly1 antibody reactive	ENSG00000145220	-0.979	4.00E-02
MAD2L1	mitotic arrest deficient 2 like 1	ENSG00000164109	-1.153	6.70E-03
MALL	mal, T cell differentiation protein like	ENSG00000144063	-1.710	1.10E-02
MAP1B	microtubule associated protein 1B	ENSG00000131711	-0.800	4.50E-02
MAP6D1	MAP6 domain containing 1	ENSG00000180834	-1.135	4.50E-02
MARCHF4	membrane associated ring-CH-type finger 4	ENSG00000144583	-1.744	8.80E-03
MASP1	mannan binding lectin serine peptidase 1	ENSG00000127241	1.839	1.70E-02
MB	myoglobin	ENSG00000198125	-1.134	1.70E-02
MCM10	minichromosome maintenance 10 replication initiation factor	ENSG00000065328	-1.469	9.90E-04
MCM2	minichromosome maintenance complex component 2	ENSG00000073111	-1.126	5.40E-03
MCM3	minichromosome maintenance complex component 3	ENSG00000112118	-0.833	3.70E-02

MCM4	minichromosome maintenance complex component 4	ENSG00000104738	-1.261	1.70E-03
MCM5	minichromosome maintenance complex component 5	ENSG00000100297	-1.307	1.40E-03
MCM6	minichromosome maintenance complex component 6	ENSG00000076003	-0.894	2.90E-02
MCM7	minichromosome maintenance complex component 7	ENSG00000166508	-0.821	3.90E-02
MCM8	minichromosome maintenance 8 homologous recombination repair factor	ENSG00000125885	-0.855	5.00E-02
MCOLN1	mucolipin 1	ENSG00000090674	0.841	3.90E-02
MELK	maternal embryonic leucine zipper kinase	ENSG00000165304	-1.543	2.30E-04
MET	MET proto-oncogene, receptor tyrosine kinase	ENSG00000105976	-0.914	2.80E-02
METTL7B	methyltransferase like 7B	ENSG00000170439	-1.590	8.00E-03
MGST1	microsomal glutathione S-transferase 1	ENSG00000008394	0.793	4.80E-02
MIR181A1HG	MIR181A1 host gene	ENSG00000229989	-2.008	2.80E-02
MIS18A	MIS18 kinetochore protein A	ENSG00000159055	-1.401	2.20E-03
MKI67	marker of proliferation Ki-67	ENSG00000148773	-1.705	2.50E-05
MLLT1	MLLT1 super elongation complex subunit	ENSG00000130382	0.833	3.50E-02
MLPH	melanophilin	ENSG00000115648	-3.937	7.80E-08
MMP15	matrix metallopeptidase 15	ENSG00000102996	0.964	2.60E-02
MORF4L2-AS1	MORF4L2 antisense RNA 1	ENSG00000231154	1.979	8.30E-03
MPP3	membrane palmitoylated protein 3	ENSG00000161647	-1.176	2.60E-02
MTCO2P12		ENSG00000229344	2.997	2.00E-02
MTFP1	mitochondrial fission process 1	ENSG00000242114	-1.565	1.90E-02
MTHFD1L	methylenetetrahydrofolate dehydrogenase (NADP+ dependent) 1 like	ENSG00000120254	0.905	2.40E-02
MXRA5	matrix remodeling associated 5	ENSG00000101825	2.180	1.50E-07
MXRA5Y	matrix remodeling associated 5 Y-linked (pseudogene)	ENSG00000235649	2.004	4.20E-02
MYBL1	MYB proto-oncogene like 1	ENSG00000185697	-1.287	5.10E-03
MYBL2	MYB proto-oncogene like 2	ENSG00000101057	-1.186	3.90E-03
MYEOV	myeloma overexpressed	ENSG00000172927	-1.810	4.20E-02
MYLK2	myosin light chain kinase 2	ENSG00000101306	-2.015	2.10E-02
MYRIP	myosin VIIA and Rab interacting protein	ENSG00000170011	1.323	1.20E-02
N4BP3	NEDD4 binding protein 3	ENSG00000145911	2.478	2.80E-02
NAALADL2	N-acetylated alpha-linked acidic dipeptidase like 2	ENSG00000177694	1.314	2.20E-02
NAV2	neuron navigator 2	ENSG00000166833	-1.277	5.50E-03
NCAPD2	non-SMC condensin I complex subunit D2	ENSG00000010292	-0.998	1.20E-02
NCAPG	non-SMC condensin I complex subunit G	ENSG00000109805	-1.328	1.30E-03
NCAPG2	non-SMC condensin II complex subunit G2	ENSG00000146918	-1.272	2.10E-03
NCAPH	non-SMC condensin I complex subunit H	ENSG00000121152	-1.163	9.80E-03
NDC80	NDC80 kinetochore complex component	ENSG00000080986	-1.647	1.50E-04
NDE1	nudE neurodevelopment protein 1	ENSG00000072864	-0.885	2.80E-02
NDST3	N-deacetylase and N-sulfotransferase 3	ENSG00000164100	1.074	3.30E-02
NEAT1	nuclear paraspeckle assembly transcript 1	ENSG00000245532	1.108	5.30E-03
NEBL	nebulette	ENSG00000078114	2.394	4.60E-05
NECTIN1	nectin cell adhesion molecule 1	ENSG00000110400	-0.852	3.60E-02
NEFL	neurofilament light	ENSG00000277586	-1.175	1.10E-02
NEGR1	neuronal growth regulator 1	ENSG00000172260	1.099	7.70E-03

NEK2	NIMA related kinase 2	ENSG00000117650	-1.525	9.80E-04
NEURL1B	neuralized E3 ubiquitin protein ligase 1B	ENSG00000214357	-1.421	1.50E-03
NIPAL1	NIPA like domain containing 1	ENSG00000163293	-1.321	2.80E-02
NKD2	NKD inhibitor of WNT signaling pathway 2	ENSG00000145506	1.366	1.60E-02
NPY	neuropeptide Y	ENSG00000122585	1.834	9.00E-04
NPY1R	neuropeptide Y receptor Y1	ENSG00000164128	2.470	5.80E-09
NPY5R	neuropeptide Y receptor Y5	ENSG00000164129	1.587	2.90E-03
NR4A2	nuclear receptor subfamily 4 group A member 2	ENSG00000153234	2.796	3.40E-09
NR4A3	nuclear receptor subfamily 4 group A member 3	ENSG00000119508	2.022	8.30E-03
NRGN	neurogranin	ENSG00000154146	-1.235	8.00E-03
NSD2	nuclear receptor binding SET domain protein 2	ENSG00000109685	-0.818	3.90E-02
NT5E	5'-nucleotidase ecto	ENSG00000135318	-0.985	1.40E-02
NTAN1	N-terminal asparagine amidase	ENSG00000157045	-0.903	2.90E-02
NTNG2	netrin G2	ENSG00000196358	0.928	4.50E-02
NUF2	NUF2 component of NDC80 kinetochore complex	ENSG00000143228	-1.936	1.60E-05
NUSAP1	nucleolar and spindle associated protein 1	ENSG00000137804	-1.486	3.20E-04
OCIAD1-AS1	OCIAD1 antisense RNA 1	ENSG00000248256	2.737	3.30E-02
OIP5	Opa interacting protein 5	ENSG00000104147	-1.388	1.10E-02
OLFML2A	olfactomedin like 2A	ENSG00000185585	-1.517	4.50E-04
ORC6	origin recognition complex subunit 6	ENSG00000091651	-1.478	8.20E-04
OSGIN2	oxidative stress induced growth inhibitor family member 2	ENSG00000164823	0.884	2.80E-02
PAM	peptidylglycine alpha-amidating monooxygenase	ENSG00000145730	0.877	2.70E-02
PAMR1	peptidase domain containing associated with muscle regeneration 1	ENSG00000149090	2.797	9.00E-03
PAQR4	progestin and adipoQ receptor family member 4	ENSG00000162073	-1.177	6.20E-03
PARP2	poly(ADP-ribose) polymerase 2	ENSG00000129484	-0.885	3.70E-02
PASK	PAS domain containing serine/threonine kinase	ENSG00000115687	-1.120	1.10E-02
PBK	PDZ binding kinase	ENSG00000168078	-1.467	7.10E-04
PBX1	PBX homeobox 1	ENSG00000185630	1.233	2.20E-03
PCAT6	prostate cancer associated transcript 6	ENSG00000228288	1.355	2.70E-02
PCAT7	prostate cancer associated transcript 7	ENSG00000231806	2.997	2.00E-02
PCDH1	protocadherin 1	ENSG00000156453	-1.195	1.70E-02
PCK2	phosphoenolpyruvate carboxykinase 2, mitochondrial	ENSG00000100889	-1.142	8.60E-03
PCNA	proliferating cell nuclear antigen	ENSG00000132646	-0.821	4.00E-02
PCSK4	proprotein convertase subtilisin/kexin type 4	ENSG00000115257	1.452	1.30E-02
PDE1A	phosphodiesterase 1A	ENSG00000115252	3.117	1.40E-03
PDE3A	phosphodiesterase 3A	ENSG00000172572	1.895	1.30E-02
PDE4B	phosphodiesterase 4B	ENSG00000184588	2.071	1.70E-04
PDE4D	phosphodiesterase 4D	ENSG00000113448	2.135	8.30E-06
PDE7B	phosphodiesterase 7B	ENSG00000171408	3.286	4.30E-12
PDGFD	platelet derived growth factor D	ENSG00000170962	1.704	4.90E-05
PDLIM4	PDZ and LIM domain 4	ENSG00000131435	-1.460	1.10E-03

PDZRN3	PDZ domain containing ring finger 3	ENSG00000121440	1.083	3.80E-02
PGRMC2	progesterone receptor membrane component 2	ENSG00000164040	1.024	1.10E-02
PHF19	PHD finger protein 19	ENSG00000119403	-0.933	2.20E-02
PHGDH	phosphoglycerate dehydrogenase	ENSG00000092621	-1.213	2.60E-03
PI16	peptidase inhibitor 16	ENSG00000164530	2.143	3.90E-06
PIF1	PIF1 5'-to-3' DNA helicase	ENSG00000140451	-1.155	1.20E-02
PIK3IP1	phosphoinositide-3-kinase interacting protein 1	ENSG00000100100	0.948	4.50E-02
PIMREG	PICALM interacting mitotic regulator	ENSG00000129195	-1.595	1.80E-04
PITPNM3	PITPNM family member 3	ENSG00000091622	-0.867	3.10E-02
PITX2	paired like homeodomain 2	ENSG00000164093	1.646	7.30E-03
PKMYT1	protein kinase, membrane associated tyrosine/threonine 1	ENSG00000127564	-1.296	2.00E-03
PKN3	protein kinase N3	ENSG00000160447	-0.961	3.20E-02
PKP2	plakophilin 2	ENSG00000057294	0.851	3.60E-02
PLA2G4C	phospholipase A2 group IVC	ENSG00000105499	1.481	3.80E-02
PLAT	plasminogen activator, tissue type	ENSG00000104368	-1.791	1.00E-04
PLAU	plasminogen activator, urokinase	ENSG00000122861	-1.045	8.30E-03
PLAUR	plasminogen activator, urokinase receptor	ENSG00000011422	-0.952	2.10E-02
PLCL1	phospholipase C like 1 (inactive)	ENSG00000115896	2.028	1.30E-03
PLCXD3	phosphatidylinositol specific phospholipase C X domain containing 3	ENSG00000182836	-0.945	4.80E-02
PLEK2	pleckstrin 2	ENSG00000100558	-1.366	3.30E-03
PLEKHA2	pleckstrin homology domain containing A2	ENSG00000169499	1.445	4.00E-04
PLK1	polo like kinase 1	ENSG00000166851	-1.614	9.20E-05
PLK4	polo like kinase 4	ENSG00000142731	-1.051	2.20E-02
PLOD2	procollagen-lysine,2-oxoglutarate 5-dioxygenase 2	ENSG00000152952	1.666	3.40E-05
PLSCR4	phospholipid scramblase 4	ENSG00000114698	0.821	4.30E-02
PLXNA4	plexin A4	ENSG00000221866	-1.122	1.50E-02
PLXNB3	plexin B3	ENSG00000198753	0.974	2.10E-02
PMAIP1	phorbol-12-myristate-13-acetate-induced protein 1	ENSG00000141682	-0.938	2.70E-02
PMEL	premelanosome protein	ENSG00000185664	-1.071	2.70E-02
PNP	purine nucleoside phosphorylase	ENSG00000198805	-0.879	4.30E-02
POC1A	POC1 centriolar protein A	ENSG00000164087	-1.095	1.30E-02
PODXL	podocalyxin like	ENSG00000128567	-1.731	3.00E-05
POLA1	DNA polymerase alpha 1, catalytic subunit	ENSG00000101868	-0.968	2.70E-02
POLD1	DNA polymerase delta 1, catalytic subunit	ENSG00000062822	-0.922	2.50E-02
POLE	DNA polymerase epsilon, catalytic subunit	ENSG00000177084	-0.824	4.50E-02
POLQ	DNA polymerase theta	ENSG00000051341	-1.428	2.60E-03
POLRMTP1	RNA polymerase mitochondrial pseudogene 1	ENSG00000266066	2.552	9.50E-04
PPIAP46	peptidylprolyl isomerase A pseudogene 46	ENSG00000260266	3.173	9.80E-04
PPIAP53	peptidylprolyl isomerase A pseudogene 53	ENSG00000223878	1.834	4.20E-02
PPP1R15A	protein phosphatase 1 regulatory subunit 15A	ENSG00000087074	-0.789	5.00E-02

PPP1R32	protein phosphatase 1 regulatory subunit 32	ENSG00000162148	3.217	7.80E-03
PRC1	protein regulator of cytokinesis 1	ENSG00000198901	-1.491	2.40E-04
PRC1-AS1	PRC1 antisense RNA 1	ENSG00000258725	-1.664	1.50E-04
PRDM8	PR/SET domain 8	ENSG00000152784	-1.276	1.10E-02
PREX1	phosphatidylinositol-3,4,5-trisphosphate dependent Rac exchange factor 1	ENSG00000124126	-1.079	2.40E-02
PRKN	parkin RBR E3 ubiquitin protein ligase	ENSG00000185345	1.481	3.80E-02
PRR11	proline rich 11	ENSG00000068489	-1.426	6.10E-04
PRRT3	proline rich transmembrane protein 3	ENSG00000163704	0.997	2.80E-02
PRSS2	serine protease 2	ENSG00000275896	-0.980	4.70E-02
PRUNE2	prune homolog 2 with BCH domain	ENSG00000106772	0.917	2.10E-02
PSAT1	phosphoserine aminotransferase 1	ENSG00000135069	-1.287	1.60E-03
PSD3	pleckstrin and Sec7 domain containing 3	ENSG00000156011	0.899	2.20E-02
PSRC1	proline and serine rich coiled-coil 1	ENSG00000134222	-1.591	3.90E-04
PTGS2	prostaglandin-endoperoxide synthase 2	ENSG00000073756	1.253	8.00E-03
PTP4A1	protein tyrosine phosphatase 4A1	ENSG00000112245	0.893	2.50E-02
PTPDC1	protein tyrosine phosphatase domain containing 1	ENSG00000158079	-1.233	1.30E-02
PTPRN	protein tyrosine phosphatase receptor type N	ENSG00000054356	3.267	4.00E-05
PTPRR	protein tyrosine phosphatase receptor type R	ENSG00000153233	2.153	8.50E-03
PTTG1	PTTG1 regulator of sister chromatid separation, securin	ENSG00000164611	-1.387	1.30E-03
QPRT	quinolinate phosphoribosyltransferase	ENSG00000103485	2.737	3.30E-02
RAB3A	RAB3A, member RAS oncogene family	ENSG00000105649	2.442	2.30E-04
RACGAP1	Rac GTPase activating protein 1	ENSG00000161800	-1.426	5.90E-04
RAD51	RAD51 recombinase	ENSG00000051180	-1.527	1.50E-03
RAD51AP1	RAD51 associated protein 1	ENSG00000111247	-1.689	3.10E-04
RALGAPA2	Ral GTPase activating protein catalytic alpha subunit 2	ENSG00000188559	0.892	4.10E-02
RASD1	ras related dexamethasone induced 1	ENSG00000108551	2.921	3.40E-10
RASD2	RASD family member 2	ENSG00000100302	-1.353	2.90E-02
RASL11B	RAS like family 11 member B	ENSG00000128045	2.027	3.00E-04
RASSF10	Ras association domain family member 10	ENSG00000189431	-2.834	7.80E-03
RASSF8	Ras association domain family member 8	ENSG00000123094	0.900	2.80E-02
RBL1	RB transcriptional corepressor like 1	ENSG00000080839	-1.350	2.50E-03
RBM7	RNA binding motif protein 7	ENSG00000076053	0.827	4.80E-02
RECQL4	RecQ like helicase 4	ENSG00000160957	-1.041	1.10E-02
REPS2	RALBP1 associated Eps domain containing 2	ENSG00000169891	1.121	2.20E-02
RFC2	replication factor C subunit 2	ENSG00000049541	-1.223	1.00E-02
RFC3	replication factor C subunit 3	ENSG00000133119	-1.150	6.90E-03
RGMB	repulsive guidance molecule BMP co-receptor b	ENSG00000174136	-0.880	3.00E-02
RGMB-AS1	RGMB antisense RNA 1	ENSG00000246763	-1.545	9.90E-03
RGS2	regulator of G protein signaling 2	ENSG00000116741	1.819	1.80E-05
RHBDL3	rhomboid like 3	ENSG00000141314	-1.325	1.60E-02
RHOU	ras homolog family member U	ENSG00000116574	1.984	4.20E-06
RIN1	Ras and Rab interactor 1	ENSG00000174791	-0.845	4.70E-02
RNASEH2A	ribonuclease H2 subunit A	ENSG00000104889	-1.146	5.80E-03
RND2	Rho family GTPase 2	ENSG00000108830	1.455	2.30E-02

RNF150	ring finger protein 150	ENSG00000170153	2.004	4.20E-02
RNF152	ring finger protein 152	ENSG00000176641	-2.145	3.90E-02
ROBO1	roundabout guidance receptor 1	ENSG00000169855	1.065	7.50E-03
ROBO4	roundabout guidance receptor 4	ENSG00000154133	-6.586	2.30E-03
RP11-753C18.12		ENSG00000282772	1.279	2.10E-02
RP11_1167A192		ENSG00000255038	2.306	1.50E-02
RP11_120D51		ENSG00000234129	-2.282	1.70E-02
RP11_120K92		ENSG00000259684	3.111	1.30E-02
RP11_142E91		ENSG00000276672	-2.145	3.90E-02
RP11_158H57		ENSG00000268573	3.111	1.30E-02
RP11_161H235		ENSG00000258232	-1.166	3.70E-03
RP11_19P228		ENSG00000266872	2.341	2.40E-06
RP11_229P1322		ENSG00000229257	-2.008	2.80E-02
RP11_229P1323		ENSG00000231864	-1.044	2.70E-02
RP11_22B231		ENSG00000111788	-1.263	3.10E-02
RP11_234A11		ENSG00000242299	-1.225	4.10E-02
RP11_240G225		ENSG00000274554	2.052	7.00E-03
RP11_301L82		ENSG00000233110	3.091	2.10E-05
RP11_304L198		ENSG00000261532	-1.164	1.80E-02
RP11_334A148		ENSG00000235563	2.737	3.30E-02
RP11_334C175		ENSG00000262580	1.328	1.60E-02
RP11_33N143		ENSG00000277873	-1.225	4.10E-02
RP11_344B22		ENSG00000264982	1.964	3.40E-04
RP11_344N102		ENSG00000237768	1.121	2.40E-02
RP11_399B171		ENSG00000278962	-1.916	3.00E-02
RP11_424C202		ENSG00000256663	-1.918	4.40E-03
RP11_435D73		ENSG00000232335	-1.221	4.40E-02
RP11_443B201		ENSG00000271936	-1.336	2.20E-02
RP11_454L92		ENSG00000259318	2.737	3.30E-02
RP11_459E51		ENSG00000253125	-1.218	3.00E-02
RP11_489E74		ENSG00000272256	0.793	5.00E-02
RP11_495O111		ENSG00000255091	-1.916	3.00E-02
RP11_517C164		ENSG00000261243	-1.895	3.30E-03
RP11_527N222		ENSG00000253746	-2.024	9.40E-03
RP11_544A128		ENSG00000246851	1.834	4.20E-02
RP11_54O716		ENSG00000272438	2.004	4.20E-02
RP11_613D138		ENSG00000244953	2.193	2.90E-03
RP11_626E131		ENSG00000235902	2.737	3.30E-02
RP11_65L32		ENSG00000270277	-2.282	1.70E-02
RP11_67L35		ENSG00000242396	0.986	1.50E-02
RP11_76K133		ENSG00000264475	1.903	3.10E-02
RP11_843B152		ENSG00000257530	1.562	1.00E-02
RP11_847H182		ENSG00000245482	-3.739	7.80E-03
RP11_84A193		ENSG00000254545	-1.235	1.80E-02
RP11_873E201		ENSG00000267397	1.397	6.90E-03
RP11_876N243		ENSG00000280153	3.217	7.80E-03
RP11_927P214		ENSG00000265982	6.130	6.90E-03
RP13_977J115		ENSG00000256804	-1.883	4.10E-02
RP1_228H135		ENSG00000260920	-1.773	3.10E-03
RP1_266L202		ENSG00000232640	3.515	1.20E-04
RP1_292L203		ENSG00000274818	2.163	2.10E-02
RP3_329A58		ENSG00000272374	2.873	2.00E-02
RP3_368A46		ENSG00000271533	1.834	4.20E-02

RP4_536B242		ENSG00000260466	-1.156	1.30E-02
RP4_694A72		ENSG00000233589	-1.263	3.10E-02
RP5_832C25		ENSG00000215014	2.873	2.00E-02
RP5_875H189		ENSG00000276851	-1.938	2.20E-02
RPL15P3	ribosomal protein L15 pseudogene 3	ENSG00000212802	-1.389	3.80E-02
RPS6KA2	ribosomal protein S6 kinase A2	ENSG00000071242	1.058	1.20E-02
RRAD	RRAD, Ras related glycolysis inhibitor and calcium channel regulator	ENSG00000166592	-2.285	1.10E-07
RRM1	ribonucleotide reductase catalytic subunit M1	ENSG00000167325	-0.810	4.40E-02
RRM2	ribonucleotide reductase regulatory subunit M2	ENSG00000171848	-1.171	3.80E-03
RTN4RL2	reticulon 4 receptor like 2	ENSG00000186907	6.708	7.70E-04
S100A2	S100 calcium binding protein A2	ENSG00000196754	-0.980	2.50E-02
S1PR3	sphingosine-1-phosphate receptor 3	ENSG00000213694	1.146	4.50E-03
SAPCD2	suppressor APC domain containing 2	ENSG00000186193	-1.274	2.60E-03
SAT1	spermidine/spermine N1-acetyltransferase 1	ENSG00000130066	1.142	4.90E-03
SBSPON	somatomedin B and thrombospondin type 1 domain containing	ENSG00000164764	1.822	1.60E-04
SCARA3	scavenger receptor class A member 3	ENSG00000168077	-1.628	1.20E-04
SCG2	secretogranin II	ENSG00000171951	1.944	3.30E-06
SCLY	selenocysteine lyase	ENSG00000132330	-1.524	3.10E-02
SCN2A	sodium voltage-gated channel alpha subunit 2	ENSG00000136531	-1.916	3.00E-02
SEMA3C	semaphorin 3C	ENSG00000075223	1.046	9.20E-03
SEMA3D	semaphorin 3D	ENSG00000153993	1.979	8.30E-03
SEMA3F	semaphorin 3F	ENSG00000001617	1.050	2.50E-02
SEMA6D	semaphorin 6D	ENSG00000137872	1.122	1.20E-02
SERPINE1	serpin family E member 1	ENSG00000106366	-1.431	3.20E-04
SFMBT1	Scm like with four mbt domains 1	ENSG00000163935	-1.325	1.60E-02
SFMBT2	Scm like with four mbt domains 2	ENSG00000198879	1.839	1.70E-02
SFRP1	secreted frizzled related protein 1	ENSG00000104332	1.806	2.20E-05
SGK1	serum/glucocorticoid regulated kinase 1	ENSG00000118515	0.799	4.50E-02
SGO1	shugoshin 1	ENSG00000129810	-1.375	5.80E-03
SGO2	shugoshin 2	ENSG00000163535	-0.955	3.20E-02
SHC4	SHC adaptor protein 4	ENSG00000185634	1.286	2.10E-02
SHCBP1	SHC binding and spindle associated 1	ENSG00000171241	-1.605	1.50E-04
SIDT2	SID1 transmembrane family member 2	ENSG00000149577	0.922	2.40E-02
SIGLEC15	sialic acid binding Ig like lectin 15	ENSG00000197046	-1.651	4.80E-03
SIPA1L1	signal induced proliferation associated 1 like 1	ENSG00000197555	0.898	2.60E-02
SKA1	spindle and kinetochore associated complex subunit 1	ENSG00000154839	-2.344	3.00E-06
SKA3	spindle and kinetochore associated complex subunit 3	ENSG00000165480	-1.492	1.10E-03
SKIDA1	SKI/DACH domain containing 1	ENSG00000180592	1.839	1.70E-02
SKP2	S-phase kinase associated protein 2	ENSG00000145604	-0.985	2.40E-02
SLC14A1	solute carrier family 14 member 1 (Kidd blood group)	ENSG00000141469	-1.422	6.40E-04
SLC1A3	solute carrier family 1 member 3	ENSG00000079215	3.255	3.10E-09
SLC1A7	solute carrier family 1 member 7	ENSG00000162383	1.698	6.50E-03
SLC25A30-AS1	SLC25A30 antisense RNA 1	ENSG00000251015	1.719	3.10E-02

SLC25A40	solute carrier family 25 member 40	ENSG00000075303	-1.016	4.40E-02
SLC26A2	solute carrier family 26 member 2	ENSG00000155850	0.863	3.00E-02
SLC27A3	solute carrier family 27 member 3	ENSG00000143554	-1.400	3.60E-02
SLC29A1	solute carrier family 29 member 1 (Augustine blood group)	ENSG00000112759	-0.951	2.00E-02
SLC2A12	solute carrier family 2 member 12	ENSG00000146411	2.210	6.20E-03
SLC35G1	solute carrier family 35 member G1	ENSG00000176273	-6.722	1.30E-03
SLC40A1	solute carrier family 40 member 1	ENSG00000138449	2.063	6.00E-05
SLC43A3	solute carrier family 43 member 3	ENSG00000134802	-1.242	3.80E-03
SLC44A3	solute carrier family 44 member 3	ENSG00000143036	1.135	2.00E-02
SLC44A3-AS1	SLC44A3 antisense RNA 1	ENSG00000224081	1.376	1.80E-02
SLC44A5	solute carrier family 44 member 5	ENSG00000137968	-1.447	4.10E-02
SLC46A1	solute carrier family 46 member 1	ENSG00000076351	0.953	2.30E-02
SLC4A4	solute carrier family 4 member 4	ENSG00000080493	0.892	4.70E-02
SLC4A5	solute carrier family 4 member 5	ENSG00000188687	-1.688	4.90E-03
SLC6A17	solute carrier family 6 member 17	ENSG00000197106	-2.034	1.00E-03
SLC7A5	solute carrier family 7 member 5	ENSG00000103257	-0.877	2.80E-02
SLC8A1	solute carrier family 8 member A1	ENSG00000183023	-1.559	2.60E-03
SLC8A1-AS1	SLC8A1 antisense RNA 1	ENSG00000227028	-2.698	1.30E-02
SLF1	SMC5-SMC6 complex localization factor 1	ENSG00000133302	-0.997	4.30E-02
SLFN12L	schlafen family member 12 like	ENSG00000205045	1.903	3.10E-02
SLIT2	slit guidance ligand 2	ENSG00000145147	1.504	2.00E-04
SLITRK4	SLIT and NTRK like family member 4	ENSG00000179542	1.016	1.40E-02
SMAGP	small cell adhesion glycoprotein	ENSG00000170545	-0.897	3.50E-02
SMC2	structural maintenance of chromosomes 2	ENSG00000136824	-0.989	1.70E-02
SMC4	structural maintenance of chromosomes 4	ENSG00000113810	-1.167	3.60E-03
SMC6	structural maintenance of chromosomes 6	ENSG00000163029	-1.095	9.20E-03
SMPD3	sphingomyelin phosphodiesterase 3	ENSG00000103056	1.244	4.00E-03
SMTN	smoothelin	ENSG00000183963	-0.894	5.00E-02
SNAI3	snail family transcriptional repressor 3	ENSG00000185669	2.095	1.20E-02
SNRPGP10	small nuclear ribonucleoprotein polypeptide G pseudogene 10	ENSG00000235363	-1.916	3.00E-02
SORBS2	sorbin and SH3 domain containing 2	ENSG00000154556	2.165	4.90E-07
SOX6	SRY-box transcription factor 6	ENSG00000110693	-1.447	4.10E-02
SOX9	SRY-box transcription factor 9	ENSG00000125398	-1.107	7.80E-03
SPAG5	sperm associated antigen 5	ENSG00000076382	-1.326	1.80E-03
SPDL1	spindle apparatus coiled-coil protein 1	ENSG00000040275	-1.390	7.40E-04
SPHK1	sphingosine kinase 1	ENSG00000176170	-2.609	3.30E-06
SPINK4	serine peptidase inhibitor, Kazal type 4	ENSG00000122711	6.266	3.90E-03
SPOCD1	SPOC domain containing 1	ENSG00000134668	-1.358	1.00E-03
SPOCK3	SPARC (osteonectin), cwcv and kazal like domains proteoglycan 3	ENSG00000196104	1.049	1.70E-02
SRGN	serglycin	ENSG00000122862	1.169	1.00E-02
SSPO	SCO-spondin	ENSG00000197558	1.281	4.20E-02
ST20-MTHFS	ST20-MTHFS readthrough	ENSG00000259332	1.970	2.20E-02
ST3GAL6-AS1	ST3GAL6 antisense RNA 1	ENSG00000239445	2.997	2.00E-02
STAR	steroidogenic acute regulatory protein	ENSG00000147465	1.736	3.20E-05
STARD5	StAR related lipid transfer domain containing 5	ENSG00000172345	1.060	2.80E-02
STBD1	starch binding domain 1	ENSG00000118804	-1.400	3.60E-02
STC1	stanniocalcin 1	ENSG00000159167	1.226	4.30E-03
STIL	STIL centriolar assembly protein	ENSG00000123473	-1.140	9.60E-03
STMN1	stathmin 1	ENSG00000117632	-0.915	2.10E-02

STRA6	stimulated by retinoic acid 6	ENSG00000137868	-1.507	1.70E-03
STX1A	syntaxin 1A	ENSG00000106089	-1.291	3.60E-03
SULT1B1	sulfotransferase family 1B member 1	ENSG00000173597	3.217	7.80E-03
SUV39H1	suppressor of variegation 3-9 homolog 1	ENSG00000101945	-0.893	5.00E-02
SYBU	syntabulin	ENSG00000147642	1.270	3.10E-02
SYNE3	spectrin repeat containing nuclear envelope family member 3	ENSG00000176438	-2.569	9.60E-05
TACC3	transforming acidic coiled-coil containing protein 3	ENSG00000013810	-1.389	6.40E-04
TANC2	tetratricopeptide repeat, ankyrin repeat and coiled-coil containing 2	ENSG00000170921	0.809	4.30E-02
TBC1D2B	TBC1 domain family member 2B	ENSG00000167202	-0.822	4.40E-02
TBL1X	transducin beta like 1 X-linked	ENSG00000101849	1.119	4.90E-03
TBX2	T-box transcription factor 2	ENSG00000121068	1.543	2.20E-04
TCF19	transcription factor 19	ENSG00000137310	-1.186	4.10E-03
TCOF1	treacle ribosome biogenesis factor 1	ENSG00000070814	-0.895	2.70E-02
TEDC1	tubulin epsilon and delta complex 1	ENSG00000185347	-0.890	4.50E-02
TENM4	teneurin transmembrane protein 4	ENSG00000149256	-0.936	4.40E-02
TEX52	testis expressed 52	ENSG00000283297	2.997	2.00E-02
TFPI	tissue factor pathway inhibitor	ENSG00000003436	1.030	1.00E-02
TFPI2	tissue factor pathway inhibitor 2	ENSG00000105825	1.067	2.20E-02
TGFBR2	transforming growth factor beta receptor 2	ENSG00000163513	0.822	3.90E-02
TGM2	transglutaminase 2	ENSG00000198959	1.195	3.50E-03
TICRR	TOPBP1 interacting checkpoint and replication regulator	ENSG00000140534	-1.127	1.00E-02
TIGD3	tigger transposable element derived 3	ENSG00000173825	2.737	3.30E-02
TIMELESS	timeless circadian regulator	ENSG00000111602	-0.980	1.60E-02
TK1	thymidine kinase 1	ENSG00000167900	-1.468	3.40E-04
TLCD2	TLC domain containing 2	ENSG00000185561	1.119	4.70E-02
TLL1	tolloid like 1	ENSG00000038295	1.307	8.20E-03
TM4SF1	transmembrane 4 L six family member 1	ENSG00000169908	1.756	2.20E-05
TM7SF2	transmembrane 7 superfamily member 2	ENSG00000149809	1.178	6.20E-03
TMC3	transmembrane channel like 3	ENSG00000188869	3.653	3.10E-03
TMEFF2	transmembrane protein with EGF like and two follistatin like domains 2	ENSG00000144339	1.985	2.20E-05
TMEM151A	transmembrane protein 151A	ENSG00000179292	1.495	5.70E-03
TMEM171	transmembrane protein 171	ENSG00000157111	-2.406	1.10E-02
TMEM190	transmembrane protein 190	ENSG00000160472	1.780	2.30E-02
TMEM245	transmembrane protein 245	ENSG00000106771	0.817	3.90E-02
TMEM25	transmembrane protein 25	ENSG00000149582	0.867	4.10E-02
TMEM60	transmembrane protein 60	ENSG00000135211	-1.016	4.40E-02
TMPO	thymopoietin	ENSG00000120802	-0.924	2.10E-02
TMPO-AS1	TMPO antisense RNA 1	ENSG00000257167	-1.385	9.30E-04
TMTC1	transmembrane O-mannosyltransferase targeting cadherins 1	ENSG00000133687	1.088	1.00E-02
TNFAIP8L1	TNF alpha induced protein 8 like 1	ENSG00000185361	-0.913	3.00E-02
TNFRSF11A	TNF receptor superfamily member 11a	ENSG00000141655	-1.077	2.80E-02
TNFRSF11B	TNF receptor superfamily member 11b	ENSG00000164761	1.475	2.10E-03
TNFRSF21	TNF receptor superfamily member 21	ENSG00000146072	0.864	3.20E-02
TNFSF10	TNF superfamily member 10	ENSG00000121858	1.578	5.30E-04
TOB1	transducer of ERBB2, 1	ENSG00000141232	1.907	5.90E-06
TOP2A	DNA topoisomerase II alpha	ENSG00000131747	-1.695	2.80E-05

TP73	tumor protein p73	ENSG00000078900	-1.689	1.10E-02
TPTEP2	TPTE pseudogene 2	ENSG00000244627	1.637	2.30E-02
TPX2	TPX2 microtubule nucleation factor	ENSG00000088325	-1.649	5.50E-05
TRAC	T cell receptor alpha constant	ENSG00000277734	-2.834	7.80E-03
TRHDE	thyrotropin releasing hormone degrading enzyme	ENSG00000072657	-1.131	2.40E-02
TRIM59	tripartite motif containing 59	ENSG00000213186	-1.208	3.50E-03
TRIP13	thyroid hormone receptor interactor 13	ENSG00000071539	-1.264	3.30E-03
TROAP	trophinin associated protein	ENSG00000135451	-1.705	1.30E-04
TRPC6	transient receptor potential cation channel subfamily C member 6	ENSG00000137672	1.567	1.90E-02
TSPAN11	tetraspanin 11	ENSG00000110900	1.094	1.00E-02
TSPAN8	tetraspanin 8	ENSG00000127324	0.806	4.60E-02
TTC36	tetratricopeptide repeat domain 36	ENSG00000172425	2.873	2.00E-02
TTF2	transcription termination factor 2	ENSG00000116830	-1.274	3.50E-03
TTK	TTK protein kinase	ENSG00000112742	-1.754	1.10E-04
TUB	TUB bipartite transcription factor	ENSG00000166402	1.299	3.40E-03
TUBA1B	tubulin alpha 1b	ENSG00000123416	-1.233	1.80E-03
TUBA1C	tubulin alpha 1c	ENSG00000167553	-1.229	2.00E-03
TUBB	tubulin beta class I	ENSG00000196230	-0.798	4.20E-02
TUBB4B	tubulin beta 4B class IVb	ENSG00000188229	-1.195	2.60E-03
TVP23C	trans-golgi network vesicle protein 23 homolog C	ENSG00000175106	1.360	9.00E-03
TYMS	thymidylate synthetase	ENSG00000176890	-1.178	3.50E-03
TYMSOS	TYMS opposite strand	ENSG00000176912	-1.497	7.90E-04
TYRP1	tyrosinase related protein 1	ENSG00000107165	1.227	2.90E-02
UBE2C	ubiquitin conjugating enzyme E2 C	ENSG00000175063	-1.795	3.00E-05
UBE2T	ubiquitin conjugating enzyme E2 T	ENSG00000077152	-1.366	2.40E-03
UBL7-AS1	UBL7 antisense RNA 1 (head to head)	ENSG00000247240	-1.759	4.20E-02
UHRF1	ubiquitin like with PHD and ring finger domains 1	ENSG00000276043	-1.527	2.00E-04
UNC5D	unc-5 netrin receptor D	ENSG00000156687	2.001	9.40E-03
UPP1	uridine phosphorylase 1	ENSG00000183696	1.138	4.40E-03
USP1	ubiquitin specific peptidase 1	ENSG00000162607	-0.913	2.50E-02
UST-AS1	UST antisense RNA 1	ENSG00000227660	1.034	4.80E-02
VASH2	vasohibin 2	ENSG00000143494	0.832	4.50E-02
VDR	vitamin D receptor	ENSG00000111424	1.568	2.60E-03
VSTM2L	V-set and transmembrane domain containing 2 like	ENSG00000132821	-1.614	4.10E-02
VTN	vitronectin	ENSG00000109072	1.572	1.60E-03
VWA5B2	von Willebrand factor A domain containing 5B2	ENSG00000145198	4.839	1.50E-14
WDHD1	WD repeat and HMG-box DNA binding protein 1	ENSG00000198554	-1.224	5.20E-03
WDR62	WD repeat domain 62	ENSG00000075702	-1.801	1.60E-04
WIPI1	WD repeat domain, phosphoinositide interacting 1	ENSG00000070540	0.841	4.00E-02
WLS	Wnt ligand secretion mediator	ENSG00000116729	-0.930	1.90E-02
WNT2B	Wnt family member 2B	ENSG00000134245	1.292	5.00E-03
WNT5A	Wnt family member 5A	ENSG00000114251	1.069	8.80E-03
WNT5A-AS1	WNT5A antisense RNA 1	ENSG00000244586	1.761	7.00E-04
WNT7B	Wnt family member 7B	ENSG00000188064	-2.905	1.40E-03
XXBAC_BPG252P99		ENSG00000272540	-0.864	2.80E-02

YPEL4	yippee like 4	ENSG00000166793	1.227	1.70E-02
ZGRF1	zinc finger GRF-type containing 1	ENSG00000138658	-1.298	9.50E-03
ZKSCAN7-AS1	ZKSCAN7 ZNF cluster antisense RNA 1	ENSG00000236869	-2.015	2.10E-02
ZNF275	zinc finger protein 275	ENSG00000063587	0.986	1.40E-02
ZNF33B	zinc finger protein 33B	ENSG00000196693	1.107	2.50E-02
ZNF367	zinc finger protein 367	ENSG00000165244	-1.239	8.10E-03
ZNF436-AS1	ZNF436 antisense RNA 1	ENSG00000249087	1.081	2.70E-02
ZNF641	zinc finger protein 641	ENSG00000167528	0.842	4.40E-02
ZNF703	zinc finger protein 703	ENSG00000183779	-0.860	3.10E-02
ZNF714	zinc finger protein 714	ENSG00000160352	-1.537	4.10E-03
ZWILCH	zwilch kinetochore protein	ENSG00000174442	-1.024	1.60E-02
ZWINT	ZW10 interacting kinetochore protein	ENSG00000122952	-1.281	1.70E-03

Table S3. List of upstream regulators in treatments (melatonin, FSK) (*p*-value of overlap ≥ 0.05).

**Melatonin Treatment**					
**Upstream Regulator**	**Expr Log Ratio**	**Molecule Type**	**Predicted Activation State**	**Activation z-score**	**p-value of overlap**
**DAP3**	0.150	other	Inhibited	-2.449	2.64E-06
**MT-TE**	-0.418	other			4.96E-05
**LONP1**	-0.026	peptidase		-0.522	7.91E-05
**STOX1**		other			8.31E-05
**actinonin**		chemical reagent	Activated	2.236	2.35E-04
**HIF1A**	0.061	transcription regulator		0.520	6.20E-04
**MALSU1**	-0.089	other			8.09E-04
**Ap2 alpha**		group			8.09E-04
**MRPL14**	-0.200	other			1.38E-03
**trans-hydroxytamoxifen**		chemical drug		-0.775	1.97E-03
**SIRT3**	-0.080	enzyme	Activated	2.020	1.99E-03
**Retinoic acid-RAR-RXR**		complex			2.15E-03
**ALKBH1**	-0.248	enzyme			2.15E-03
**NSUN3**	0.269	enzyme			2.15E-03
**GPER1**	-1.153	G-protein coupled receptor		1.242	2.23E-03
**PHB2**	0.083	transcription regulator		1.000	2.54E-03
**HNF4A**		transcription regulator		-0.170	3.07E-03
**ARHGDIG**		other		-1.000	3.18E-03
**IFITM1**	-0.645	transmembrane receptor	Activated	2.000	3.18E-03
**miR-892b (miRNAs w/seed ACUGGCU)**		mature microRNA			3.65E-03
**taprostene**		chemical reagent			3.65E-03
**ACSL4**	0.007	enzyme		-0.391	3.71E-03
**PPARGC1B**		transcription regulator		-1.134	3.80E-03
**hydrocortisone**		chemical - endogenous mammalian		-1.086	4.32E-03
**LCOR**	-0.109	transcription regulator			4.37E-03
**EIF4E**	-0.003	translation regulator		-1.414	5.28E-03
**FLCN**	-0.361	other	Activated	2.214	6.44E-03
**miR-331-3p (miRNAs w/seed CCCCUGG)**		mature microRNA			7.12E-03
**PAPOLA**	0.048	enzyme			7.12E-03
**PAF1**	-0.087	other		0.447	7.30E-03
**torin1**		chemical reagent		1.718	7.81E-03
**MIR4269**		microRNA			1.16E-02
**Cox5b**		other			1.16E-02
**pemetrexed**		chemical drug			1.16E-02
**Betacatenin/TCF**		complex			1.19E-02
**TFAP2B**		transcription regulator			1.19E-02

**AKT3**	0.146	kinase			1.24E-02
**MYOC**		other			1.25E-02
**phenethyl isothiocyanate**		chemical drug		-1.000	1.35E-02
**MT-TM**		other			1.45E-02
**CNOT3**	0.060	other			1.45E-02
**RICTOR**	0.110	other		1.780	1.53E-02
**NR4A1**	0.077	ligand-dependent nuclear receptor		0.905	1.53E-02
**RRP1B**	0.149	transcription regulator			1.59E-02
**AP2M1**	-0.138	transporter			1.70E-02
**TAB2**	0.097	other			1.70E-02
**FGF18**		growth factor			1.70E-02
**Congo Red**		chemical toxicant			1.70E-02
**calcium chloride**		chemical drug			1.70E-02
**Rhox5**		transcription regulator			1.74E-02
**1810019D21Rik**		other		-0.816	1.75E-02
**ASXL2**	-0.159	other		-1.000	1.82E-02
**bexarotene**		chemical drug		-0.826	1.98E-02
**miR-21-5p (and other miRNAs w/seed AGCUUAU)**		mature microRNA	Inhibited	-2.410	2.04E-02
**coenzyme Q10**		chemical drug			2.06E-02
**cerulenin**		chemical drug			2.06E-02
**TFE3**	0.010	transcription regulator	Inhibited	-2.236	2.25E-02
**CNOT7**	0.018	transcription regulator			2.28E-02
**ZNF746**	0.098	transcription regulator			2.32E-02
**KMT5B**	-0.041	enzyme			2.32E-02
**LRPPRC**	0.136	other			2.32E-02
**WNT10B**	0.056	other			2.32E-02
**ASARM-PO4**		chemical reagent			2.32E-02
**MYCN**		transcription regulator	Inhibited	-3.208	2.38E-02
**MAP4K4**	-0.071	kinase		-0.426	2.40E-02
**HNF1B**		transcription regulator		0.889	2.40E-02
**NFIA**	0.819	transcription regulator			2.41E-02
**PDGF BB**		complex		0.045	2.59E-02
**mir-181**		microRNA		1.000	2.65E-02
**4-hydroxytamoxifen**		chemical drug		0.095	2.74E-02
**EPAS1**	0.186	transcription regulator		0.059	2.74E-02
**BACH1**	0.126	transcription regulator			2.79E-02
**F2R**	0.006	G-protein coupled receptor		-0.518	2.94E-02
**6-amino-4-(4-**		chemical reagent			3.03E-02

**phenoxyphenylethylamino)quinazoline**				
**L-type Calcium Channel**		complex		3.03E-02
**MRPL12**	-0.142	other		3.03E-02
**miR-217-5p (and other miRNAs w/seed ACUGCAU)**		mature microRNA		3.03E-02
**RGS16**		other		3.03E-02
**miR-16-5p (and other miRNAs w/seed AG-CAGCA)**		mature microRNA	-0.430	3.05E-02
**AHR**	0.231	ligand-dependent nuclear receptor	-0.900	3.15E-02
**LIPE**	-0.493	enzyme		3.18E-02
**ZNF202**	0.542	transcription regu-lator		3.19E-02
**ADRB1**		G-protein coupled receptor		3.19E-02
**LMNA**	-0.013	other		3.36E-02
**atractyloside**		chemical toxicant		3.53E-02
**endocannabinoid**		chemical - endog-enous mammalian		3.53E-02
**tubulin (complex)**		complex		3.53E-02
**ASB9**	-0.630	transcription regu-lator		3.53E-02
**WTIP**	0.074	transcription regu-lator		3.53E-02
**DNAJA4**	-1.703	other		3.53E-02
**TFAP2E**	0.341	transcription regu-lator		3.53E-02
**ANKRD12**	0.089	other		3.53E-02
**BRINP1**	0.743	peptidase		3.53E-02
**PPIP5K1**	0.157	phosphatase		3.53E-02
**VPS36**	0.094	other		3.53E-02
**FNDC3B**	0.100	other		3.53E-02
**TMEM184A**	-0.094	other		3.53E-02
**DMTN**	0.072	other		3.53E-02
**SGMS2**	0.172	enzyme		3.53E-02
**CDCA4**	0.106	other		3.53E-02
**RIC8A**	0.009	other		3.53E-02
**PU-H71**		chemical drug		3.53E-02
**SYTL4**	0.125	transporter		3.53E-02
**AP2S1**	0.048	transporter		3.53E-02
**MORF4L2**	0.027	other		3.53E-02
**JAKMIP1**		translation regula-tor		3.53E-02
**SNAP91**		other		3.53E-02
**CFI**	0.007	peptidase		3.53E-02
**mir-3960**		microRNA		3.53E-02
**miR-377-3p (miRNAs w/seed UCACACA)**		mature microRNA		3.53E-02
**miR-548d-3p (and other miRNAs w/seed AAAAACC)**		mature microRNA		3.53E-02
**miR-331-5p (and other miRNAs w/seed UAGGUAU)**		mature microRNA		3.53E-02

**miR-616-3p (miRNAs w/seed GUCAUUG)**		mature microRNA		3.53E-02
**AX 115**		chemical reagent		3.53E-02
**SCO2**	-0.219	enzyme		3.53E-02
**KCNA1**		ion channel		3.53E-02
**SEM1**	-0.108	peptidase		3.53E-02
**MAP7**	-0.599	other		3.53E-02
**ECSCR**		other		3.53E-02
**ATP1B3**	-0.066	transporter		3.53E-02
**CRABP1**		transporter		3.53E-02
**CFD**		peptidase		3.53E-02
**CAND1**	0.116	transcription regulator		3.53E-02
**chemotherapy/tretinoin**		chemical drug		3.53E-02
**MORF4L1**	0.025	other		3.53E-02
**ALYREF**	-0.095	transcription regulator		3.53E-02
**DECR1**	-0.037	enzyme		3.53E-02
**CHP1**	-0.141	transporter		3.53E-02
**OPRL1**	0.056	G-protein coupled receptor		3.53E-02
**IGHMBP2**	-0.003	enzyme		3.53E-02
**SR11256**		chemical reagent		3.53E-02
**RECTAS**		chemical reagent		3.53E-02
**broussoflavonol b**		chemical reagent		3.53E-02
**Annonacin**		chemical reagent		3.53E-02
**Clorgyline**		chemical drug		3.53E-02
**4-diphenylacetoxy-1,1-dimethylpiperidinium**		chemical reagent		3.53E-02
**Thioperamide**		chemical reagent		3.53E-02
**2,5-dihydroxymethylcinnamate**		chemical - kinase inhibitor		3.53E-02
**Nafoxidine**		chemical drug		3.53E-02
**Astra 1397**		chemical reagent		3.53E-02
**TAK-779**		chemical drug		3.53E-02
**PD173955**		chemical - kinase inhibitor		3.53E-02
**Fotemustine**		chemical drug		3.53E-02
**quinuclidinyl benzilate**		chemical reagent		3.53E-02
**AP-1 decoy**		chemical reagent		3.53E-02
**tartaric acid**		chemical reagent		3.53E-02
**pristanic acid**		chemical - endogenous mammalian		3.53E-02
**2-ethylhexanoic acid**		chemical toxicant		3.53E-02
**Cycloleucine**		chemical toxicant		3.53E-02
**hydrocortisone phosphate**		chemical drug		3.53E-02
**Tin**		chemical reagent		3.53E-02
**FN1**	0.037	enzyme	-0.529	3.61E-02
**MAT1A**		enzyme		3.63E-02
**(-)-epicatechin gallate**		chemical - endogenous non-mammalian		3.80E-02

**TRG**		other			3.80E-02
**PHEX**	0.544	peptidase			3.80E-02
**Chromium**		chemical drug		-0.555	3.86E-02
**NORAD**	0.111	other		-1.000	4.05E-02
**PTH**		other		0.152	4.06E-02
**Raloxifene**		chemical drug		-0.164	4.06E-02
**L-tryptophan**		chemical - endogenous mammalian			4.10E-02
**WDR5**	-0.068	transcription regulator			4.10E-02
**PBX3**	0.124	transcription regulator			4.10E-02
**Apicidin**		chemical - endogenous non-mammalian			4.10E-02
**RASSF1**	-0.234	other		-1.066	4.20E-02
**MAP2K1**	0.141	kinase		-0.181	4.30E-02
**SOX1**		transcription regulator		0.000	4.40E-02
**CAB39L**	0.536	kinase		-1.000	4.41E-02
**CIDEC**		other		0.686	4.41E-02
**Erlotinib**		chemical drug		0.956	4.41E-02
**oblimersen**		biologic drug		0.447	4.42E-02
**PLAG1**	-0.122	transcription regulator		-1.432	4.42E-02
**alvespimycin**		chemical drug			4.59E-02
**MBD1**	-0.107	transcription regulator			4.59E-02
**cycloheximide**		chemical reagent		-0.701	4.60E-02
**NKX2-3**		transcription regulator		-0.873	4.63E-02
**PHF21A**	-0.051	other			4.64E-02
**MIR320**		group			4.64E-02
**NMNAT1**	0.145	enzyme			4.64E-02
**lapatinib**		chemical drug			4.64E-02
**levamisole**		chemical drug			4.64E-02
**ibandronic acid**		chemical drug			4.64E-02
**cortistatin A**		chemical reagent			4.64E-02
**tiron**		chemical reagent			4.64E-02
**miR-125b-5p (and other miRNAs w/seed CCCUGAG)**		mature microRNA	Inhibited	-2.590	4.86E-02
**TREM1**		transmembrane receptor		-0.577	4.89E-02
**sirolimus**		chemical drug	Activated	3.313	6.92E-02
**RUNX3**		transcription regulator	Inhibited	-2.646	7.82E-02
**NEUROG1**		transcription regulator	Activated	2.236	7.93E-02
**KLF3**	-0.123	transcription regulator	Inhibited	-2.183	8.32E-02
**TOB1**	-0.204	transcription regu-	Activated	2.000	8.87E-02

		lator			
**TRAP1**	0.078	enzyme	Activated	2.449	9.48E-02
**ATF4**	-0.144	transcription regulator	Activated	2.154	1.17E-01
**PGR**	0.235	ligand-dependent nuclear receptor	Inhibited	-2.104	1.20E-01
**carboplatin**		chemical drug		-1.953	1.29E-01
**trichostatin A**		chemical drug	Inhibited	-2.044	1.31E-01
**MSC**	-0.038	transcription regulator	Activated	2.000	1.42E-01
**NRIP1**	0.185	transcription regulator		1.961	1.68E-01
**EGLN1**	0.085	enzyme		-1.982	1.75E-01
**metribolone**		chemical reagent	Inhibited	-2.706	1.90E-01
**CLPP**	-0.025	peptidase	Activated	2.000	1.97E-01
**decitabine**		chemical drug		-1.911	2.03E-01
**trovafloxacin**		chemical drug	Inhibited	-2.000	2.19E-01
**PTEN**	0.141	phosphatase	Inhibited	-2.210	2.30E-01
**heparin**		chemical - endogenous mammalian		1.969	2.57E-01
**tazemetostat**		chemical drug		-1.994	2.60E-01
**MTOR**	0.059	kinase		-1.795	2.61E-01
**SP2509**		chemical reagent		-1.994	3.06E-01
**STK11**	0.053	kinase	Inhibited	-2.331	3.24E-01
**LCN2**		transporter		1.951	3.61E-01
**PPARD**	0.039	ligand-dependent nuclear receptor	Activated	2.803	3.79E-01
**KDM1A**	0.082	enzyme		1.964	4.00E-01
**mir-21**		microRNA	Inhibited	-2.529	4.02E-01
**CXCL12**		cytokine		-1.942	4.05E-01
**okadaic acid**		chemical toxicant		-1.982	4.25E-01
**SP600125**		chemical - kinase inhibitor	Inhibited	-2.230	4.79E-01
**KLF11**	-0.231	transcription regulator		-1.913	4.80E-01
**CDKN2A**		transcription regulator		-1.929	4.83E-01
**HSF1**	-0.013	transcription regulator		1.777	5.01E-01
**carbon tetrachloride**		chemical toxicant	Inhibited	-2.234	5.01E-01
**PRKCD**	-0.377	kinase		1.890	5.08E-01
**fatty acid**		chemical - endogenous mammalian		1.960	5.33E-01
**isobutylmethylxanthine**		chemical toxicant		1.941	1.00E00
**5-fluorouracil**		chemical drug		1.912	1.00E00
**etoposide**		chemical drug		-1.982	1.00E00
**17-alpha-ethinylestradiol**		chemical drug		-1.982	1.00E00
**Pkc(s)**		group		1.987	1.00E00
**26s Proteasome**		complex		1.982	1.00E00
**CCND1**	-0.063	transcription regulator		1.969	1.00E00

**APOE**	-0.592	transporter		-1.944	1.00E00
**GATA6**	0.135	transcription regulator	Activated	2.000	1.00E00
**YAP1**	-0.014	transcription regulator		1.949	1.00E00
**NFATC2**	0.207	transcription regulator	Activated	2.000	1.00E00
**TCF7L2**	-0.111	transcription regulator		1.890	1.00E00
**EPO**		cytokine		1.982	1.00E00
**WNT3A**		cytokine		1.828	1.00E00
**pioglitazone**		chemical drug	Inhibited	-2.160	1.00E00
**dimethyl sulfoxide**		chemical drug	Activated	2.000	1.00E00
**isoproterenol**		chemical drug	Activated	2.166	1.00E00
**cyclosporin A**		biologic drug		-1.849	1.00E00
**D-glucose**		chemical - endogenous mammalian		1.847	1.00E00
**wortmannin**		chemical - kinase inhibitor		1.969	1.00E00
**testosterone**		chemical - endogenous mammalian	Inhibited	-2.270	1.00E00
**Ca2+**		chemical – endogenous mammalian	Activated	2.959	1.00E00
**FSK treatment**					
**Upstream Regulator**	Expr Log Ratio	Molecule Type	Predicted Activation State	Activation z-score	p-value of overlap
**ZBTB17**	-0.243	transcription regulator			1.55E-26
**Vegf**		group	Inhibited	-2.090	5.96E-18
**dexamethasone**		chemical drug	Activated	2.176	4.18E-16
**PTGER2**	-0.527	G-protein coupled receptor	Inhibited	-3.394	1.09E-15
**TP53**	-0.267	transcription regulator		1.729	5.11E-15
**HGF**		growth factor	Inhibited	-2.072	7.72E-15
**CDKN1A**	0.081	kinase		1.907	8.50E-15
**forskolin**		chemical toxicant	Activated	3.974	1.10E-14
**FOXM1**	-1.262	transcription regulator	Inhibited	-3.168	8.99E-14
**NR1H3**	0.652	ligand-dependent nuclear receptor			1.10E-13
**AREG**	0.650	growth factor	Inhibited	-3.357	3.02E-13
**E2F4**	-0.407	transcription regulator			1.10E-12
**RABL6**	-0.248	other	Inhibited	-3.606	1.38E-12
**CCND1**	-0.747	transcription regulator	Inhibited	-2.121	7.35E-12
**TCF3**	-0.367	transcription regulator		1.667	8.34E-12
**CREB1**	0.091	transcription regu-		0.312	9.32E-12

		lator			
**Pka**		complex	Activated	2.408	3.77E-11
**CDK4**	-0.560	kinase			4.20E-11
**dextran sulfate**		chemical drug			1.06E-10
**LDLR**	0.325	transporter			3.32E-10
**TGFB1**	-0.079	growth factor		-0.136	9.14E-10
**TCF4**	0.186	transcription regulator			1.21E-09
**FOXO3**	0.142	transcription regulator		0.730	1.29E-09
**HNF1A-AS1**		other	Inhibited	-2.646	2.06E-09
**ERBB2**	0.230	kinase	Inhibited	-2.904	2.84E-09
**FGF2**	0.116	growth factor		-1.436	3.84E-09
**calcitriol**		chemical drug	Activated	3.442	4.13E-09
**LIN9**	-1.300	other	Inhibited	-2.619	4.65E-09
**CDKN2A**		transcription regulator	Activated	3.605	8.67E-09
**EGF**	1.097	growth factor		-0.337	1.06E-08
**CSF2**		cytokine	Inhibited	-3.266	1.54E-08
**ESR1**	1.726	ligand-dependent nuclear receptor	Inhibited	-2.021	3.53E-08
**IL1B**	0.343	cytokine		0.934	3.85E-08
**actinomycin D**		biologic drug		-1.296	7.21E-08
**PHF21A**	0.234	other	Activated	2.190	7.30E-08
**YAP1**	0.072	transcription regulator		-1.937	8.11E-08
**diethylstilbestrol**		chemical drug		-1.528	8.96E-08
**YY1**	-0.116	transcription regulator			1.09E-07
**NUPR1**	-0.122	transcription regulator	Activated	2.711	1.25E-07
**AIP**	0.199	transcription regulator	Inhibited	-2.138	1.65E-07
**2-(4-amino-1-isopropyl-1H-pyrazolo[3,4-d]pyrimidin-3-yl)-1H-indol-5-ol**		chemical reagent	Activated	2.985	1.80E-07
**fluocinolone acetonide**		chemical drug		-0.707	1.94E-07
**AR**	-0.158	ligand-dependent nuclear receptor		-1.808	3.58E-07
**MITF**	0.133	transcription regulator	Inhibited	-3.189	3.63E-07
**TNF**		cytokine		0.329	5.74E-07
**CREM**	0.226	transcription regulator		-0.622	6.12E-07
**8-bromo-cAMP**		chemical reagent	Activated	2.429	6.40E-07
**beta-estradiol**		chemical - endogenous mammalian		1.142	6.65E-07
**IL6**	0.549	cytokine		0.254	7.16E-07
**tetradecanoylphorbol acetate**		chemical drug		0.649	8.36E-07
**EP400**	-0.207	other	Inhibited	-2.621	8.60E-07
**filgrastim**		biologic drug		1.023	9.40E-07
**LY294002**		chemical - kinase		-0.282	9.59E-07

		inhibitor			
**AGN194204**		chemical drug	Inhibited	-2.121	1.16E-06
**PTH**		other		0.554	1.86E-06
**tretinoin**		chemical - endogenous mammalian	Activated	2.038	2.03E-06
**prostaglandin E2**		chemical - endogenous mammalian		1.926	2.62E-06
**doxorubicin**		chemical drug	Activated	2.282	2.68E-06
**triamcinolone acetonide**		chemical drug		0.507	2.82E-06
**RBPJ**	-0.023	transcription regulator		0.632	2.87E-06
**GW3965**		chemical reagent		1.088	2.92E-06
**4-hydroxytamoxifen**		chemical drug		0.380	3.37E-06
**decitabine**		chemical drug		1.453	3.72E-06
**Nr1h**		group		0.250	3.97E-06
**Pkc(s)**		group	Activated	2.376	4.47E-06
**TP73**	-1.689	transcription regulator	Activated	2.238	4.57E-06
**PDGF BB**		complex		1.320	4.94E-06
**P38 MAPK**		group		1.232	5.89E-06
**WNT1**		cytokine		-0.742	6.43E-06
**cyclic AMP**		chemical - endogenous mammalian	Activated	2.801	7.07E-06
**LHCGR**		G-protein coupled receptor			7.42E-06
**AGT**		growth factor		0.927	7.71E-06
**deferoxamine**		chemical drug		0.389	8.46E-06
**discodermolide**		chemical drug			8.68E-06
**KDM5B**	0.143	transcription regulator	Activated	2.039	8.97E-06
**GATA4**	0.233	transcription regulator		0.862	1.09E-05
**hydroxyurea**		chemical drug		-0.922	1.13E-05
**SNCA**	0.175	enzyme		1.116	1.17E-05
**etoposide**		chemical drug		1.037	1.20E-05
**MAPK1**	-0.118	kinase		0.000	1.23E-05
**FSH**		complex		1.733	1.61E-05
**CDC27**	-0.057	other			1.94E-05
**trans-hydroxytamoxifen**		chemical drug		-0.905	1.96E-05
**ASAH1**	0.639	enzyme		-1.231	1.96E-05
**PRKAA1**	0.150	kinase		-1.126	2.00E-05
**AG490**		chemical - kinase inhibitor		0.115	2.13E-05
**NKX2-3**		transcription regulator		-0.624	2.22E-05
**MYBL2**	-1.186	transcription regulator	Inhibited	-2.179	2.32E-05
**cycloheximide**		chemical reagent		-1.130	2.49E-05
**glucocorticoid**		chemical drug		0.440	2.55E-05
**tributyltin**		chemical reagent		-1.890	2.57E-05
**lipopolysaccharide**		chemical drug	Activated	2.130	3.12E-05

**MED1**	0.006	transcription regulator		-1.238	3.49E-05
**medroxyprogesterone acetate**		chemical drug	Activated	2.985	3.60E-05
**F2**	-0.708	peptidase		1.828	3.82E-05
**CCAR1**	-0.178	transcription regulator			3.84E-05
**CTNNB1**	-0.063	transcription regulator		0.789	4.07E-05
**bleomycin**		chemical drug		-1.019	4.09E-05
**RBL2**	0.246	other	Activated	2.382	4.19E-05
**progesterone**		chemical - endogenous mammalian		0.734	4.25E-05
**bucladesine**		chemical toxicant	Activated	2.306	4.69E-05
**GPER1**	-1.087	G-protein coupled receptor		1.673	4.72E-05
**FSHR**	3.080	G-protein coupled receptor		1.387	4.72E-05
**TFAP2A**	0.081	transcription regulator		1.545	4.83E-05
**NR3C2**	0.420	ligand-dependent nuclear receptor		0.277	4.87E-05
**poly rI:rC-RNA**		biologic drug		1.084	5.04E-05
**imipramine blue**		chemical drug	Activated	2.186	5.42E-05
**Creb**		group		1.219	5.76E-05
**L-methionine**		chemical - endogenous mammalian			5.87E-05
**CG**		complex	Activated	2.069	6.03E-05
**EWSR1**	-0.096	other		0.447	6.30E-05
**BMP2**		growth factor		1.078	6.41E-05
**KDM1A**	-0.068	enzyme	Inhibited	-2.417	6.47E-05
**BNIP3L**	0.478	other	Activated	2.449	6.52E-05
**SLC5A8**		transporter			6.66E-05
**APC (complex)**		complex			6.66E-05
**RBX1**	-0.413	enzyme			6.66E-05
**CD24**	0.432	other		-1.987	6.77E-05
**TGFB2**	0.624	growth factor		-0.445	7.23E-05
**plicamycin**		chemical drug		-0.355	7.71E-05
**ADRB**		group		-0.504	8.10E-05
**Tgf beta**		group		0.270	8.54E-05
**Lh**		complex		1.387	8.70E-05
**E2F3**	-0.330	transcription regulator	Inhibited	-2.138	9.05E-05
**mifepristone**		chemical drug		-0.199	9.15E-05
**D-glucose**		chemical - endogenous mammalian		1.321	9.26E-05
**GNA15**		enzyme			9.33E-05
**PGF**		growth factor			9.56E-05
**GDF9**	-0.293	growth factor		-0.638	9.56E-05
**troglitazone**		chemical drug		-0.154	1.01E-04
**NONO**	0.004	transcription regulator	Inhibited	-2.000	1.02E-04

**PD98059**		chemical - kinase inhibitor		0.637	1.06E-04
**FEV**		transcription regulator	Activated	2.646	1.07E-04
**SMARCE1**	0.047	transcription regulator			1.24E-04
**VEGFA**	-0.345	growth factor		0.977	1.32E-04
**RB1**	-0.159	transcription regulator		-0.359	1.35E-04
**TAL1**		transcription regulator	Inhibited	-2.530	1.35E-04
**WNT3A**		cytokine		0.437	1.35E-04
**Hif1**		complex		0.068	1.40E-04
**L-triiodothyronine**		chemical - endogenous mammalian		1.665	1.42E-04
**HDAC1**	-0.161	transcription regulator		-0.682	1.45E-04
**corticosterone**		chemical - endogenous mammalian		1.044	1.50E-04
**BMP15**		growth factor		-1.964	1.50E-04
**FSHB**		other		0.152	1.50E-04
**NR3C1**	-0.080	ligand-dependent nuclear receptor		0.406	1.52E-04
**gemcitabine**		chemical drug		-1.014	1.53E-04
**Sn50 peptide**		chemical toxicant		0.555	1.53E-04
**bazedoxifene**		chemical drug			1.57E-04
**prodigiosin**		chemical toxicant			1.57E-04
**FHL1**	-0.049	other			1.58E-04
**SLC8A1**	-1.559	transporter			1.58E-04
**1,1-bis(3'-indolyl)-1-(4-hydroxyphenyl)methane**		chemical reagent			1.58E-04
**4-(octyloxy)phenol**		chemical reagent			1.58E-04
**4-(heptyloxy)phenol**		chemical reagent			1.58E-04
**GNAQ**	0.371	enzyme			1.68E-04
**JAG1**	-0.215	growth factor		0.200	1.77E-04
**CRH**		cytokine		1.995	1.82E-04
**ESR2**	1.943	ligand-dependent nuclear receptor		0.638	1.90E-04
**EGR2**	-1.225	transcription regulator		1.408	1.92E-04
**RARA**	-0.470	ligand-dependent nuclear receptor		-1.342	1.99E-04
**nitric oxide**		chemical - endogenous mammalian		1.236	2.01E-04
**TREM1**		transmembrane receptor		-0.223	2.16E-04
**anacardic acid**		chemical - endogenous non-mammalian			2.22E-04
**SMOC2**		other			2.22E-04
**BMS-754807**		chemical drug			2.22E-04

**mir-21**		microRNA	Activated	3.464	2.41E-04
**bee venom**		chemical - endogenous non-mammalian	Activated	2.640	2.42E-04
**Irgm1**		other	Activated	2.219	2.47E-04
**Histone h3**		group			2.67E-04
**NS-398**		chemical reagent		-0.152	2.74E-04
**Hdac**		group	Inhibited	-2.099	2.76E-04
**let-7**		microRNA		1.440	2.86E-04
**dihydrotestosterone**		chemical - endogenous mammalian		0.160	2.91E-04
**PTHLH**	-0.438	other		1.067	2.94E-04
**Pka catalytic subunit**		group		1.941	2.95E-04
**HAND1**		transcription regulator		1.000	2.95E-04
**FGF9**		growth factor	Activated	2.000	2.95E-04
**PBRM1**	-0.117	other			3.02E-04
**IGF2**		growth factor			3.15E-04
**Calcineurin protein(s)**		complex		0.328	3.17E-04
**BMP4**	0.059	growth factor		1.443	3.21E-04
**estrogen**		chemical drug		-0.533	3.22E-04
**panobinostat**		chemical drug		-1.067	3.36E-04
**phenylephrine**		chemical drug		1.478	3.41E-04
**AMH**	-0.059	growth factor		-1.103	3.43E-04
**NR5A2**	-0.160	ligand-dependent nuclear receptor		1.652	3.49E-04
**JUN**	-0.939	transcription regulator		-0.504	3.62E-04
**INHA**	2.440	growth factor		0.570	3.70E-04
**TBX2**	1.543	transcription regulator		-1.633	3.94E-04
**TLX1**		transcription regulator		-1.000	3.97E-04
**cucurbitacin B**		chemical - endogenous non-mammalian			3.99E-04
**gefitinib**		chemical drug		1.981	4.06E-04
**5-N-ethylcarboxamido adenosine**		chemical reagent		1.344	4.15E-04
**SMARCA4**	-0.017	transcription regulator		1.155	4.19E-04
**nifedipine**		chemical drug		-0.447	4.23E-04
**IGF1**		growth factor		0.010	4.25E-04
**SHH**		peptidase		-0.652	4.60E-04
**PEPCK**		group			4.70E-04
**AZD7762**		chemical drug			4.70E-04
**CDC20**	-1.696	other			4.70E-04
**WTAP**	-0.268	other			4.70E-04
**methoxyacetic acid**		chemical toxicant			4.70E-04
**bisindolylmaleimide I**		chemical - kinase inhibitor		-1.195	5.18E-04
**S-nitroso-N-acetyl-DL-penicillamine**		chemical reagent		0.927	5.21E-04

**CASP3**	-0.106	peptidase		0.187	5.21E-04
**STAT3**	0.414	transcription regulator		1.008	5.28E-04
**Histone h4**		group			5.89E-04
**MBD3**	-0.124	other			5.93E-04
**butyric acid**		chemical - endogenous mammalian		1.302	6.12E-04
**fulvestrant**		chemical drug	Activated	2.005	6.24E-04
**KLF4**	0.773	transcription regulator		0.681	6.24E-04
**NAE1**	-0.401	enzyme			6.48E-04
**pyruvic acid**		chemical - endogenous mammalian			6.48E-04
**NGF**	-0.940	growth factor		0.703	6.59E-04
**NFYB**	0.281	transcription regulator			6.64E-04
**KDM4C**	-0.268	enzyme			6.70E-04
**glucagon**		biologic drug	Activated	2.236	6.90E-04
**MAPK14**	0.214	kinase	Activated	2.779	7.06E-04
**nicotine**		chemical drug		-0.158	7.33E-04
**CLEC4G**		other		0.447	7.48E-04
**GNRH1**	-0.454	other		-0.266	7.48E-04
**SB203580**		chemical - kinase inhibitor		-1.966	7.83E-04
**INHBA**	1.257	growth factor		1.335	7.90E-04
**miR-143-3p (and other miRNAs w/seed GAGAUGA)**		mature microRNA			8.03E-04
**PDE5A**	0.118	enzyme			8.03E-04
**nocodazole**		chemical reagent		0.072	8.12E-04
**WT1**		transcription regulator		-1.128	8.31E-04
**ADCYAP1**		other		1.258	8.58E-04
**salmonella minnesota R595 lipopolysaccharides**		chemical - endogenous non-mammalian		1.718	8.66E-04
**RUNX2**	0.766	transcription regulator		-0.379	8.66E-04
**isoproterenol**		chemical drug		1.371	9.03E-04
**ZFP36**	0.537	transcription regulator	Activated	2.449	9.15E-04
**Cmtm2a**		transcription regulator			9.32E-04
**SP6**		transcription regulator			9.32E-04
**ARTN**	-1.597	growth factor			9.32E-04
**methyl cellosolve**		chemical reagent			9.32E-04
**SP1**	0.058	transcription regulator		-0.031	9.44E-04
**epothilone B**		chemical drug			9.79E-04
**HIF1AN**	0.044	enzyme			9.79E-04
**MDK**	-0.460	growth factor			9.79E-04

**cisplatin**		chemical drug		0.345	9.99E-04
**E2f**		group		-1.980	1.00E-03
**LIPE**	-0.642	enzyme			1.00E-03
**TCF**		group			1.03E-03
**indomethacin**		chemical drug		0.306	1.10E-03
**WNT5A**	1.069	cytokine	Inhibited	-2.628	1.15E-03
**Hmgb1**		transcription regulator			1.18E-03
**DDX25**		enzyme			1.18E-03
**LATS2**	0.136	kinase			1.18E-03
**P2RY2**		G-protein coupled receptor			1.18E-03
**ATF6**	-0.013	transcription regulator	Inhibited	-2.219	1.18E-03
**uric acid**		chemical - endogenous mammalian	Activated	2.000	1.22E-03
**BMP7**		growth factor		-0.302	1.26E-03
**TNFSF13B**	0.665	cytokine			1.27E-03
**PTGS2**	1.253	enzyme		0.198	1.29E-03
**NFkB (complex)**		complex		0.196	1.31E-03
**RELA**	0.103	transcription regulator		1.510	1.32E-03
**docetaxel**		chemical drug		0.131	1.32E-03
**FOXO1**	1.412	transcription regulator		-1.067	1.35E-03
**Salmonella enterica serotype abortus equi lipopolysaccharide**		chemical toxicant		1.414	1.39E-03
**HCAR2**		G-protein coupled receptor			1.40E-03
**KLK4**		peptidase			1.40E-03
**AKR1B1**	0.043	enzyme			1.40E-03
**indirubin**		chemical drug			1.40E-03
**thiazolidinedione**		chemical drug			1.40E-03
**methylselenic acid**		chemical reagent			1.41E-03
**fluvastatin**		chemical drug			1.43E-03
**norepinephrine**		chemical - endogenous mammalian		0.874	1.44E-03
**NFKBIA**	-0.323	transcription regulator		0.307	1.47E-03
**raloxifene**		chemical drug		-1.134	1.50E-03
**darusentan**		chemical drug			1.54E-03
**acyline**		biologic drug			1.54E-03
**HSD17B1**	0.214	enzyme			1.54E-03
**Mamld1**		other			1.54E-03
**scoparone**		chemical - endogenous non-mammalian			1.54E-03
**atrasentan**		chemical drug			1.54E-03
**RXRA**	-0.248	ligand-dependent nuclear receptor		-0.798	1.55E-03
**NR5A1**	0.264	ligand-dependent	Activated	2.224	1.56E-03

		nuclear receptor			
**GHRL**		growth factor		-0.807	1.56E-03
**CIITA**	0.775	transcription regulator		1.993	1.57E-03
**carbamylcholine**		chemical drug		0.277	1.57E-03
**fluticasone propionate**		chemical drug		-0.863	1.61E-03
**simvastatin**		chemical drug		0.530	1.62E-03
**VHL**	-0.173	transcription regulator			1.64E-03
**HDAC4**	0.282	transcription regulator			1.64E-03
**NUMB**	-0.385	other			1.65E-03
**ACTB**	-0.211	other			1.65E-03
**RARRES2**	0.345	transmembrane receptor			1.65E-03
**leuprolide**		biologic drug			1.65E-03
**PIK3CA**	0.166	kinase		-0.068	1.67E-03
**LDL**		complex		1.164	1.72E-03
**PRKAA**		group		-0.124	1.73E-03
**TLR7/8**		group		-1.067	1.73E-03
**genistein**		chemical drug		1.262	1.82E-03
**HTT**	0.003	transcription regulator		0.277	1.83E-03
**DMP1**		other		1.000	1.89E-03
**dacinostat**		chemical drug			1.92E-03
**PHB**	-0.359	transcription regulator			1.92E-03
**naloxone**		chemical drug			1.92E-03
**GLI3**	-0.295	transcription regulator			2.02E-03
**curcumin**		chemical drug		0.837	2.05E-03
**BRCA1**	-1.447	transcription regulator		-0.167	2.07E-03
**hydrocortisone**		chemical - endogenous mammalian		0.520	2.10E-03
**trichostatin A**		chemical drug		1.085	2.10E-03
**E2F2**	-0.919	transcription regulator	Inhibited	-2.000	2.15E-03
**GnRH-A**		chemical reagent		1.172	2.15E-03
**PPARA**	0.657	ligand-dependent nuclear receptor		-1.172	2.21E-03
**SRA1**	-0.094	transcription regulator			2.22E-03
**NR4A1**	0.466	ligand-dependent nuclear receptor		-0.344	2.27E-03
**RNF31**	0.366	enzyme		-0.600	2.28E-03
**SPTBN2**	-0.452	other			2.29E-03
**LHX6**		transcription regulator			2.29E-03
**G6PC**		phosphatase			2.29E-03
**CIMO**		chemical reagent			2.29E-03

**SQ 29548**		chemical reagent			2.29E-03
**fluphenazine**		chemical drug			2.29E-03
**GABA receptor**		complex			2.29E-03
**sparfosic acid**		chemical drug			2.29E-03
**NKX2-1**		transcription regulator			2.29E-03
**IGF1R**	0.860	transmembrane receptor		1.826	2.40E-03
**MDM2**	-0.056	transcription regulator		-0.832	2.42E-03
**thymoquinone**		chemical drug			2.44E-03
**CITED2**	0.483	transcription regulator			2.44E-03
**PRKACA**	-0.246	kinase			2.44E-03
**Ro31-8220**		chemical - kinase inhibitor		-1.067	2.44E-03
**thyroid hormone**		chemical - endogenous mammalian		-0.647	2.45E-03
**VIP**		other		1.432	2.47E-03
**Pkg**		group			2.55E-03
**DYRK1A**	-0.087	kinase			2.55E-03
**PTPN3**	-0.394	phosphatase			2.55E-03
**U0126**		chemical - kinase inhibitor		-0.256	2.64E-03
**SB 216763**		chemical toxicant		1.067	2.65E-03
**vincristine**		chemical drug			2.65E-03
**EGR1**	-0.161	transcription regulator	Inhibited	-2.376	2.71E-03
**RRP1B**	0.008	transcription regulator			2.71E-03
**POU4F1**		transcription regulator			2.71E-03
**ZNF217**	0.203	transcription regulator		-1.000	2.72E-03
**H89**		chemical - kinase inhibitor		-1.091	2.75E-03
**streptozocin**		chemical drug		0.951	2.80E-03
**ADRA1B**	-0.046	G-protein coupled receptor			2.87E-03
**ADRA1D**		G-protein coupled receptor			2.87E-03
**dinoprost**		chemical - endogenous mammalian		0.399	2.88E-03
**Rb**		group			2.88E-03
**RBL1**	-1.350	transcription regulator			2.88E-03
**AICAR**		chemical - endogenous mammalian		-0.132	2.88E-03
**PRKCD**	-0.687	kinase		0.000	2.89E-03
**NOS2**		enzyme		1.067	2.89E-03
**Insulin**		group		0.046	2.89E-03

**PD184352**		chemical drug			2.90E-03
**CEBPB**	0.701	transcription regulator		1.611	2.95E-03
**losartan potassium**		chemical drug		-1.387	2.96E-03
**PRKCE**	0.249	kinase		0.327	3.05E-03
**HIF1A**	0.604	transcription regulator		0.908	3.07E-03
**RDH10**	-0.216	enzyme			3.18E-03
**ASCL2**		transcription regulator			3.18E-03
**HCRT**		other			3.18E-03
**ABCC4**	1.291	transporter			3.18E-03
**FERMT1**	-0.688	other			3.18E-03
**PROK1**		growth factor			3.18E-03
**casticin**		chemical - endogenous non-mammalian			3.18E-03
**DVL2**	-0.053	other			3.18E-03
**VGF**		growth factor			3.18E-03
**gliotoxin**		chemical toxicant			3.18E-03
**Muscarinic cholinergic receptor**		group			3.18E-03
**androstenedione**		chemical - endogenous mammalian			3.18E-03
**DNMT3A**	0.042	enzyme		0.956	3.19E-03
**TGFB3**	0.513	growth factor		0.970	3.24E-03
**PRL**		cytokine		0.736	3.27E-03
**GATA6**	0.052	transcription regulator		-0.384	3.28E-03
**risperidone**		chemical drug			3.29E-03
**Ikb**		group			3.29E-03
**UCN**	-0.082	other			3.29E-03
**DRAP1**	-0.694	transcription regulator			3.29E-03
**tetrachlorodibenzodioxin**		chemical toxicant		-0.225	3.33E-03
**RETN**		other		1.015	3.34E-03
**TEAD2**	-0.194	transcription regulator			3.34E-03
**IKBKB**	-0.416	kinase	Activated	2.578	3.35E-03
**melatonin**		chemical - endogenous mammalian		-1.172	3.38E-03
**RETNLB**		other		-0.447	3.40E-03
**TGFA**	0.122	growth factor		0.831	3.59E-03
**ADRA1A**		G-protein coupled receptor			3.59E-03
**kainic acid**		chemical toxicant		0.896	3.69E-03
**UCN-01**		chemical drug			3.70E-03
**MTPN**	0.213	transcription regulator		-0.555	3.78E-03
**cobalt chloride**		chemical reagent		-0.039	3.78E-03
**NRAS**	-0.113	enzyme			3.83E-03
**CBX5**	-0.382	transcription regu-		-0.447	3.84E-03

		lator		
**CDKN1B**	0.270	kinase	-0.084	3.84E-03
**cholecalciferol**		chemical - endogenous mammalian	1.595	3.84E-03
**PML**	-0.126	transcription regulator	0.954	4.09E-03
**atrazine**		chemical toxicant		4.21E-03
**NELFCD**	0.100	other		4.21E-03
**SULT2B1**		enzyme		4.21E-03
**S100a7a**		other		4.21E-03
**NUP155**	-0.709	transporter		4.21E-03
**DES**		other		4.21E-03
**DIABLO**	0.227	other		4.21E-03
**dantrolene**		chemical drug		4.21E-03
**raclopride**		chemical drug		4.21E-03
**alpha-tocopherol succinate**		chemical drug		4.21E-03
**N,N-dimethylarginine**		chemical - endogenous mammalian		4.21E-03
**resveratrol**		chemical drug	0.789	4.27E-03
**3M-002**		chemical reagent	0.000	4.34E-03
**E2F1**	-0.878	transcription regulator	-0.854	4.39E-03
**SMO**	0.128	G-protein coupled receptor		4.41E-03
**PTPRJ**	0.753	phosphatase	1.000	4.44E-03
**ARNT**	0.025	transcription regulator		4.52E-03
**IL1**		group	1.061	4.53E-03
**SP600125**		chemical - kinase inhibitor	0.263	4.59E-03
**CPE**	0.941	peptidase		4.62E-03
**isoflurane**		chemical drug		4.62E-03
**GNAS**	-0.202	enzyme		4.75E-03
**SAFB**	-0.171	other		4.75E-03
**INSR**	0.580	kinase	1.980	4.80E-03
**TWIST1**	-0.532	transcription regulator	1.134	4.84E-03
**MAPK9**	-0.349	kinase	0.896	4.84E-03
**chenodeoxycholic acid**		chemical - endogenous mammalian	1.446	4.87E-03
**CCL2**		cytokine	0.271	4.87E-03
**NFYA**	-0.126	transcription regulator		4.87E-03
**leukotriene D4**		chemical - endogenous mammalian	1.131	5.07E-03
**NLRP3**	0.989	other	1.109	5.11E-03
**LEP**		growth factor	-0.470	5.12E-03
**PRKAR2B**	0.267	kinase		5.12E-03
**titanium dioxide**		chemical drug		5.12E-03
**SMAD3**	-0.355	transcription regulator	0.479	5.33E-03

**garcinol**		chemical - endogenous non-mammalian		-0.447	5.36E-03
**novobiocin**		chemical drug			5.36E-03
**Mir218**		microRNA			5.36E-03
**Pde**		group			5.36E-03
**bendamustine**		chemical drug			5.36E-03
**SAV1**	-0.304	other			5.36E-03
**SERPINB2**		other			5.36E-03
**NELFA**	-0.122	other			5.36E-03
**MC1R**	0.435	G-protein coupled receptor			5.36E-03
**SRD5A1**	-0.442	enzyme			5.36E-03
**NELFE**	-0.200	other			5.36E-03
**benazepril**		chemical drug			5.36E-03
**BIRC5**	-1.547	other			5.40E-03
**EBI3**		cytokine		1.067	5.40E-03
**CHUK**	0.003	kinase	Activated	2.158	5.58E-03
**hydrogen peroxide**		chemical - endogenous mammalian		1.339	5.58E-03
**S100A6**	-0.280	transporter			5.66E-03
**letrozole**		chemical drug			5.66E-03
**NOTCH3**	0.672	transcription regulator			5.76E-03
**luteolin**		chemical drug			5.76E-03
**RASSF1**	-0.751	other	Activated	2.200	5.89E-03
**KLF11**	-0.514	transcription regulator			5.91E-03
**MAPK8**	0.084	kinase		1.452	6.06E-03
**pioglitazone**		chemical drug		-1.351	6.06E-03
**CCL11**		cytokine		-1.977	6.12E-03
**EGFR**	0.108	kinase		-0.351	6.16E-03
**SLC13A1**		transporter		1.342	6.16E-03
**vinblastine**		chemical drug			6.23E-03
**THRB**	0.122	ligand-dependent nuclear receptor			6.39E-03
**LIF**	0.228	cytokine		0.426	6.39E-03
**emodin**		chemical drug		0.577	6.50E-03
**CSF1**	-0.590	cytokine		-1.942	6.57E-03
**6-mercaptopurine**		chemical drug			6.65E-03
**O6-benzylguanine**		chemical drug			6.65E-03
**niclosamide**		chemical drug			6.65E-03
**PF-3084014**		chemical drug			6.65E-03
**RANBP9**	0.007	other			6.65E-03
**TFR2**		transporter			6.65E-03
**SIK1/SIK1B**	1.156	kinase			6.65E-03
**premarin**		chemical drug			6.65E-03
**farglitazar**		chemical drug			6.65E-03
**levamisole**		chemical drug			6.65E-03
**PDLIM2**	-0.069	other		-0.277	6.74E-03
**miR-146a-5p (and other miRNAs w/seed**		mature microRNA		1.342	6.74E-03

**GAGAACU)**					
**FGFR1**	-0.510	kinase		-1.000	6.74E-03
**mir-223**		microRNA		1.387	6.84E-03
**SRSF1**	-0.235	other			6.84E-03
**PRKAR1A**	0.279	kinase			6.84E-03
**betulinic acid**		chemical drug			6.84E-03
**vorinostat**		chemical drug		1.269	6.96E-03
**Ca2+**		chemical - endogenous mammalian		0.878	6.96E-03
**PPIF**	-0.121	enzyme		-1.342	7.04E-03
**HDAC2**	-0.126	transcription regulator		1.000	7.34E-03
**ATF3**	-0.977	transcription regulator		0.594	7.35E-03
**grape seed extract**		chemical drug			7.48E-03
**NPY**	1.834	other			7.48E-03
**NOSTRIN**	1.067	transcription regulator			7.48E-03
**olanzapine**		chemical drug		-0.365	7.68E-03
**Ifn gamma**		complex		1.446	8.01E-03
**C9orf72**	0.079	other			8.06E-03
**vismodegib**		chemical drug			8.06E-03
**TCF21**		transcription regulator			8.06E-03
**TBX1**		transcription regulator			8.06E-03
**HLF**		transcription regulator			8.06E-03
**S100A7**		other			8.06E-03
**STMN1**	-0.915	other			8.06E-03
**CA074-methyl ester**		chemical reagent			8.06E-03
**puerarin**		chemical drug			8.06E-03
**canrenoate potassium**		chemical drug			8.06E-03
**IKBKG**	0.021	kinase	Activated	2.425	8.14E-03
**NRG1**	0.453	growth factor		1.421	8.50E-03
**topotecan**		chemical drug		0.239	8.66E-03
**STAT5a/b**		group		-0.757	8.70E-03
**epigallocatechin-gallate**		chemical drug		0.674	8.72E-03
**ZBED6**	0.175	transcription regulator			8.86E-03
**PAK1**	-0.567	kinase			8.86E-03
**alvocidib**		chemical drug			8.86E-03
**15-deoxy-delta-12,14 -PGJ 2**		chemical - endogenous mammalian		-0.349	8.91E-03
**HRAS**	-0.035	enzyme		-1.485	9.04E-03
**12-(3-adamantan-1-yl-ureido) dodecanoic acid**		chemical reagent		0.577	9.13E-03
**folic acid**		chemical - endogenous mammalian		0.218	9.13E-03
**PGR**	0.181	ligand-dependent nuclear receptor		1.910	9.14E-03

**ionomycin**		chemical reagent		0.118	9.16E-03
**PTEN**	0.153	phosphatase		1.856	9.17E-03
**2-amino-5-phosphonovaleric acid**		chemical - other	Inhibited	-2.433	9.30E-03
**prednisolone**		chemical drug	Activated	2.121	9.39E-03
**embelin**		chemical - endogenous non-mammalian			9.59E-03
**WP1066**		chemical drug			9.59E-03
**WWC1**	0.748	transcription regulator			9.59E-03
**RGS2**	1.819	other			9.59E-03
**PRAME**	-0.124	other			9.59E-03
**HSF4**	0.211	transcription regulator			9.59E-03
**CDH5**		other			9.59E-03
**quinacrine**		chemical drug			9.59E-03
**nordihydroguaiaretic acid**		chemical drug			9.59E-03
**CYP27B1**	-0.002	enzyme			9.60E-03
**sildenafil**		chemical drug			9.60E-03
**sulindac**		chemical drug			9.62E-03
**cytarabine**		chemical drug			9.62E-03
**lovastatin**		chemical drug		-0.035	9.92E-03
**HAND2**	0.522	transcription regulator			1.01E-02
**SMARCA2**	0.029	transcription regulator			1.01E-02
**sodium chloride**		chemical - endogenous mammalian		1.995	1.01E-02
**NFATC1**	-0.411	transcription regulator		-0.061	1.02E-02
**PDX1**		transcription regulator		-1.000	1.02E-02
**ethanol**		chemical - endogenous mammalian		-0.497	1.03E-02
**8-bromoguanosine 3',5'-cyclic monophosphate**		chemical - kinase inhibitor			1.04E-02
**IL27**		cytokine	Activated	2.391	1.06E-02
**IL15**	0.308	cytokine		0.858	1.06E-02
**estradiol benzoate**		chemical drug			1.07E-02
**camptothecin**		chemical drug		-1.255	1.08E-02
**CCN2**	0.329	growth factor		1.067	1.10E-02
**Akt**		group		-1.231	1.11E-02
**STAT5A**	0.995	transcription regulator			1.11E-02
**bortezomib**		chemical drug		0.375	1.11E-02
**MSX2**	-0.942	transcription regulator			1.12E-02
**Yaf2**		transcription regulator			1.12E-02
**4-methylnitrosoamino-1-(3-pyridinyl)-1-butanone**		chemical toxicant			1.12E-02

**lipoteichoic acid**		chemical - endogenous non-mammalian			1.12E-02
**D-alpha-tocopheryl succinate**		chemical reagent			1.12E-02
**YWHAQ**	-0.361	other			1.12E-02
**CTSG**		peptidase			1.12E-02
**RBP1**	0.329	transporter			1.12E-02
**BHLHE41**	-0.526	transcription regulator			1.12E-02
**phencyclidine**		chemical drug			1.12E-02
**hormone**		chemical drug			1.12E-02
**maxacalcitol**		chemical drug			1.12E-02
**estrogen receptor**		group		-0.113	1.14E-02
**SMAD4**	-0.013	transcription regulator		-0.762	1.14E-02
**bexarotene**		chemical drug		0.174	1.14E-02
**thapsigargin**		chemical toxicant		0.033	1.14E-02
**IFNG**		cytokine	Activated	2.018	1.14E-02
**CREBBP**	-0.151	transcription regulator			1.15E-02
**MAP2K1**	0.081	kinase		-0.218	1.17E-02
**mir-34**		microRNA		1.924	1.18E-02
**AKT1**	-0.157	kinase			1.18E-02
**fatty acid**		chemical - endogenous mammalian		1.951	1.19E-02
**NQO1**	-0.014	enzyme			1.20E-02
**mevalonic acid**		chemical - endogenous mammalian			1.20E-02
**CEBPA**	-0.450	transcription regulator		0.680	1.23E-02
**GATA1**		transcription regulator		0.000	1.26E-02
**endocannabinoid**		chemical - endogenous mammalian			1.26E-02
**ZYG11B**	0.076	other			1.26E-02
**Ca2 ATPase**		group			1.26E-02
**Hdac1/2**		group			1.26E-02
**SNHG1**	-0.570	other			1.26E-02
**mecarzole**		chemical drug			1.26E-02
**sodium thiosulfate**		chemical drug			1.26E-02
**CYP2R1**	-0.326	enzyme			1.26E-02
**FBXL7**	0.069	enzyme			1.26E-02
**HJURP**	-1.722	other			1.26E-02
**DNAJA4**	-0.671	other			1.26E-02
**RGN**		enzyme			1.26E-02
**CD3-TCR**		complex			1.26E-02
**SNHG7**	0.002	other			1.26E-02
**ASPSCR1**	0.061	other			1.26E-02
**MARCH6**	0.333	enzyme			1.26E-02
**SGMS2**	-0.340	enzyme			1.26E-02
**linsitinib**		chemical drug			1.26E-02

**HSPA6**		enzyme	1.26E-02
**ASB7**	0.342	other	1.26E-02
**JPH2**	-0.697	enzyme	1.26E-02
**SIRT5**	0.172	enzyme	1.26E-02
**METAP2**	-0.169	peptidase	1.26E-02
**ZYG11A**	-1.117	other	1.26E-02
**amuvatinib**		chemical drug	1.26E-02
**proprotein convertase**		group	1.26E-02
**voxtalisib**		chemical drug	1.26E-02
**TNNC1**		other	1.26E-02
**NAPA**	0.124	transporter	1.26E-02
**miR-302b-5p (and other miRNAs w/seed CUUUAAC)**		mature microRNA	1.26E-02
**miR-381-3p (and other miRNAs w/seed AUACAAG)**		mature microRNA	1.26E-02
**miR-181a-1-3p (and other miRNAs w/seed CCAUCGA)**		mature microRNA	1.26E-02
**mir-463**		microRNA	1.26E-02
**mir-340**		microRNA	1.26E-02
**mir-471**		microRNA	1.26E-02
**DYNLT3**	0.314	other	1.26E-02
**SCD5**	0.091	enzyme	1.26E-02
**KCNA1**		ion channel	1.26E-02
**TNR**		other	1.26E-02
**NUDT21**	-0.566	other	1.26E-02
**DSG2**	-0.048	other	1.26E-02
**RIT1**	-0.031	enzyme	1.26E-02
**TBXAS1**	-0.933	enzyme	1.26E-02
**MPT0B214**		chemical reagent	1.26E-02
**CDC23**	-0.374	enzyme	1.26E-02
**ITIH4**		other	1.26E-02
**SIRT1/2 inhibitor VII**		chemical reagent	1.26E-02
**TPD52**	0.426	other	1.26E-02
**ALYREF**	-0.718	transcription regulator	1.26E-02
**COX7A2**	-0.057	enzyme	1.26E-02
**HMGN5**	0.426	transcription regulator	1.26E-02
**pharmacoperone IN3**		chemical reagent	1.26E-02
**IGHMBP2**	0.082	enzyme	1.26E-02
**SR11256**		chemical reagent	1.26E-02
**CWP232228**		chemical reagent	1.26E-02
**chlordane**		chemical toxicant	1.26E-02
**proadifen**		chemical reagent	1.26E-02
**guanethidine**		chemical drug	1.26E-02
**darifenacin**		chemical drug	1.26E-02
**2,5-dihydroxymethylcinnamate**		chemical - kinase inhibitor	1.26E-02
**benomyl**		chemical toxicant	1.26E-02
**EPI-002**		chemical reagent	1.26E-02
**melarsoprol**		chemical drug	1.26E-02

**trichlorfon**		chemical toxicant		1.26E-02
**fadrozole**		chemical drug		1.26E-02
**hedamycin**		chemical reagent		1.26E-02
**gallamine triethiodide**		chemical drug		1.26E-02
**AuNP@PEG@e14a2**		chemical reagent		1.26E-02
**BMS200261**		chemical reagent		1.26E-02
**Gly-Gln**		chemical reagent		1.26E-02
**2,3-dichloro-5,8-dihydroxy-1,4-naphthoquinone**		chemical toxicant		1.26E-02
**alcuronium**		chemical drug		1.26E-02
**becaplermin**		biologic drug		1.26E-02
**hydrocortisone phosphate**		chemical drug		1.26E-02
**6alpha-fluorotestosterone**		chemical toxicant		1.26E-02
**PI3K (complex)**		complex	-0.822	1.28E-02
**ATP7B**	0.907	transporter		1.29E-02
**NR2F1**	-0.528	ligand-dependent nuclear receptor		1.29E-02
**cyclopamine**		chemical reagent		1.29E-02
**GNA14**		enzyme		1.30E-02
**miR-141-3p (and other miRNAs w/seed AACACUG)**		mature microRNA	-0.061	1.30E-02
**tyrphostin AG 1478**		chemical - kinase inhibitor	-0.057	1.30E-02
**PKD1**	0.141	ion channel	-1.664	1.30E-02
**GON4L**	-0.010	transcription regulator		1.30E-02
**Ptprd**		phosphatase		1.30E-02
**PECAM1**	-0.669	other		1.30E-02
**Betacatenin/TCF**		complex		1.30E-02
**NDUFA13**	-0.035	enzyme		1.30E-02
**IQGAP2**	-0.412	other		1.30E-02
**TGFBR3**	-0.384	kinase		1.30E-02
**cetrorelix**		biologic drug		1.30E-02
**aphidicolin**		chemical toxicant		1.30E-02
**selenite**		chemical toxicant		1.30E-02
**zeranol**		chemical - endogenous mammalian		1.30E-02
**noscapine**		chemical drug		1.30E-02
**growth factor**		group		1.30E-02
**onapristone**		chemical drug		1.30E-02
**SOX1**		transcription regulator	0.000	1.36E-02
**tacrolimus**		chemical drug	-0.000	1.36E-02
**EDN1**	1.834	cytokine	0.650	1.36E-02
**CEACAM1**		transporter		1.38E-02
**DACH1**	0.208	transcription regulator		1.38E-02
**FLT3**		kinase		1.38E-02
**LG100268**		chemical reagent		1.38E-02
**REST**	-0.046	transcription regulator	-1.032	1.39E-02

**black raspberry extract**		chemical drug		1.42E-02
**hyaluronic acid**		chemical - endogenous mammalian	-0.520	1.47E-02
**NR0B1**		ligand-dependent nuclear receptor		1.48E-02
**benzyl isothiocyanate**		chemical - endogenous non-mammalian		1.48E-02
**Bvht**		other	1.000	1.49E-02
**NTRK2**		kinase		1.49E-02
**lonafarnib**		chemical drug		1.49E-02
**1'-acetoxychavicol acetate**		chemical reagent		1.49E-02
**miR-19b-3p (and other miRNAs w/seed GUGCAAA)**		mature microRNA		1.49E-02
**mir-199**		microRNA		1.49E-02
**mir-154**		microRNA		1.49E-02
**RFX5**	0.211	transcription regulator		1.49E-02
**perindopril**		chemical drug		1.49E-02
**pyridoxine**		chemical - endogenous mammalian		1.49E-02
**asiatic acid**		chemical reagent		1.49E-02
**24-hydroxycholesterol**		chemical - endogenous mammalian		1.49E-02
**MAP2K6**	0.190	kinase		1.55E-02
**triptolide**		chemical drug		1.55E-02
**epinephrine**		chemical - endogenous mammalian		1.55E-02
**NAMPT**	0.274	cytokine		1.58E-02
**CD2**		transmembrane receptor		1.58E-02
**NFYC**	-0.108	transcription regulator		1.58E-02
**NKX2-5**		transcription regulator		1.58E-02
**HOXC6**	-0.042	transcription regulator		1.58E-02
**testosterone**		chemical - endogenous mammalian	1.569	1.61E-02
**paclitaxel**		chemical drug	0.330	1.62E-02
**PD173074**		chemical reagent	1.000	1.62E-02
**Notch**		group		1.62E-02
**AHR**	0.290	ligand-dependent nuclear receptor	0.861	1.63E-02
**isobutylmethylxanthine**		chemical toxicant	1.812	1.66E-02
**ACVR1**	-0.009	kinase		1.68E-02
**GABPA**	0.024	transcription regulator		1.68E-02
**GSK2816126**		chemical drug		1.68E-02
**CDK2AP1**	-0.237	other		1.68E-02
**baicalein**		chemical drug		1.68E-02

**CGB3 (includes others)**		other			1.69E-02
**CHEK2**	-0.275	kinase			1.69E-02
**NKX6-1**	-0.861	transcription regulator			1.69E-02
**HDAC9**	-0.294	transcription regulator			1.69E-02
**CBX3**	-0.228	transcription regulator			1.69E-02
**aroclor 1254**		chemical toxicant			1.69E-02
**4-nitroquinoline-1-oxide**		chemical toxicant			1.69E-02
**cystamine**		chemical drug			1.69E-02
**27-hydroxycholesterol**		chemical - endogenous mammalian			1.69E-02
**staurosporine**		chemical - kinase inhibitor		1.446	1.69E-02
**oleic acid**		chemical - endogenous mammalian		-1.432	1.69E-02
**ROR2**	0.256	kinase			1.69E-02
**IgG**		complex		-0.697	1.74E-02
**VCAN**	0.527	other		0.000	1.74E-02
**EIF4E**	-0.132	translation regulator		-1.342	1.74E-02
**1,4-bis[2-(3,5-dichloropyridyloxy)]benzene**		chemical toxicant		-0.322	1.74E-02
**SP2509**		chemical reagent	Activated	3.000	1.75E-02
**SOX2**		transcription regulator		1.067	1.78E-02
**ANGPT1**	1.770	growth factor			1.79E-02
**ILK**	-0.200	kinase			1.79E-02
**advanced glycation end-products**		chemical - endogenous mammalian			1.79E-02
**MECP2**	0.115	transcription regulator			1.80E-02
**metformin**		chemical drug	Inhibited	-2.200	1.83E-02
**MYC**	-0.071	transcription regulator	Inhibited	-2.976	1.84E-02
**SOX3**		transcription regulator		0.000	1.84E-02
**ciglitazone**		chemical drug			1.84E-02
**N-Ac-Leu-Leu-norleucinal**		chemical - protease inhibitor			1.84E-02
**R5020**		chemical reagent			1.84E-02
**methylprednisolone**		chemical drug		1.930	1.86E-02
**AMPK**		complex		1.131	1.86E-02
**MYB**		transcription regulator		-0.839	1.88E-02
**cryptotanshinone**		chemical drug			1.90E-02
**Angiotensin II receptor type 1**		group			1.90E-02
**GIP**		other			1.90E-02
**MXI1**	0.357	transcription regulator			1.90E-02
**DAXX**	-0.402	transcription regu-			1.90E-02

		lator		
**IL19**		cytokine		1.90E-02
**actinonin**		chemical reagent		1.90E-02
**zearalenone**		chemical toxicant		1.90E-02
**K+**		chemical - endogenous mammalian		1.90E-02
**valproic acid**		chemical drug	0.743	1.91E-02
**celecoxib**		chemical drug	1.683	1.92E-02
**NOTCH1**	-0.130	transcription regulator	-0.304	1.95E-02
**Jnk**		group	0.342	1.99E-02
**spironolactone**		chemical drug	-1.000	2.00E-02
**SMAD1**	-0.239	transcription regulator		2.01E-02
**KAT6A**	-0.030	enzyme		2.01E-02
**ABCA1**	0.227	transporter		2.01E-02
**Foxp1**		transcription regulator		2.01E-02
**GMNN**	-1.018	transcription regulator	0.000	2.08E-02
**TRAF2**	-0.397	enzyme	-1.987	2.08E-02
**TFRC**	0.097	transporter		2.08E-02
**FGF1**	-1.644	growth factor	-0.669	2.11E-02
**Fgfr**		group		2.12E-02
**Pdgf Ab**		complex		2.12E-02
**hemoglobin**		complex		2.12E-02
**S100B**		other		2.12E-02
**CDK1**	-1.310	kinase		2.12E-02
**PLCG2**		enzyme		2.12E-02
**ARHGDIG**		other		2.12E-02
**ITGA6**	-0.430	transmembrane receptor		2.12E-02
**KLRC4-KLRK1/KLRK1**		transmembrane receptor		2.12E-02
**ACTN4**	-0.361	transcription regulator		2.12E-02
**NCR2**		transmembrane receptor		2.12E-02
**antimycin A**		chemical - endogenous non-mammalian		2.12E-02
**sertraline**		chemical drug		2.12E-02
**glycyrrhetinic acid**		chemical drug		2.12E-02
**pilocarpine**		chemical drug		2.12E-02
**Sb202190**		chemical - kinase inhibitor	-1.400	2.17E-02
**5-O-mycolyl-beta-araf-(1->2)-5-O-mycolyl-alpha-araf-(1->1')-glycerol**		chemical - endogenous non-mammalian	1.000	2.25E-02
**FGF19**		growth factor		2.25E-02
**lithium**		chemical drug	-1.992	2.25E-02

**TP63**		transcription regulator	-0.694	2.32E-02
**EPAS1**	-0.008	transcription regulator	0.051	2.32E-02
**Z-LLL-CHO**		chemical - protease inhibitor	-1.020	2.33E-02
**flutamide**		chemical drug		2.34E-02
**TLX3**		transcription regulator		2.35E-02
**TET3**	-0.549	enzyme		2.35E-02
**artesunic acid**		chemical drug		2.35E-02
**CCK**		other		2.35E-02
**miR-486-5p (and other miRNAs w/seed CCUGUAC)**		mature microRNA		2.35E-02
**GAS5**	-0.039	other		2.35E-02
**WWOX**	-0.291	enzyme		2.35E-02
**EPHA2**	-0.189	kinase		2.35E-02
**isoprenaline**		chemical drug		2.35E-02
**riboflavin**		chemical - endogenous mammalian		2.35E-02
**baicalin**		chemical - endogenous non-mammalian		2.38E-02
**ITGA5**	-0.374	transmembrane receptor		2.38E-02
**MAPK7**	-0.156	kinase		2.38E-02
**ERBB4**	0.533	kinase	-1.964	2.42E-02
**CSF3**		cytokine	-1.720	2.43E-02
**5,6-epoxyeicosatrienoic acid**		chemical - endogenous mammalian		2.50E-02
**(6E,8Z,11Z,14Z)-(5S)-5-hydroperoxyeicosa-6,8.11,14-tetraenoic acid**		chemical - endogenous mammalian		2.50E-02
**minoxidil**		chemical drug		2.50E-02
**4-aminopyrazolo(3,4-d)pyrimidine**		chemical reagent		2.50E-02
**purvalanol A**		chemical reagent		2.50E-02
**amikacin**		chemical drug		2.50E-02
**7beta-hydroxycholesterol**		chemical - endogenous mammalian		2.50E-02
**MICU1**	0.239	other		2.50E-02
**1,2-dibromo-3-chloropropane**		chemical toxicant		2.50E-02
**GST**		complex		2.50E-02
**vildagliptin**		chemical drug		2.50E-02
**AEOL-10150**		chemical drug		2.50E-02
**glemanserin**		chemical drug		2.50E-02
**macimorelin**		biologic drug		2.50E-02
**thymol**		chemical reagent		2.50E-02
**Rab11**		group		2.50E-02
**MTNR1A**		G-protein coupled receptor		2.50E-02
**USP15**	0.145	peptidase		2.50E-02
**PCDH9**	0.341	other		2.50E-02

**hyperoside**		chemical - endogenous non-mammalian	2.50E-02
**SENP8**	-0.549	peptidase	2.50E-02
**DENND1A**	0.263	other	2.50E-02
**alpha-adrenergic receptor**		group	2.50E-02
**CP**	0.359	enzyme	2.50E-02
**MTNR1B**		G-protein coupled receptor	2.50E-02
**TBX4**		transcription regulator	2.50E-02
**Hat**		complex	2.50E-02
**AHSA1**	0.017	other	2.50E-02
**soy isoflavones**		chemical drug	2.50E-02
**adrenoceptor**		group	2.50E-02
**CHIR-124**		chemical reagent	2.50E-02
**1-(3-C-ethynylribopentofuranosyl)cytosine**		chemical drug	2.50E-02
**LY 518674**		chemical drug	2.50E-02
**bucillamine**		chemical drug	2.50E-02
**STAT-1/2**		group	2.50E-02
**CACYBP**	-0.208	other	2.50E-02
**safflor yellow B**		chemical - endogenous non-mammalian	2.50E-02
**NOSIP**	-0.170	other	2.50E-02
**KCNJ5**		ion channel	2.50E-02
**NPBWR1**		G-protein coupled receptor	2.50E-02
**AFM**		transporter	2.50E-02
**DAPK3**	-0.112	kinase	2.50E-02
**HPGD**	0.664	enzyme	2.50E-02
**SYCP3**	0.493	other	2.50E-02
**miR-344d-3p (and other miRNAs w/seed AUAUAAC)**		mature microRNA	2.50E-02
**miR-292-3p (and other miRNAs w/seed AGUGCCG)**		mature microRNA	2.50E-02
**mir-370**		microRNA	2.50E-02
**mir-153**		microRNA	2.50E-02
**lynestrenol**		chemical drug	2.50E-02
**CCNG1**	0.005	other	2.50E-02
**HIF1A-AS1**	0.601	other	2.50E-02
**PIM**		group	2.50E-02
**ECE1**	0.018	peptidase	2.50E-02
**PFN2**	-0.096	enzyme	2.50E-02
**HIPK3**	0.364	kinase	2.50E-02
**PLIN3**	-0.119	other	2.50E-02
**MUC16**		other	2.50E-02
**cyclo(iso-Asp-GR)-LLIIKLAKLAKKLAKLAK**		chemical reagent	2.50E-02
**NTSR2**		G-protein coupled receptor	2.50E-02

**SVIL**	-0.317	other	2.50E-02
**TH**		enzyme	2.50E-02
**GPBP1**	-0.145	transcription regulator	2.50E-02
**PRIMA1**		other	2.50E-02
**PNRC1**	0.397	other	2.50E-02
**NEU2**		enzyme	2.50E-02
**UBE2V1**	-0.078	transcription regulator	2.50E-02
**Gm4836 (includes others)**		other	2.50E-02
**APOC1**	0.694	transporter	2.50E-02
**RFX4**		transcription regulator	2.50E-02
**VNN1**		enzyme	2.50E-02
**3830403N18Rik/Xlr**		other	2.50E-02
**securinine**		chemical reagent	2.50E-02
**gamma-secretase inhibitor XX**		chemical reagent	2.50E-02
**S7**		chemical - kinase inhibitor	2.50E-02
**SI163**		chemical - kinase inhibitor	2.50E-02
**S29**		chemical - kinase inhibitor	2.50E-02
**daphnoretin**		chemical - endogenous non-mammalian	2.50E-02
**satavaptan**		chemical drug	2.50E-02
**thimerosal**		chemical drug	2.50E-02
**XK469**		chemical drug	2.50E-02
**talipexole**		chemical drug	2.50E-02
**AN-207**		chemical toxicant	2.50E-02
**SSR180575**		chemical drug	2.50E-02
**SD-1008**		chemical - kinase inhibitor	2.50E-02
**lavendustin A**		chemical - kinase inhibitor	2.50E-02
**endomorphin-2**		chemical - endogenous mammalian	2.50E-02
**isoalantolactone**		chemical - endogenous non-mammalian	2.50E-02
**ingenol-dibenzoate**		chemical reagent	2.50E-02
**swainsonine**		chemical - endogenous non-mammalian	2.50E-02
**N-methylscopolamine**		chemical drug	2.50E-02
**3-keto, N-aminoethyl aminocaproyl dihydrocinnamoyl cyclopamine**		chemical reagent	2.50E-02
**22-hydroxycholesterol**		chemical - endogenous mammalian	2.50E-02
**RARG**	-0.010	ligand-dependent	2.52E-02

		nuclear receptor		
**TGFBR1**	0.615	kinase	1.172	2.52E-02
**APP**	0.340	other	1.228	2.53E-02
**mir-486**		microRNA		2.58E-02
**AXIN1**	0.067	other		2.58E-02
**GABPB1**	-0.136	transcription regulator		2.58E-02
**LY6E**	-0.120	other		2.58E-02
**NAB2**	0.002	transcription regulator		2.58E-02
**finasteride**		chemical drug		2.58E-02
**miR-291a-3p (and other miRNAs w/seed AAGUGCU)**		mature microRNA	0.854	2.61E-02
**methimazole**		chemical drug		2.64E-02
**9.10-dimethyl-1,2-benzanthracene**		chemical toxicant		2.64E-02
**cyclosporin A**		biologic drug	-0.785	2.76E-02
**POU5F1**		transcription regulator	0.025	2.80E-02
**phorbol esters**		chemical - other	-0.371	2.80E-02
**phosphate**		chemical - endogenous mammalian	-1.091	2.80E-02
**salinosporamide A**		chemical drug		2.83E-02
**DCAF1**	-0.017	kinase		2.83E-02
**PRDM16**	1.506	transcription regulator		2.83E-02
**XRCC6**	-0.304	enzyme		2.83E-02
**mir-26**		microRNA		2.83E-02
**SOCS6**	0.161	other		2.83E-02
**linalool**		chemical - endogenous non-mammalian		2.83E-02
**NFAT5**	0.021	transcription regulator	1.000	2.90E-02
**SYVN1**	0.455	transporter	-0.447	2.90E-02
**bromodeoxyuridine**		chemical drug		2.91E-02
**ADM**		other		2.91E-02
**salirasib**		chemical drug		2.91E-02
**n-nitrosomethylbenzylamine**		chemical toxicant		2.98E-02
**YBX1**	-0.304	transcription regulator		3.00E-02
**OGT**	0.251	enzyme	-1.000	3.00E-02
**IL5**		cytokine	-1.488	3.00E-02
**DETA-NONOate**		chemical reagent		3.06E-02
**PARPBP**	-0.910	other		3.09E-02
**EIF4G1**	-0.090	translation regulator		3.09E-02
**GADD45A**	-0.783	other		3.09E-02
**CCN3**	1.415	growth factor		3.09E-02
**GRN**	0.251	growth factor		3.09E-02
**IL15RA**	-0.558	transmembrane receptor		3.09E-02

**wogonin**		chemical - endogenous non-mammalian			3.09E-02
**CALCA**		other			3.11E-02
**KMT2A**	-0.010	transcription regulator			3.11E-02
**ITGB1**	0.263	transmembrane receptor		-0.786	3.15E-02
**quercetin**		chemical drug		0.757	3.15E-02
**CLEC11A**	0.102	growth factor			3.20E-02
**SIRT1**	-0.191	transcription regulator		1.480	3.27E-02
**bezafibrate**		chemical drug		-0.927	3.32E-02
**THPO**		cytokine			3.35E-02
**vitamin D**		chemical drug			3.35E-02
**pregna-4.17-diene-3.16-dione**		chemical - endogenous non-mammalian			3.36E-02
**FAM3B**		cytokine			3.36E-02
**oxymatrine**		chemical drug			3.36E-02
**MAP2K5**	0.463	kinase			3.36E-02
**CETP**		enzyme			3.36E-02
**desipramine**		chemical drug			3.36E-02
**evodiamine**		chemical - endogenous non-mammalian			3.36E-02
**diallyl trisulfide**		chemical - endogenous non-mammalian			3.36E-02
**CNR1**		G-protein coupled receptor		1.387	3.40E-02
**ritonavir**		chemical drug			3.40E-02
**aldosterone**		chemical - endogenous mammalian	Activated	2.128	3.40E-02
**BMP6**	0.397	growth factor		-0.659	3.43E-02
**MTOR**	0.056	kinase		0.640	3.44E-02
**TLR9**		transmembrane receptor		-0.179	3.47E-02
**ID2**	1.227	transcription regulator		0.577	3.50E-02
**MAPKAPK2**	0.272	kinase			3.51E-02
**CEBPD**	1.583	transcription regulator		0.192	3.54E-02
**zerumbone**		chemical - endogenous non-mammalian			3.63E-02
**TRPS1**	0.278	transcription regulator			3.63E-02
**MAP3K3**	0.038	kinase			3.63E-02
**LCAT**	0.151	enzyme			3.63E-02
**diosgenin**		chemical - endog-			3.63E-02

		enous non-mammalian			
**Pdgf (complex)**		complex		1.980	3.65E-02
**POMC**		other		1.923	3.65E-02
**MAP2K4**	-0.142	kinase			3.67E-02
**SCD**	0.309	enzyme			3.67E-02
**GW 4064**		chemical toxicant			3.67E-02
**CpG oligonucleotide**		chemical drug			3.67E-02
**thioguanine**		chemical drug			3.73E-02
**L-tyrosine**		chemical - endogenous mammalian			3.73E-02
**hyodeoxycholic acid**		chemical - endogenous mammalian			3.73E-02
**chloride**		chemical - endogenous mammalian			3.73E-02
**1-palmitoyl-2-arachidonoyl-phosphatidylcholine**		chemical - endogenous mammalian			3.73E-02
**ixabepilone**		chemical drug			3.73E-02
**Cyclin B**		group			3.73E-02
**lipoxygenase**		group			3.73E-02
**sethoxydim**		chemical toxicant			3.73E-02
**CFC1/CFC1B**		other			3.73E-02
**muraglitazar**		chemical drug			3.73E-02
**PRAG1**	-0.332	kinase			3.73E-02
**CADPS**		other			3.73E-02
**CLTRN**		other			3.73E-02
**FAM168A**	0.315	other			3.73E-02
**SOCS2-AS1**	-0.453	other			3.73E-02
**KLF14**		transcription regulator			3.73E-02
**LAPTM4B**	0.285	other			3.73E-02
**Integrin alpha 4 beta 1**		complex			3.73E-02
**STIL**	-1.140	other			3.73E-02
**EXOC3L1**	-0.627	other			3.73E-02
**CKLF**	-0.683	cytokine			3.73E-02
**Trk Receptor**		group			3.73E-02
**xenon**		chemical drug			3.73E-02
**defibrotide**		biologic drug			3.73E-02
**GEM231**		chemical drug			3.73E-02
**cerebrolysin**		chemical drug			3.73E-02
**RNF144B**	-0.456	enzyme			3.73E-02
**PAG1**	0.418	other			3.73E-02
**GSTA4**	-0.566	enzyme			3.73E-02
**ARID1B**	0.069	transcription regulator			3.73E-02
**MADD**	0.555	other			3.73E-02
**miR-136-5p (miRNAs w/seed CUCCAUU)**		mature microRNA			3.73E-02
**miR-376a-5p (miRNAs w/seed UAGAUUC)**		mature microRNA			3.73E-02
**mir-541**		microRNA			3.73E-02
**mir-379**		microRNA			3.73E-02
**miR-153-3p (miRNAs w/seed UGCAUAG)**		mature microRNA			3.73E-02

**mir-342**		microRNA	3.73E-02
**miR-224-5p (miRNAs w/seed AAGUCAC)**		mature microRNA	3.73E-02
**PYCR2**	-0.026	enzyme	3.73E-02
**SCN1B**	-0.103	ion channel	3.73E-02
**NRP2**	-0.041	kinase	3.73E-02
**BMP1**	-0.118	peptidase	3.73E-02
**N4BP1**	-0.169	other	3.73E-02
**S100A1**	-0.584	other	3.73E-02
**SOD3**	-0.160	enzyme	3.73E-02
**ANKRD1**	-0.763	transcription regulator	3.73E-02
**TRERF1**	0.332	transcription regulator	3.73E-02
**CCNL2**	-0.037	other	3.73E-02
**Cdkal1**		enzyme	3.73E-02
**MAP1S**	-0.225	enzyme	3.73E-02
**AP2A2**	0.038	transporter	3.73E-02
**EPHA4**	-0.519	kinase	3.73E-02
**VEZF1**	-0.169	transcription regulator	3.73E-02
**NR2F6**	0.000	ligand-dependent nuclear receptor	3.73E-02
**MT3**		other	3.73E-02
**Rbpjl2**		other	3.73E-02
**RPL10**	-0.022	translation regulator	3.73E-02
**BUB1B**	-1.945	kinase	3.73E-02
**IRX1**		transcription regulator	3.73E-02
**temocapril**		chemical reagent	3.73E-02
**nilutamide**		chemical drug	3.73E-02
**AA-861**		chemical reagent	3.73E-02
**glyceollin**		chemical - endogenous non-mammalian	3.73E-02
**DL-threo-dihydrosphingosine**		chemical - kinase inhibitor	3.73E-02
**fosbretabulin**		chemical drug	3.73E-02
**pramipexole**		chemical drug	3.73E-02
**pirenzepine**		chemical drug	3.73E-02
**ethyl-3-trifluoromethylbenzyl-alpha-ketoglutarate**		chemical reagent	3.73E-02
**4-amino-6-hydrazino-7-beta-D-ribofuranosyl-7H-pyrrolo[2,3-d]-pyrimidine-5-carboxamide**		chemical reagent	3.73E-02
**lead nitrate**		chemical toxicant	3.73E-02
**imisopasem manganese**		chemical drug	3.73E-02
**actinomycin**		chemical - endogenous non-mammalian	3.73E-02
**L-JNK inhibitor I**		chemical reagent	3.73E-02

**phosphoramidon**		chemical - endogenous non-mammalian		3.73E-02
**Z-LEHD-FMK**		chemical reagent		3.73E-02
**10-hydroxycamptothecin**		chemical - endogenous mammalian		3.73E-02
**physostigmine**		chemical drug		3.73E-02
**E64**		chemical - endogenous non-mammalian		3.73E-02
**D,L-propargylglycine**		chemical reagent		3.73E-02
**2R,4R-4-aminopyrrolidine-2,4-dicarboxylic acid**		chemical reagent		3.73E-02
**fosinopril**		chemical drug		3.73E-02
**megestrol acetate**		chemical drug		3.73E-02
**desogestrel**		chemical drug		3.73E-02
**TNFSF10**	1.578	cytokine	1.309	3.77E-02
**cephaloridine**		chemical drug	0.000	3.77E-02
**arachidonic acid**		chemical - endogenous mammalian	1.217	3.77E-02
**rosiglitazone**		chemical drug	-1.223	3.80E-02
**IL11**	2.098	cytokine		3.83E-02
**oxaliplatin**		chemical drug		3.83E-02
**sodium arsenite**		chemical drug		3.83E-02
**dibutyl phthalate**		chemical toxicant		3.92E-02
**plumbagin**		chemical toxicant		3.92E-02
**NMU**	-0.622	other		3.92E-02
**ACKR3**	0.593	G-protein coupled receptor		3.92E-02
**BAG1**	0.851	other		3.92E-02
**ochratoxin A**		chemical toxicant		3.92E-02
**kaempferol**		chemical toxicant		3.92E-02
**STAT2**	0.285	transcription regulator		3.99E-02
**CHD4**	0.058	enzyme		3.99E-02
**chloroquine**		chemical drug		3.99E-02
**NR1H2**	0.314	ligand-dependent nuclear receptor	-0.147	4.01E-02
**ID3**	0.454	transcription regulator	1.000	4.06E-02
**ERK1/2**		group	-0.933	4.13E-02
**F2R**	0.158	G-protein coupled receptor	0.152	4.13E-02
**PTF1A**		transcription regulator		4.16E-02
**MUC1**	0.774	other		4.16E-02
**mitomycin C**		chemical drug		4.16E-02
**Ngf**		group		4.16E-02
**SPRY1**	0.700	other		4.21E-02
**PLK2**	0.041	kinase		4.21E-02
**CASR**		G-protein coupled		4.21E-02

		receptor			
**CCNE1**	-1.010	transcription regulator			4.21E-02
**diazoxide**		chemical drug			4.21E-02
**cyanocobalamin**		chemical - endogenous mammalian			4.21E-02
**geranylgeranyl pyrophosphate**		chemical - endogenous mammalian			4.21E-02
**ELAVL1**	-0.233	other	Inhibited	-2.216	4.22E-02
**STAT1**	0.094	transcription regulator		0.798	4.24E-02
**SMAD7**	-0.295	transcription regulator		0.555	4.25E-02
**MAP3K8**	0.078	kinase	Activated	2.219	4.25E-02
**geldanamycin**		chemical - endogenous non-mammalian		-1.342	4.25E-02
**miR-17-5p (and other miRNAs w/seed AAAGUGC)**		mature microRNA			4.34E-02
**E2F6**	-0.200	transcription regulator			4.34E-02
**RHO**		G-protein coupled receptor			4.34E-02
**SIN3A**	-0.091	transcription regulator			4.34E-02
**seocalcitol**		chemical drug			4.34E-02
**SNAI1**		transcription regulator		-1.400	4.35E-02
**caffeic acid phenethyl ester**		chemical drug			4.38E-02
**PRKDC**	-0.493	kinase			4.51E-02
**NOTCH4**	-0.622	transcription regulator			4.51E-02
**Saa3**		other			4.51E-02
**DPP4**	-0.167	peptidase			4.51E-02
**PLK4**	-1.051	kinase			4.51E-02
**diallyl disulfide**		chemical - endogenous non-mammalian			4.51E-02
**ceramide**		chemical - endogenous mammalian			4.51E-02
**dopamine**		chemical - endogenous mammalian		-1.982	4.51E-02
**USF2**	0.197	transcription regulator			4.51E-02
**Ins1**		other		0.086	4.53E-02
**MET**	-0.914	kinase		0.169	4.64E-02
**GLI2**	-0.183	transcription regulator		-0.911	4.64E-02
**PDCD1**		phosphatase			4.69E-02
**25-hydroxycholesterol**		chemical reagent			4.69E-02
**PPARG**	-0.518	ligand-dependent		-0.357	4.69E-02

		nuclear receptor		
**atorvastatin**		chemical drug	0.700	4.75E-02
**alitretinoin**		chemical drug	0.555	4.77E-02
**aspirin**		chemical drug	1.150	4.77E-02
**OSM**		cytokine	1.585	4.78E-02
**picrotoxin**		chemical toxicant		4.82E-02
**apomorphine**		chemical drug		4.82E-02
**FOXL2**	-0.179	transcription regulator		4.87E-02
**NEUROG1**		transcription regulator		4.87E-02
**NRG2**	-0.063	growth factor		4.87E-02
**Go 6976**		chemical - kinase inhibitor		4.87E-02
**prostaglandin E**		chemical - endogenous mammalian		4.94E-02
**flavokawain A**		chemical - endogenous non-mammalian		4.94E-02
**OSGIN1**	-0.149	growth factor		4.94E-02
**ZXDC**	0.185	transcription regulator		4.94E-02
**PROM1**	1.112	other		4.94E-02
**ASCC1**	0.293	transcription regulator		4.94E-02
**SLC13A5**		transporter		4.94E-02
**SLCO1C1**		transporter		4.94E-02
**TEX11**		other		4.94E-02
**ARID2**	0.000	transcription regulator		4.94E-02
**enecadin**		chemical drug		4.94E-02
**ZNF24**	0.217	transcription regulator		4.94E-02
**C1GALT1**	-0.229	enzyme		4.94E-02
**MITF-p300/CBP**		complex		4.94E-02
**sepantronium**		chemical drug		4.94E-02
**firtecan pegol**		chemical drug		4.94E-02
**danusertib**		chemical drug		4.94E-02
**olaparib**		chemical drug		4.94E-02
**TBR1**		transcription regulator		4.94E-02
**ENPP1**	-0.618	enzyme		4.94E-02
**CHFR**	-0.001	enzyme		4.94E-02
**RNF6**	0.041	transcription regulator		4.94E-02
**PAK4**	-0.353	kinase		4.94E-02
**ADRA2B**		G-protein coupled receptor		4.94E-02
**HMG20B**	-0.196	transcription regulator		4.94E-02
**MS4A2**		transmembrane		4.94E-02

		receptor	
**CSH1/CSH2**		other	4.94E-02
**TBX20**		transcription regulator	4.94E-02
**Nppb**		other	4.94E-02
**CHMP2B**	-0.230	other	4.94E-02
**miR-515-5p (and other miRNAs w/seed UCUCCAA)**		mature microRNA	4.94E-02
**miR-202-3p (and other miRNAs w/seed GAGGUAU)**		mature microRNA	4.94E-02
**miR-370-3p (and other miRNAs w/seed CCUGCUG)**		mature microRNA	4.94E-02
**miR-532-3p (miRNAs w/seed CUCCCAC)**		mature microRNA	4.94E-02
**TIAM1**	-0.928	other	4.94E-02
**SSTR5**		G-protein coupled receptor	4.94E-02
**IK**	0.020	cytokine	4.94E-02
**TAF12**	0.039	transcription regulator	4.94E-02
**parbendazole**		chemical reagent	4.94E-02
**DLL3**	-0.138	other	4.94E-02
**HSPB6**		other	4.94E-02
**CANX**	0.370	other	4.94E-02
**ARSA**	0.434	enzyme	4.94E-02
**SIGMAR1**	-0.316	transmembrane receptor	4.94E-02
**DCC**		transmembrane receptor	4.94E-02
**CELF2**	1.075	other	4.94E-02
**GRM3**		G-protein coupled receptor	4.94E-02
**LSM1**	-0.298	other	4.94E-02
**TRPV5**		ion channel	4.94E-02
**RGD1560225**		other	4.94E-02
**GP1BA**	0.452	transmembrane receptor	4.94E-02
**FOXH1**	0.080	transcription regulator	4.94E-02
**ATRX**	0.018	transcription regulator	4.94E-02
**SCTR**		G-protein coupled receptor	4.94E-02
**2-[[4-[(e)-styryl]phenoxy]methyl]oxirane**		chemical reagent	4.94E-02
**S3I-1757**		chemical reagent	4.94E-02
**LGALS2**	-0.234	other	4.94E-02
**TSN**	-0.143	other	4.94E-02
**CEBPZ**	-0.116	transcription regulator	4.94E-02
**Mt3**		other	4.94E-02
**APOC3**		transporter	4.94E-02
**1-[2,3-bis(furan-2-yl)quinoxalin-6-yl]-3-(4-**		chemical reagent	4.94E-02

**bromophenyl)urea**			
**3,6-bis(1-methyl-4-vinylpyridium)carbazole diiodide**		chemical reagent	4.94E-02
**N-hydroxy-2,2-diphenylacetamide**		chemical reagent	4.94E-02
**3,6-bis(4-methyl-2-vinylpyrazinium)carbazole**		chemical reagent	4.94E-02
**CKS2**	-1.418	kinase	4.94E-02
**LY5**		chemical reagent	4.94E-02
**1-(2-hydroxy-5-methylphenyl)-3-phenyl-1,3-propanedione**		chemical reagent	4.94E-02
**abrocitinib**		chemical drug	4.94E-02
**hydrochlorothiazide**		chemical drug	4.94E-02
**TAS-103**		chemical drug	4.94E-02
**pyridoxal phosphate**		chemical - endogenous mammalian	4.94E-02
**nevirapine**		chemical drug	4.94E-02
**liarozole**		chemical drug	4.94E-02
**pyridostigmine**		chemical drug	4.94E-02
**(-)-isoproterenol**		chemical drug	4.94E-02
**ammonia**		chemical - endogenous mammalian	4.94E-02
**chlorophyll a**		chemical - endogenous non-mammalian	4.94E-02
**MS645**		chemical reagent	4.94E-02
**CCI-007**		chemical reagent	4.94E-02
**leucine-2-alanine enkephalin**		chemical drug	4.94E-02
**myristoylated Protein Kinase C peptide inhibitor**		chemical - kinase inhibitor	4.94E-02
**insulin glargine**		biologic drug	4.94E-02
**bisindolylmaleimide II**		chemical - kinase inhibitor	4.94E-02
**limonene**		chemical - endogenous non-mammalian	4.94E-02
**colcemid**		chemical - endogenous non-mammalian	4.94E-02
**trigonelline**		chemical - endogenous mammalian	4.94E-02
**galanthamine**		chemical drug	4.94E-02
**durapatite**		chemical - endogenous mammalian	4.94E-02
**long chain fatty acid**		chemical - endogenous mammalian	4.94E-02
**gemcabene**		chemical drug	4.94E-02
**Collagen type VI**		complex	4.94E-02
**thymeleatoxin**		chemical toxicant	4.94E-02
**karenitecin**		chemical drug	4.94E-02
**Co2+**		chemical - endogenous mammalian	4.94E-02

**TLR7**		transmembrane receptor		-1.982	5.72E-02
**RAS**		group		1.964	8.94E-02
**ADORA2A**		G-protein coupled receptor		1.954	9.32E-02
**imatinib**		chemical drug	Activated	2.042	1.07E-01
**fenofibrate**		chemical drug	Inhibited	-2.390	1.22E-01
**E. coli serotype 0127B8 lipopolysaccharide**		chemical - endogenous non-mammalian		1.982	2.08E-01
**miR-1-3p (and other miRNAs w/seed GGAAUGU)**		mature microRNA		1.980	2.24E-01
**HNF1A**	-1.222	transcription regulator		1.951	2.72E-01
**tazemetostat**		chemical drug	Activated	2.236	3.25E-01
**gentamicin**		chemical drug	Inhibited	-2.000	3.72E-01
**IL10RA**		transmembrane receptor	Activated	2.000	5.55E-01
